# Mechanically Triggered Chemical Recyclable Polyethylene‐Like Materials

**DOI:** 10.1002/anie.202522618

**Published:** 2026-02-01

**Authors:** Menghe Xu, Peng Liu, Changle Chen, Tae‐Lim Choi

**Affiliations:** ^1^ Key Laboratory of Precision and Intelligent Chemistry, Department of Polymer Science and Engineering University of Science and Technology of China Hefei China; ^2^ Department of Materials ETH Zürich Zürich Switzerland

**Keywords:** ball mill, cross‐linked PE, polyethylene recycling, polymer degradation, polymer mechanochemistry

## Abstract

Polyethylene (PE) materials are indispensable to modern infrastructure due to their exceptional thermal, mechanical, and chemical resilience. However, the same properties that make these materials durable also render them environmentally persistent and unrecyclable by conventional means, posing a critical sustainability challenge. Here, we report a mechanochemically triggered, chemically recyclable PE‐like system that enables the closed‐loop recycling of cross‐linked polyethylene (XLPE). Through palladium‐catalyzed coordination copolymerization of ethylene with the cyclobutene‐fused ester (CBE) comonomer, polar PE‐like materials with tunable properties are achieved. Upon optimal mechanical activation in the presence of a radical inhibitor, the CBE units undergo ring opening, installing ester linkages into the polymer backbone. Notably, the high crystallinity of copolymers with low CBE content enables ball‐milling to achieve activation efficiency comparable to cryo‐milling. Subsequent ethanolysis of ester linkages cleanly converts the initial copolymer into multifunctional oligomers, which can be repolymerized after hydrogenation via transesterification to yield a recyclable XLPE with properties comparable to a commercial analogue. This work demonstrates a robust platform for reconciling the durability and recyclability of polyethylene, offering a transformative route toward sustainable polyolefins.

## Graphical abstract

Polyethylene‐like copolymers with benchmark properties are prepared by embedding latent cyclobutene mechanophores to overcome PE's end‐of‐life limits. The mechanophores are inert in service but are selectively opened by solvent‐free ball milling to yield well‐defined telechelic oligomers. After ethanolysis/hydrogenation, these oligomers are repolymerized to cross‐linked PE with commercial‐grade properties, enabling a practical closed‐loop circularity.

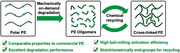

## 1 Introduction

Polyethylene (PE) materials are the most produced synthetic polymer worldwide, with widespread applications in packaging, infrastructure, and consumer products [1, 2, 3]. Its exceptional chemical resistance, mechanical robustness, and processability underpin its utility but also contribute to one of its greatest environmental drawbacks: extreme persistence in the environment. The high stability of C─C and C─H bonds in the PE backbone renders it nearly immune to degradation, leading to the relentless accumulation of plastic waste and posing a major barrier to achieving a circular plastics economy [4, 5, 6, 7].

**SCHEME 1:**
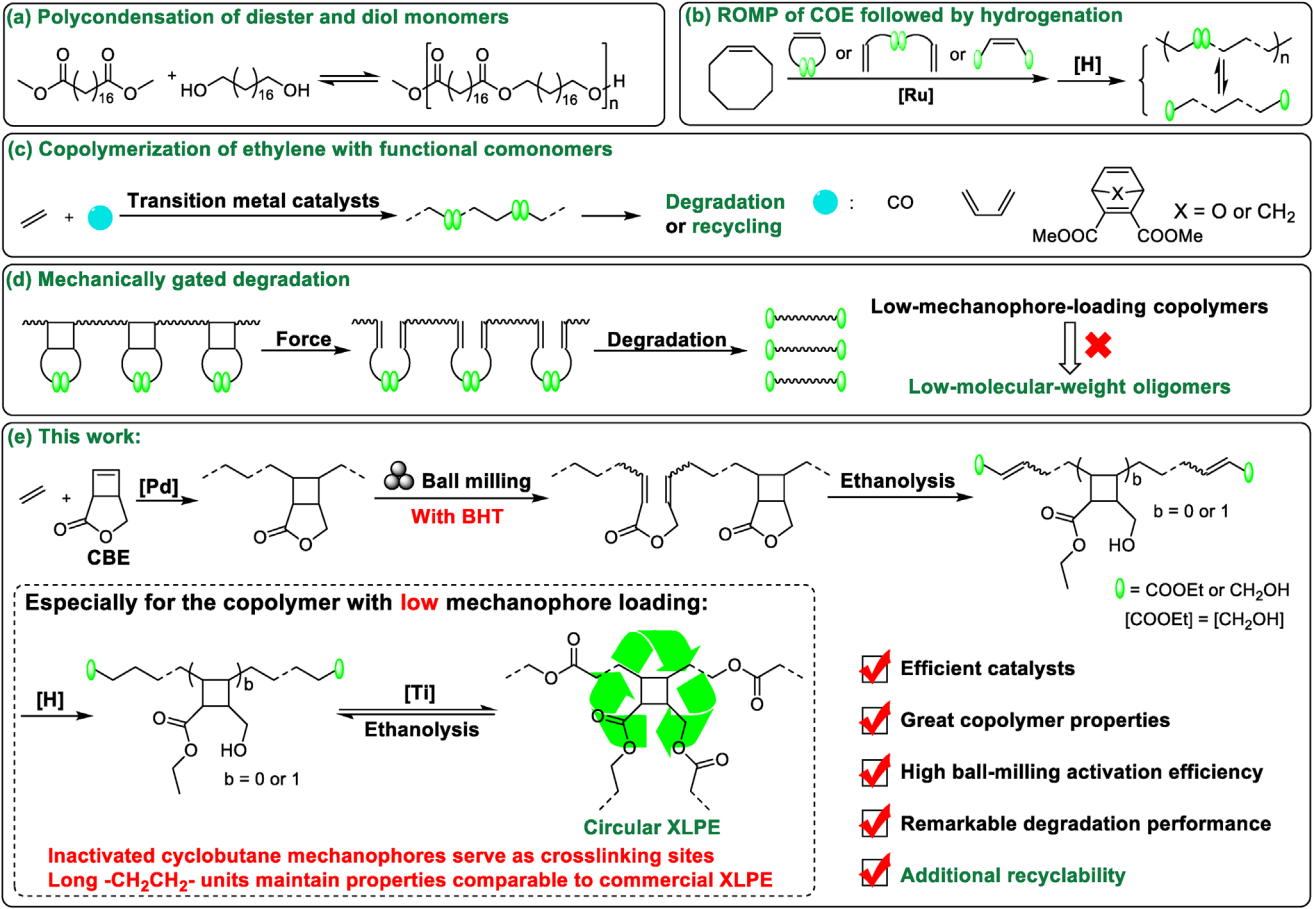
(a–c) Reported approaches to synthesize recyclable and degradable PE‐like materials. (d) Concept of mechanical gated degradation. (e) This work is for mechanically triggered recyclable PE‐like materials.

In recent years, significant efforts have been devoted to degrading/upcycling post‐consumer PE waste by converting it into small molecules or oligomers [8, 9, 10, 11, 12, 13, 14, 15, 16, 17]. Alternatively, a promising approach is the *de novo* design of chemically degradable or recyclable PE‐like materials that preserve the desirable properties of conventional PE while enabling controlled depolymerization under mild conditions. For example, Mecking and co‐workers reported a landmark study on closed‐loop recyclable high‐density PE‐like polymers synthesized via polycondensation of biomass‐derived diester and diol monomers (Scheme 1a) [18, 19, 20, 21]. Moreover, ring‐opening olefin metathesis polymerization (ROMP) of cyclooctene (COE) in the presence of functional cyclic olefins [22, 23, 24], *α*,*ω*‐dienes [25, 26], or chain‐transfer agents (CTAs) [27, 28, 29, 30], followed by hydrogenation, can realize the tailored synthesis of circular PE‐like materials (Scheme 1b). In comparison, directly copolymerizing ethylene with functional comonomers represents an ideal and efficient approach to synthesizing degradable PE‐like materials (Scheme 1c). For instance, keto‐PE from the nonalternating copolymerization of ethylene and carbon monoxide undergoes degradation into smaller fragments upon UV irradiation [31, 32, 33] or main‐chain editing [34, 35]. Furthermore, copolymerization of ethylene with butadiene [36] or retro‐Diels–Alder cyclic olefins [37, 38] can introduce unsaturated double bonds in the polymer backbone, serving as cleavable sites for subsequent degradation and recycling. However, these in‐chain cleavable bonds often compromise the long‐term stability of polymers, as they are prematurely activated by thermal, chemical, or photolytic stimuli during initial service.

In contrast, the concept of mechanically gated degradation has emerged as a promising solution, in which degradable units remain inert during normal use and are only activated by applied mechanical force (Scheme 1d) [39]. Pioneering studies by Craig [40], Wang [41], Xia [42], and others established this concept by embedding mechanophores into tailored polymer chains to enable on‐demand degradation. More recently, Liu and Bruns extended this strategy to commodity polymers with saturated hydrocarbon backbones, including polystyrene, poly(methyl acrylate), poly(methyl methacrylate), and styrene‐butadiene rubber, via radical copolymerization of the corresponding monomers with cyclobutane imide (CBI) mechanophores [43]. The obtained polymers can achieve on‐demand mechanically activated degradation followed by hydrolysis. Tang and co‐workers further introduced this strategy into PE with in‐chain mechanophores using cyclobutene‐fused comonomers, which demonstrated the first mechanically triggered on‐demand degradable PE [44, 45, 46]. These mechanically gated degradation systems primarily targeted the copolymers with high mechanophore incorporation, which were readily degraded into low‐molecular‐weight oligomers [44]. However, the low‐mechanophore‐loading copolymers, which exhibit properties closer to commercial analogues and warrant greater attention, proved difficult to degrade effectively due to the inevitably incomplete and nonrandom mechanical activation, as well as poor solubility. Moreover, the resulting degradation products are typically heterogeneous and poorly defined with broad distribution (*Đ>30*), limiting their feasibility for closed‐loop recycling [45]. It should be noted that these residual polar, moderate‐molecular‐weight PE fragments (>5 kDa) are resistant to biodegradation [12], thereby presenting persistent environmental hazards. Therefore, more systematic studies are desired to investigate the structure‐property‐degradation relationship and to obtain optimal degradation conditions, as well with potential to achieve chemical recyclability.

In this work, we attempt to enhance the practicality of the mechanically gated degradation strategy by combining it with chemical recyclability, thereby overcoming these limitations (Scheme 1e). A series of PE‐like materials were efficiently prepared from the copolymerization of ethylene with a mechano‐active lactone, 3‐oxabicyclo[3.2.0]hept‐6‐en‐2‐one (CBE), using the selected phosphine–sulfonate palladium catalysts, and their properties were highly tunable to match those of commercial PE. The ester sites, which are initially installed as side‐chain pendants, are chemically latent under service conditions but can be transformed into the polymer backbone via mechanical activation, thereby undergoing selective cleavage upon subsequent ethanolysis. Through optimizing ball‐milling conditions and adding butylated hydroxytoluene (BHT) to suppress undesired radical‐induced side reactions, high mechanical activation efficiency was achieved, thereby efficiently facilitating the subsequent degradation. Remarkably, this then led to a product that consisted of an equal amount of hydroxyl and ester groups that could be efficiently repolymerized via trans‐esterification after hydrogenation. The resulting cross‐linked PE (XLPE) retained properties comparable to commercial analogue and could be recycled back to the original multifunctional PE fragments upon ethanolysis. This approach thus enables a closed‐loop lifecycle for high‐performance PE and represents a significant advance in sustainable materials design.

## 2 Results and Discussion

### 2.1 Catalytic Copolymerization of Ethylene and CBE

To enable mechanically triggered degradation and subsequent chemical recyclability, we selected CBE as the functional comonomer. CBE is a multifunctional monomer designed to fulfill several key roles: the strained alkene facilitates incorporation into the polyethylene backbone via coordination–insertion polymerization; the cyclobutane moiety functions as a latent mechanophore that enables mechanically triggered activation; and the embedded ester functionality provides chemical degradation and repolymerization. The other key aspect is the choice of a suitable catalyst system, which can efficiently copolymerize ethylene with CBE. Herein, the phosphine–sulfonate palladium catalysts, Pd1‐Pd3, were employed due to their outstanding polar functional tolerance [47]. Based on previous reports, Pd1 [48] bearing a tertiary butyl group can afford copolymers with both high molecular weights and high incorporation ratios. By comparison, the sterically bulky biaryl‐based Pd2 [49] can generate high‐molecular‐weight copolymers with low incorporation ratios, while the dianisyl‐substituted Pd3 [50] can insert large amounts of comonomers at the expense of molecular weights.

**FIGURE 1:**
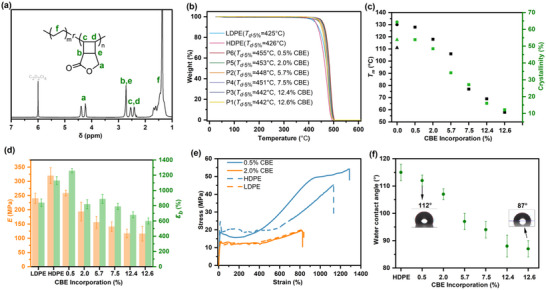
Characterizations of synthesized and commercial PE. (a) ^1^H NMR of P1. (b) TGA traces and decomposition temperature (*T_d_*) of all synthesized PE‐like polymers and commercial HDPE and LDPE at 5% decomposition. (c) Melting temperature (*T_m_*, black points) and crystallinity (*X_c_*, green points) of all synthesized PE‐like polymers (square) and commercial HDPE (circle) and LDPE (triangle). (d) Young's modules (orange) and elongations at break (green) of all synthesized PE‐like polymers and commercial HDPE and LDPE. (e) Strain‐Stress curves of P5 (solid orange line), P6 (solid blue line), commercial HDPE (dashed blue line), and LDPE (dashed orange line). (f) Water contact angles of all synthesized PE‐like polymers.

Firstly, copolymerizations were conducted using 8 atm of ethylene and 1.0 mol·L^−^^1^ of CBE in chlorobenzene at 80 °C for 30 min using Pd1‐Pd3 (Table 1, P1‐P3), which showed similar moderate catalytic activities (3.4 × 10^5^ – 4.2 × 10^5^ g·mol^−1^·h^−1^). As expected, the copolymer P1 generated from Pd1 possessed a high molecular weight (*M*_n_) of 82 kDa, and a high incorporation ratio (*F_CBE_*) of 12.6 mol% (Table 1, P1). The successful incorporation of CBE was confirmed by ^1^H NMR spectroscopy, with characteristic resonances observed at 4.32 ppm (H_a_), 2.72 ppm (H_b_ and H_e_), 2.55 ppm (H_c_), and 2.42 ppm (H_d_) (Figure 1a). Compared to Pd1, Pd2 formed a copolymer with a slightly lower *M*_n_ of 62 kDa and a reduced *F_CBE_* of 5.7 mol% (Table 1, P2). In contrast, Pd3 produced a copolymer with a similar *F_CBE_* of 12.4 mol% to P3, but its *M*_n_ was significantly decreased to 26 kDa (Table 1, P3). This is meaningful to investigate the effect of *M*_n_ on the mechanical properties and the mechanochemical reactivity of polymers in the subsequent experiments. Notably, the *M*_n_ of P3 was higher than that of PE synthesized under analogous conditions using Pd3 [50]. This suggests that the cyclobutene‐fused structure can suppress the chain transfer process by hindering the β‐hydride elimination reaction during the copolymerization, similar to the behavior observed with norbornene‐based monomers [51]. Furthermore, copolymerization parameters were optimized to reduce the CBE content in the copolymer with a suitable polymer molecular weight, achieving properties approaching those of commercial PE. This is achieved by simply decreasing the CBE concentration, thereby increasing catalytic activities and *M*_n_ (Table 1, P1 vs. P4, P2 vs. P5). For example, Pd2 provided P5 with a sharply increased catalytic activity (2.5 × 10^6^ g·mol^−1^·h^−1^) and a slightly increased *M*_n_ (65 kDa) at 0.2 mol·L^−^^1^ of CBE despite showing a lower *F*_CBE_ of 2.0 mol% (Table 1, P5). Moreover, P6 with the lowest *F_CBE_* of 0.5 mol% and the highest *M*_n_ of 190 kDa was prepared by Pd2 at 60 °C, along with a low catalytic activity (Table 1, P6).

**TABLE 1:** Catalytic copolymerization of ethylene with CBE.^a^

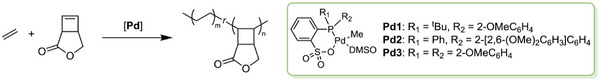								
Polymer	Cat. [Pd] (*µ*mol)	CBE (mol·L^−1^)	Time (min)	Yield (g)^b^	Activity^b^ (10^4^ g·mol^−1^·h^−1^)	*F*_CBE_ (%)^c^	*M*_n_ (kDa)^d^	*Đ* ^d^
P1	Pd1(10)	1.0	30	1.7	34	12.6	82	1.9
P2	Pd2(10)	1.0	30	1.7	34	5.7	62	2.0
P3	Pd3(10)	1.0	30	2.1	42	12.4	26	1.9
P4	Pd1(10)	0.5	30	2.1	42	7.5	90	2.6
P5	Pd2(5)	0.2	15	3.1	250	2.0	65	2.6
P6^e^	Pd2(20)	1.0	420	8.8	6	0.5	190	2.6

^a^ Polymerization conditions: total volume of chlorobenzene and comonomer = 19 mL, palladium catalyst in 1 mL of CH_2_Cl_2_, polymerization temperature = 80 °C, ethylene pressure = 8 atm.

^b^ The yields and activities are the averages of at least two runs.

^c^ CBE incorporation ratio (*F*_CBE_) was determined by ^1^H NMR spectroscopy in C_2_D_2_Cl_4_ at 120 °C.

^d^ Molecular weight (*M*_n_) and dispersity (*Đ*) were determined by SEC in 1,2,4‐trichlorobenzene at 150 °C.

^e^ Total volume of chlorobenzene and comonomer = 49 mL, polymerization temperature = 60 °C.

### 2.2 Polymer Properties

The thermal, mechanical, and surface properties of the copolymers were systematically evaluated and benchmarked against commercial high‐density PE (HDPE) and low‐density PE (LDPE) reference materials. Thermogravimetric analysis (TGA) revealed that all synthesized copolymers, with *F*_CBE_ ranging from 0.5 mol% to 12.6 mol%, exhibited excellent thermal stability. Specifically, the temperature at 5% weight loss (*T*_d,5%_) exceeded 442 °C for all copolymers, surpassing those of commercial HDPE (*T*_d,5%_ = 426 °C) and LDPE (*T*_d,5%_ = 425 °C) (Figure 1b). This enhancement underscores the thermal robustness imparted by the CBE comonomer units.

Differential scanning calorimetry (DSC) confirmed that all copolymers remain semi‐crystalline. Notably, P6, with the lowest *F*_CBE_ (0.5 mol%), displayed a melting temperature (*T*_m_) of 128 °C higher than that of LDPE (*T*_m_ = 111 °C) and comparable to HDPE (*T*_m_ = 132 °C). The degree of crystallinity (*X*_c_) of P6 reached 53.9%, nearly identical to commercial LDPE (*X*_c_ = 53.8%). As expected, increasing the CBE content progressively disrupted the crystalline packing of the PE chains due to the bulky and rigid cyclobutane structures, leading to reduced *T*_m_ and *X*_c_ values (Figures 1c and S71–S78). Nevertheless, we identify a threshold around 5.7 mol% CBE incorporation: copolymers at or below this incorporation retain a melting temperature above 106 °C and crystallinity exceeding 34.2%, values that remain comparable to many commercial PE grades (typically *T*_m_ > 100 °C, *X*_c_ > 30%).

Tensile testing revealed that both *F*_CBE_ and *M_n_* significantly influence mechanical performance (Table S1). For instance, P1 (*F*_CBE_ = 12.6 mol%, *M_n_* = 82 kDa) exhibited a high tensile strength (*σ*_b_ = 53.8 ± 2.1 MPa), substantially outperforming P3 (*σ*_b_ = 33.1 ± 1.1 MPa), which has a similar *F*_CBE_ of 12.4 mol% but a much lower *M*_n_ of 26 kDa. Comparatively, P2 (*F*_CBE_ = 5.7 mol%, *M*_n_ = 62 kDa) showed a lower Young's modulus (*E* = 156 ± 21 MPa) than P5 (*E* = 194 ± 33 MPa), which possesses a lower *F*_CBE_ of 2.0 mol% but a comparable *M*_n_ of 65 kDa. P6, with the highest *M*_n_ of 190 kDa and minimal *F*_CBE_ of 0.5 mol%, demonstrated the best overall mechanical performance, combining high ductility (strain at break *ε*_b_ = 1260 ± 30%), strength (*σ*_b_ = 53.3 ± 1.2 MPa) and stiffness (*E* = 259 ± 10 MPa) (Figure 1d), values approximately twice those reported previously [45]. Remarkably, P5 displayed a stress–strain profile nearly identical to that of commercial LDPE, while P6 closely matched the tensile behavior of commercial HDPE (Figure 1e). These observations suggest that high *M*_n_ and low *F*_CBE_ are critical for achieving optimal mechanical properties, which weren't achieved in earlier work. More broadly, the series of copolymers exhibits tunable mechanical properties, on par with or exceeding those of commercial PEs (Figure S1).

Given the presence of ester functionalities, the PE‐like copolymers are expected to exhibit higher surface polarity than conventional PE. Water contact angle (WCA) measurements were performed to evaluate surface hydrophilicity. As anticipated, the incorporation of polar CBE units led to a progressive decrease in WCA from 115 ° (commercial HDPE, *F*_CBE_ = 0 mol%) to 87 ° for P1 (*F*_CBE_ = 12.6 mol%), indicating enhanced surface polarity with increasing CBE content (Figure 1f).

### 2.3 Mechanical Degradation of Copolymers

**FIGURE 2:**
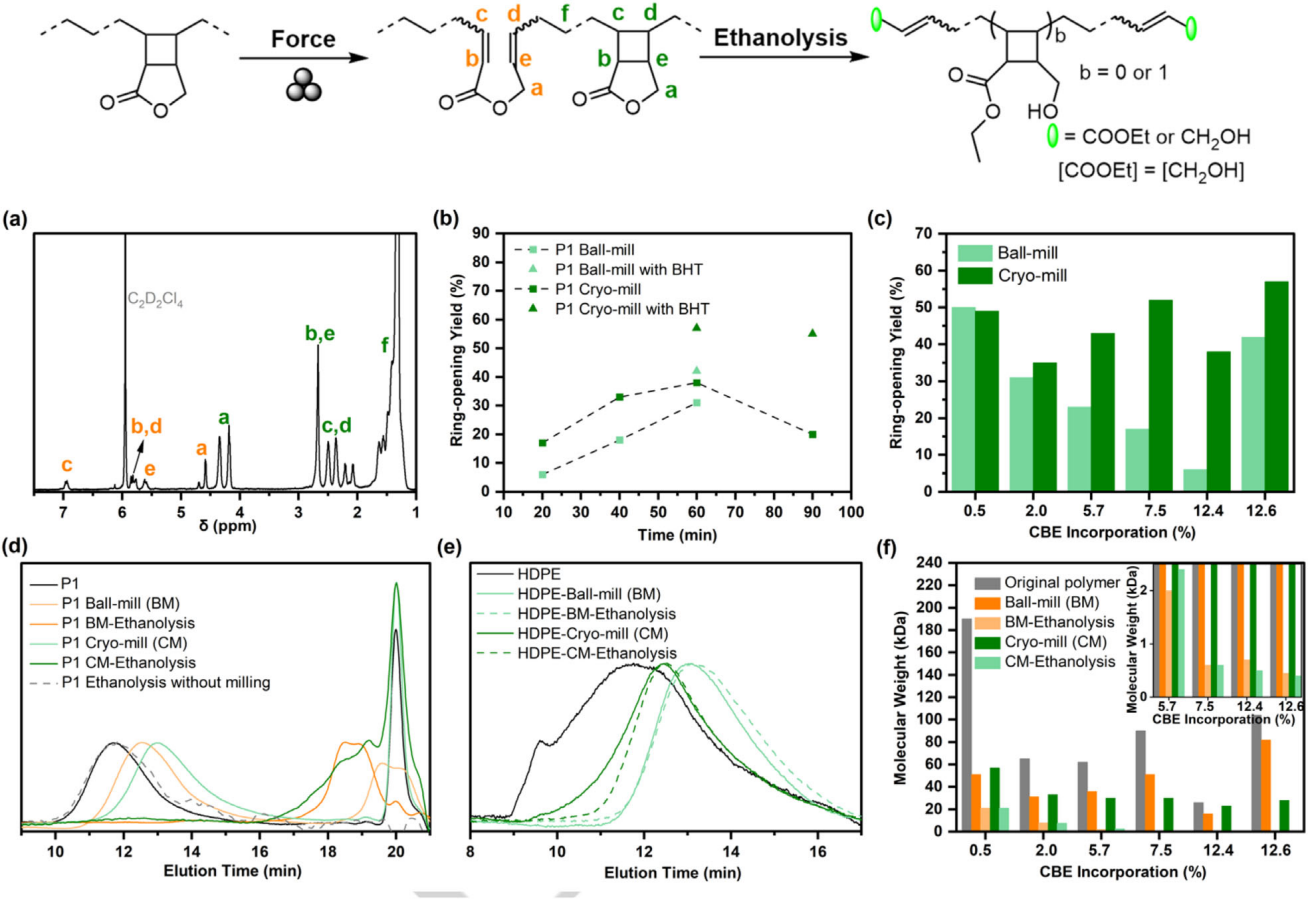
Mechanical activations and degradations of PE‐like polymers and commercial HDPE, LDPE. (a) ^1^H NMR of P1 after cryo‐milling for 20 min. (b) Cyclobutane mechanophore activation condition optimization. (c) Cyclobutane mechanophore ring‐opening yields with different CBE incorporations. (d) SEC traces of pristine P1, P1 after milling, P1 after milling and ethanolysis, and P1 after ethanolysis but without milling. (e) SEC traces of commercial HDPE, the HDPE after milling, the HDPE after milling and ethanolysis. (f) Molecular weights of the original polymers, the polymers after milling, and the polymers after milling and ethanolysis. When the CBE content exceeds 5.7 mol%, the molecular weights of the final degraded fractions drop below 1 kDa, as shown in the small inset at the top right corner of the panel. Except for the P1 ball‐mill and P1 cryo‐mill experiments in 2b, where no BHT was added, and unless otherwise noted, all milling experiments were performed with 10 wt% BHT at 30 Hz for 60 min total milling time using 5 min on/5 min off cycles.

The mechanochemical reactivity of the CBE‐containing copolymers was investigated under solid‐state conditions using practical ball milling (BM) at room temperature, without heating or solvents. Samples were subjected to vibrational milling in a stainless steel jar equipped with two stainless steel balls at 30Hz. After 20 min of BM, ^1^H NMR analysis of P1 revealed four new resonance signals (Figure 2a). The peaks at 7.00 ppm (H_c_, orange) and 5.90 ppm (H_b_, orange) were assigned to *α*,*β*‐unsaturated ester protons, while those at 5.82 ppm (H_d_, orange) and 5.67 ppm (H_e_, orange) were attributed to another alkene protons, all of which were formed upon cyclobutane ring opening. Additionally, a downfield shift of the ‐CH_2_‐ resonance from 4.30 ppm (green H_a_) to 4.69 ppm (orange H_a_) further supported the mechanochemical ring‐opening of the cyclobutane mechanophores. Initially, the ring‐opening yield after 20 min of BM was limited to 6%, while extending the milling time to 60 min increased the conversion to 31% (Table S2). However, prolonged milling led to the formation of insoluble material, presumably due to undesired radical‐induced cross‐linking and other side reactions on the generated *α*,*β*‐unsaturated esters, thereby lowering the apparent ring‐opening yield. To suppress such side reactions, cryogenic milling (CM) was performed. While CM increased the ring‐opening performance to 38%, crosslinking still remained evident (Table S2). To mitigate this, 10 wt% of the radical scavenger butylated hydroxytoluene (BHT) was added during milling, which subsequently increased the performance to 57% after 60 min of CM (Figure 2b), or to 42% under BM. Different ball‐milling frequencies were also evaluated, showing no ring opening at 10 Hz, whereas increasing the frequency to 20 Hz afforded only a 15% ring‐opening yield (Table S3).

Mechanochemical activation studies were further extended to copolymers P2–P6 and commercial HDPE and LDPE as control experiments under identical conditions (30 Hz, 60 min milling with 10 wt% BHT; Figure 2c). All CBE‐containing copolymers exhibited evidence of cyclobutane ring opening and molecular weight reduction, while no alkene resonances were detected in the spectra of mechanically treated commercial HDPE or LDPE, indicating the absence of mechanophore activation in those materials (Table S4). For copolymers P2–P4, CM consistently yielded much higher ring‐opening efficiencies than conventional BM. This difference was particularly pronounced in P3 (*M*_n_ = 26 kDa, *F*_CBE_ = 12.4 mol%), where CM afforded a 38% yield versus 6% from BM, suggesting that *M*_n_ strongly influences mechanochemical responsiveness. More importantly, no notable difference was observed between the BM and CM methods for copolymers with low CBE incorporation (P5 and P6). This can be explained that the crystallization of PE segments significantly enhanced the activation of cyclobutane mechanophores at room temperature. In this regime, chain segments bearing cyclobutane mechanophores are largely confined to the amorphous regions and effectively tethered between crystalline lamellae, which restricts segmental mobility and suppresses stress dissipation under room‐temperature milling. This constraint can mimic the “glassy” response typically achieved at cryogenic temperatures, thereby enhancing force transduction to the mechanophore. Alongside mechanophore activation, nonspecific chain scission, an intrinsic feature of mechanical polymer activation, was also observed. For example, 60 min of milling of P1 (*M*_n_ = 82 kDa) resulted in lower *M*_n_ of 49 kDa (BM) or *M*_n_ of 28 kDa (CM). Similar trends were observed for the other copolymers and for commercial PE materials (Table S4). Notably, the ring‐opening efficiencies are significantly higher than prior reports, with a maximum value reaching 57% after 1 h, which is more than twice that of the previous maximum value using cryo‐milling for 2 h (23%) [45], underscoring the efficiency of our method.

To assess the degradability of the mechanochemically activated polymers, ethanolysis was performed as a model degradation reaction. Following BM or CM, the samples were treated with triazabicyclodecene (TBD) in ethanol/xylene (v:v = 1:4) at 120 °C for 12h. Size exclusion chromatography (SEC) of the treated P1 samples revealed the appearance of a low‐molecular‐weight species (*M_n_* < 1 kDa), consistent with successful backbone cleavage (Figure 2d). In contrast, commercial HDPE and LDPE samples, which also exhibited slight molecular weight reductions after BM or CM, showed no evidence of low‐molecular‐weight products after ethanolysis (Figure 2e and Figure S2), confirming that polymer degradation is specific to the mechanochemically activated CBE‐containing copolymers.

Control experiments verified that P1 exposed to ethanolysis conditions without mechanical activation did not degrade (Figure 2d, dashed line), further underscoring the stability of the copolymers under thermal and chemical conditions alone. Following sequential mechanical activation and ethanolysis, all CBE‐containing copolymers (P1–P6) underwent varying degrees of degradation, consistent with selective and tunable degradation (Figure 2f, S43–S58, and Table S4). Notably, P3 (*M*_n_ = 26 kDa; *F*_CBE_ = 12.4 mol%) was deconstructed to oligomers with *M*_n_ < 1.0 kDa, a transformation that is difficult to achieve for low *M*_n_ PE by conventional routes. The resulting oligomer streams display markedly reduced dispersities (*Đ* = 1.2–7.2) relative to the prior reports (*M*_n_ = 1.3 kDa, *Đ* = 31–62), underscoring the selectivity of this approach and its suitability for closed‐loop recycling, which wasn't accessible by previous reports [44, 45]. ^1^H NMR and MALDI‐TOF analysis of the degradation products revealed the presence of small molecules corresponding to the expected sequence of cyclobutane ring opening followed by ester bond cleavage (Figures S3–S6), thereby validating the proposed degradation mechanism. Moreover, the identical ^1^H NMR and mass spectra of the products obtained after sequential ball milling or cryo‐milling, followed by hydrogenation and ethanolysis, indicate that both methods generate the same products via the same degradation pathway.

### 2.4 Recyclability of Degradation Products

Conventional XLPE is a widely used thermoset plastic, but it lacks circularity due to its permanent cross‐links [52]. Introducing dynamic covalent bonds into XLPE to enable reprocessability and multiple use cycles has become a mainstream strategy to enhance recyclability [53, 54, 55, 56, 57]. On the other hand, fully recyclable XLPE‐like materials remain scarce [58, 59]. Therefore, we aimed to develop a new class of XLPE‐like materials with improved recyclability, in which the multifunctional macromonomers obtained from degradation could undergo subsequent polymerization and depolymerization back to initial macromonomers. Notably, the degradation products from the low‐CBE‐incorporation copolymers P6 (*M*_n_ = 190 kDa, *F*_CBE_ = 0.5 mol%) showed significant potential toward new XLPE‐like materials, because their long ‐CH_2_CH_2_‐ units enabled them to maintain properties comparable to commercial XLPE. Furthermore, additional advantages include: the inherently stoichiometrically balanced ester and hydroxyl groups are favorable to polymerization; the inactivated cyclobutane mechanophores can serve as crosslinking sites.

**FIGURE 3:**
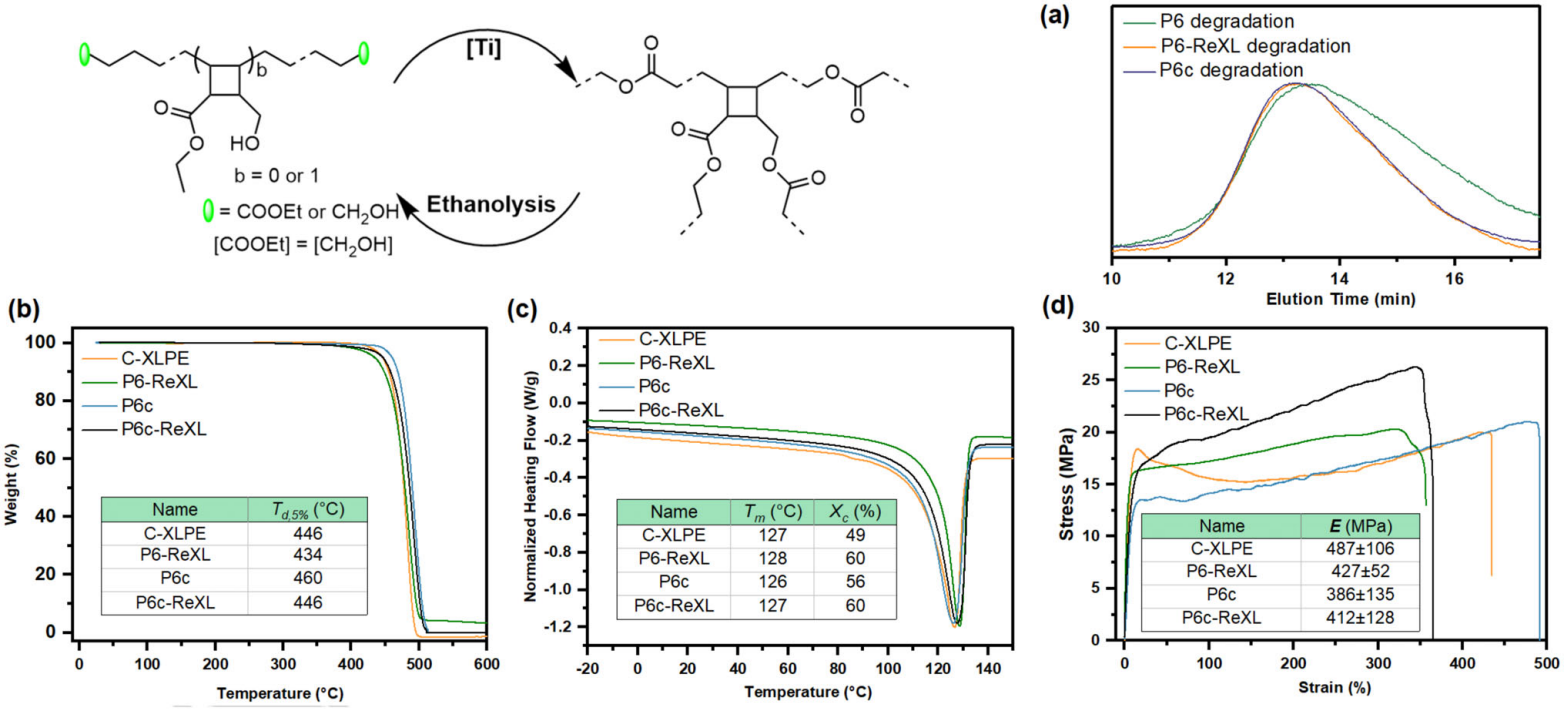
Comparison between C‐XLPE, P6‐ReXL, P6c, and P6c‐ReXL. (a) SEC traces of P6, P6‐ReXL, and P6c‐ReXL degradation fractions by sequential ball mill, hydrogenation, and ethanolysis. (b) TGA traces and decomposition temperature (*T_d_*) of C‐XLPE, P6‐ReXL, P6c, and P6c‐ReXL at 5% decomposition. (c) DSC traces, melting temperature (*T_m_*), and crystallinity (*X_c_*) of C‐XLPE, P6‐ReXL, P6c, and P6c‐ReXL. (d) Strain‐ Stress curves, and Young's modules of C‐XLPE, P6‐ReXL, P6c, and P6c‐ReXL.

Initially, we attempted to repolymerize the degradation product from P6 with titanium *n*‐butoxide (Ti(O*^n^* Bu)_4_) as a catalyst via transesterification under neat conditions at 180 °C and high vacuum to remove the byproduct ethanol. However, uncontrolled crosslinking occurred presumably due to the thermal instability of alkene groups in the degraded fragments, toward crosslinking by further radical addition. To circumvent this issue, the degraded fractions were hydrogenated first to eliminate residual alkene functionalities (Supplementary Information, page 7). Polymerization of the resulting products successfully yielded XLPE (P6‐ReXL). Crucially, P6‐ReXL can be degraded again by ethanolysis into initial oligomers (Figure 3a), demonstrating a good circular lifecycle.

To investigate the properties of P6‐ReXL, a commercial HDPE‐based XLPE (C‐XLPE) was selected as the control sample. TGA revealed that P6‐ReXL possessed a decomposition temperature (*T*_d_ = 434 °C), slightly lower than that of C‐XLPE (*T*_d_ = 446 °C), likely due to the inherent lower thermal stability of ester linkages within the polymer network (Figure 3b and S67, S68). DSC showed similar melting temperatures for P6‐ReXL and C‐XLPE (*T*_m_ = 128 °C vs. 127 °C, respectively). However, the crystallinity of P6‐ReXL (*X*_c_ = 60%) was higher than that of C‐XLPE (*X*_c_ = 49%) (Figures 3c and S79, S80). Meanwhile, P6‐ReXL exhibited good mechanical properties on par with C‐XLPE (Figure 3d). Moreover, the gel fraction of P6‐ReXL was higher than that of C‐XLPE (75.1% vs. 66.7%). In short, P6‐ReXL with closed‐loop life‐cycle retained properties comparable to commercial XLPE.

A key challenge for the chemical recycling of XLPE is its poor solubility, which severely limits conventional solution‐phase depolymerization approaches. To address this limitation and further demonstrate the advantages of our method, another cross‐linked PE (P6c) was prepared via transesterification of P6 with 1,12‐dodecanediol in the presence of Ti(O*^n^* Bu)_4_ at 180 °C under Ar. Notably, P6c exhibits a higher decomposition temperature (*T*_d_ = 460 °C; Figure 3b) than other XLPEs, which is likely attributed to the cyclobutane mechanophore that can dissipate energy through ring opening prior to thermal decomposition. Importantly, its melting behavior, crystallinity, and mechanical properties remain comparable to those of conventional XLPEs (Figures 3c, 3d, and S81). Most significantly, P6c can be degraded on demand into low‐molecular‐weight fractions through a sequence of solvent‐free ball milling, hydrogenation, and ethanolysis (Figure 3a). The resulting fractions were then repolymerized in the presence of Ti(O*^n^* Bu)_4_ at 180 °C under vacuum to afford a regenerated cross‐linked PE (P6c‐ReXL), which again displays comparable decomposition temperature, melting behavior, crystallinity, and mechanical properties (Figures 3a–3d and S82). Collectively, these results establish a practical closed‐loop chemical recycling strategy for cross‐linked PE that bypasses solubility constraints while maintaining performance metrics comparable to commercial XLPE.

## 3 Conclusion

In summary, we present a strategy for designing polyethylene‐based materials that combine the durability of conventional PE with on‐demand degradability and chemical recyclability. By incorporating cyclobutane‐fused esters into the polymer backbone via ethylene and CBE copolymerization, we access PE‐like copolymers with tunable composition and molecular weight. These materials exhibit tunable thermal and mechanical properties that are comparable to the commercial PE, but can be selectively activated with excellent efficiency through ball‐milling mechanochemistry to introduce cleavable ester units, even at low molecular weight and low CBE incorporation. Subsequently, hydrogenation and ethanolysis yield well‐defined telechelic oligomers with narrow distribution, which can be repolymerized into crosslinked polyethylene with properties rivaling commercial XLPE. Importantly, this XLPE is chemically recyclable, enabling a closed‐loop life cycle. This approach offers a promising route toward circular high‐performance polyolefins.

## Conflicts of Interest

The authors declare no conflicts of interest.

## Supporting information



The data that support the findings of this study are available in the Supporting Information of this article. The authors have cited additional references within the Supporting Information.

## Data Availability

The data that support the findings of this study are available in the supplementary material of this article.
