# Supplementary material for: Mechanically Triggered Chemical Recyclable Polyethylene‐Like Materials
Source: Angew Chem Int Ed Engl. 2026 Feb 1;65(11):e22618. doi: 10.1002/anie.202522618 (PMC12970492; doi:10.1002/anie.202522618)
Supplement: Supplementary file 1 — The data that support the findings of this study are available in the Supporting Information of this article. The authors have cited additional references within the Supporting Information. [file ANIE-65-e22618-s001.docx]

**Supplementary Information**

**Mechanically Triggered Chemical Recyclable Polyethylene-like Materials**

Menghe Xu ^‡,a^, Peng Liu ^‡,b,^* Changle Chen^a,^*, Tae-Lim Choi^b,^*

*^a^ CAS Key Laboratory of Soft Matter Chemistry, Department of Polymer Science and Engineering, University of Science and Technology of China, Hefei, China*

*^b^ Department of Materials, ETH Zürich, Vladimir-Prelog-Weg 1-5/10, 8093 Zürich, Switzerland*

^‡^ These authors contributed equally.

* Email: liupeng@ethz.ch; changle@ustc.edu.cn; tlc@ethz.ch

Table of Contents

[General experimental details and methods 1](#_Toc201313115)

[Ethylene and CBE copolymerization 2](#_Toc201313116)

[Mechanochemical activation of copolymers 3](#_Toc201313117)

[Degradation of mechanical activated copolymers 4](#_Toc201313118)

[Repolymerization of the degraded fractions 6](#_Toc201313119)

[^1^H NMR of copolymers 8](#_Toc201313120)

[^1^H NMR of mechanical activated copolymers 11](#_Toc201313121)

[SEC of copolymers 14](#_Toc201313122)

[SEC of mechanical activated polymers 18](#_Toc201313123)

[SEC of degraded polymers 26](#_Toc201313124)

[TGA of copolymers 34](#_Toc201313125)

[DSC of copolymers 40](#_Toc201313126)

[Reference 46](#_Toc201313127)

# **General experimental details and methods**

Maleic anhydride, (*E*)-1,2-dichloroethene, acetic anhydride (Ac_2_O), Zinc (Zn) dust, sodium borohydride (NaBH_4_), anhydrous magnesium sulfate (MgSO_4_) were purchased from Sinopharm Chemical Reagent Co., Ltd. Hydrogen chloride (HCl) was purchased from Sinopharm Chemical Reagent Co., Ltd. and diluted with distilled water before using. Butylated hydroxytoluene (BHT), tripropylamine (TPA), triazabicyclodecene (TBD), *trans*-2-[3-(4-tert-Butylphenyl)-2-methyl-2-propenylidene]malononitrile (DCTB) and titanium *n*-butoxide (Ti(O*^n^*Bu)_4_) were purchased from Sigma-Aldrich. *p*-Toluenesulfonyl hydrazide (PTSH) was purchased from Fluorochem. Chloroform-*d* (CDCl_3_), 1,1,2,2-tetrachloroethane-*d_2_* (C_2_D_2_Cl_4_), xylene, ethanol (EtOH), methanol (MeOH), and 1,2,4-trichlorobenzene were purchased from Fisher Scientific. High density polyethylene (HDPE, TR480) was purchased from Shanghai Jinfei Petrochemical Co., Ltd. Low density polyethylene (LDPE, 2426H) was purchased from China Shenhua Energy Co., LTD. Crosslinked polyethylene (XLPE) was purchased from Polidan T/A-HF. Reagents from commercial sources were used without further purification unless otherwise stated. Anhydrous dichloromethane (CH_2_Cl_2_), tetrahydrofuran (THF), chlorobenzene, and toluene were obtained from a Pure Process Technology solvent purification system. All manipulations of air- and water-sensitive compounds were carried out using standard Schlenk, high-vacuum, and glovebox techniques under nitrogen. **Pd1**^1^, **Pd2**^2^, **Pd3**^3^ and 3-oxabicyclo[3.2.0]hept-6-en-2-one (**CBE**)^4^ were synthesized according to literatures.

^1^H NMR spectra were recorded on a Bruker Ascend 500 MHz spectrometer. The chemical shifts of the ^1^H NMR spectra are referenced to the residual proton resonance of chloroform-*d*(CDCl_3_: δ: 7.26 ppm) or 1,1,2,2-tetrachloroethane-*d_2_* (δ: 6.00 ppm). The measurements of small molecules were performed in chloroform-d at 25 °C. The measurements of polymers were performed in 1,1,2,2-tetrachloroethane-*d_2_* at 120 °C. Polymer samples were preheated for at least 20 min before acquiring data.

Size exclusion chromatography (SEC) analyses of the polyethylene copolymers were carried out with a PL-220 system equipped with two Agilent PLgel Olexis columns, and a refractive index (RI) detector at 150 °C using 1,2,4-trichlorobenzene (100 ppm BHT as stabilizer) as mobile phase at a flow rate of 1.0 mL/min. Molecular weights and molecular weight distributions were determined by means of SEC using narrow polystyrene standards.

Matrix-Assisted Laser Desorption/Ionization Time-of-Flight Mass Spectrometry (MALDI-TOF MS) Analyses were performed on a Bruker Auto Flex MaX instrument (positive reflex mode). DCTB was used as the matrix.

Thermal gravimetric analysis (TGA) was obtained using a Waters Discovery TGA 5500 analyzer. Analysis was performed on 2-10 mg of sample at a heating rate of 10 °C /min from 30 °C to 700 °C under nitrogen gas.

Differential Scanning Calorimetry (DSC) measurements were performed on a Waters Discovery DSC 2500 analyzer. Samples (ca. 5 mg) were annealed at a heating and cooling rate 10 °C /min in a range of -80 °C to 180 °C at 10 °C/min. Melting temperature (*T_m_*) and glass transition temperature (*T_g_*) values were obtained from the second heating scan. The crystallinity of the copolymer was calculated using its measured

Δ*H_f_* integrations on heating compared to the equilibrium heat of fusion for fully crystalline polyethylene (Δ*H_f_^º^ =* 281 J/g)^5^.

Stress/strain experiments were performed at 10 mm/min using a Universal Test Machine (UTM2502) at room temperature. A standard test method, ASTM 638, was followed to measure the tensile properties of the polyethylene samples. Polymers were melt-pressed at 30 °C to 35 °C above their melting point to obtain the dog-bone-shaped tensile-test specimens. The test specimens showed around 25 mm gauge length, 2 mm width, and thickness of 0.4 mm. At least three specimens of each copolymer were tested.

The water contact angles on polymer films were measured with a Contact Angle Meter SL200B (Solon Tech. Co., Ltd.) using the dynamic sessile drop method. The reported values are the average of at least six measurements made at different positions of a film.

The ball-mill experiments were carried out in a 10 mL stainless steel jar with two 10 mm stainless steel balls on a Retsch MM400 mixer mill with a frequency of 30 Hz. The cryo-mill experiments were carried out in the same jar, ball, mixer mill, and frequency by deeping the jar in liquid nitrogen before milling.

# **Ethylene and CBE copolymerization**


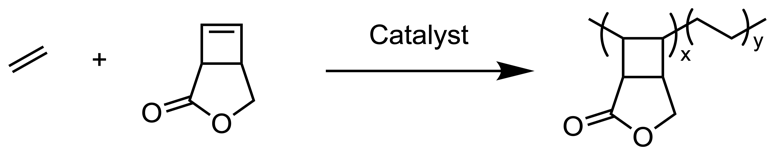


In a typical experiment, a 350 mL glass thick-walled pressure vessel was charged with chlorobenzene, a desired amount of CBE and a magnetic stir bar in the glovebox (A stainless steel Parr autoclave equipped with mechanical stirring was used for the runs at ethylene pressure of above 8 atm). The pressure vessel was connected to a high-pressure line and the solution was degassed. The vessel was warmed to the desire temperature using an oil bath and allowed to equilibrate for 5 min. The palladium catalyst (Pd1 or Pd2 or Pd3) in CH_2_Cl_2_ was injected into the polymerization system via syringe. With rapid stirring, the reactor was pressurized, maintained at a desired ethylene pressure, and stirred continuously for the desired period of time. After the pressure vessel was vented, the polymerization was quenched via the addition of methanol and the polymer was precipitated using excess methanol. After filtration, the copolymer was obtained and dried at 50 °C for 24 h under vacuum. The CBE incorporation (mol %) was calculated from ^1^H NMR analysis.

Table S1 Characterization of copolymers and commercial HDPE, LDPE, XLPE

| **Entry** | ***M_n_*^a^**  **(kDa)** | ***Đ*^a^** | ***F_CBE_*^b^**  **(%)** | ***T_d,95%_*^c^**  **(**°C**)** | ***T_m_*^d^**  **(**°C**)** | **Crystallinity**  **(%)^d^** | **Young’s modulus**  **(MPa)^e^** | **Stress at break**  **(MPa)^e^** | **Strain at break (%)^e^** |
| --- | --- | --- | --- | --- | --- | --- | --- | --- | --- |
| P1 | 82 | 1.9 | 12.6 | 443 | 58 | 12.2 | 116±26 | 53.8±2.1 | 600±40 |
| P2 | 62 | 2.0 | 5.7 | 448 | 106 | 34.2 | 156±21 | 41.9±1.1 | 890±60 |
| P3 | 26 | 1.9 | 12.4 | 442 | 69 | 16.1 | 117±2 | 33.1±1.1 | 680±40 |
| P4 | 90 | 2.6 | 7.5 | 451 | 77 | 27.2 | 141±17 | 45.3±2.4 | 790±40 |
| P5 | 65 | 2.6 | 2.0 | 453 | 118 | 48.5 | 194±33 | 20.9±1.9 | 820±60 |
| P6 | 190 | 2.6 | 0.5 | 455 | 128 | 53.9 | 259±10 | 53.3±1.2 | 1260±30 |
| HDPE | 57 | 6.8 | 0 | 426 | 132 | 64.1 | 320±18 | 45.4±3.5 | 1130±50 |
| LDPE | 36 | 5.1 | 0 | 425 | 111 | 53.8 | 240±20 | 20.4±2.7 | 840±40 |
| XLPE | -^f^ | - ^f^ | 0 | 469 | 127 | 40.7 | 617±65 | 20.4±2.6 | 437±30 |

^a)^ Determined by SEC at 150°C. ^b)^ Determined by ^1^H NMR at 120°C. ^c)^ Determined by TGA. ^d)^ Determined by DSC. ^e)^ Determined by tensile test. ^f)^ The polymer is not soluble to measure SEC.


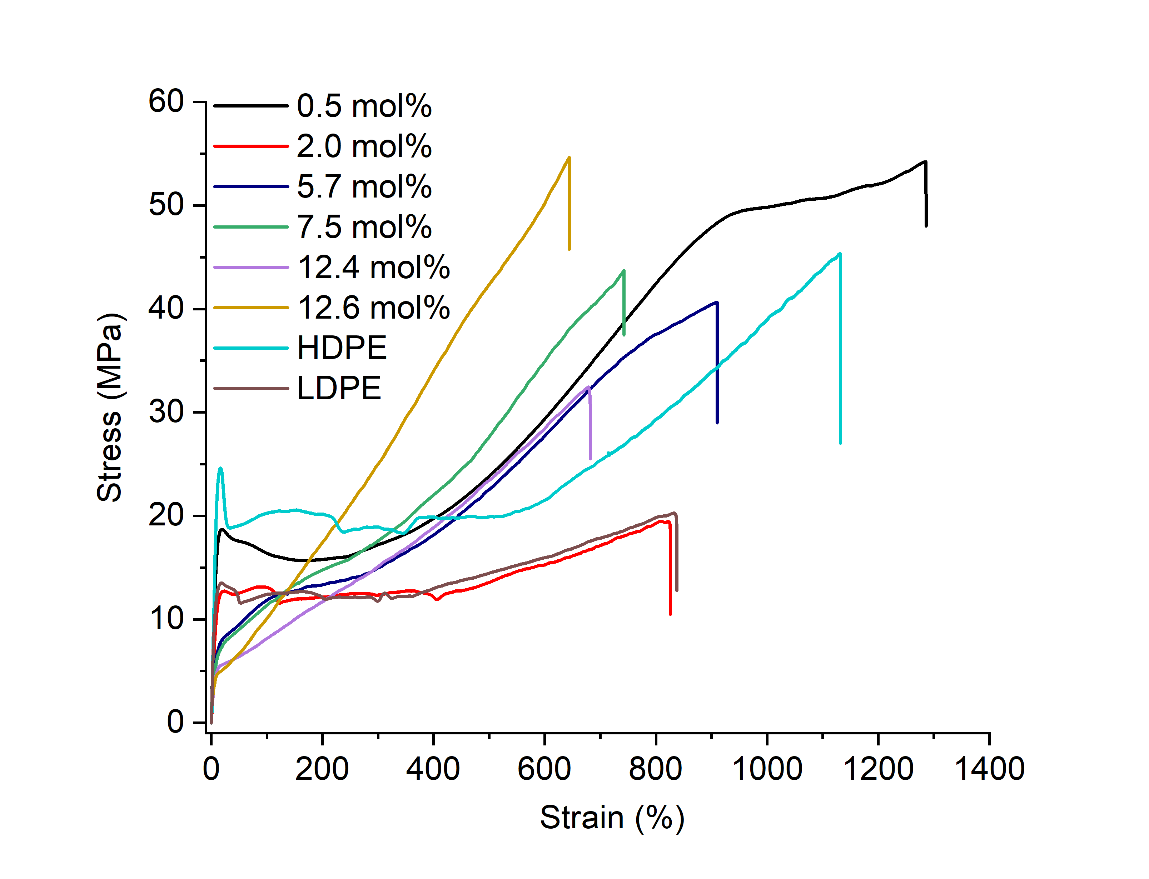


**Figure S1**. Strain-Stress curves of all synthesized copolymers, and commercial HDPE, LDPE.

For the Strain-Stress curve of commercial XLPE and recycled XLPE, please check the manuscript Figure 4.

# **Mechanochemical activation of copolymers**


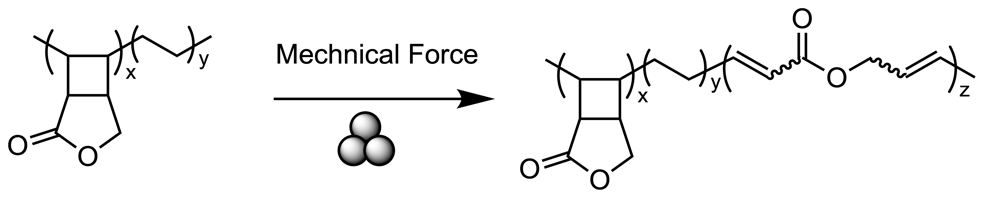


*Room temperature ball-milling (BM)*: 50 mg of the copolymer, 5 mg of butylated hydroxytoluene (BHT), and two stainless steel grinding balls (10 mm diameter) were placed into a 10 ml stainless steel grinding jar equipped with a screw-top lid. The sample was subjected to ball milling at a frequency of 30 Hz using a cycle of 5 minutes on followed by 5 minutes off, repeated until the desired total milling time was achieved. After completion, the jar was removed from the mixer mill and equilibrated to room temperature. Thereafter, the sample was taken and analyzed by SEC and ^1^H NMR.

*Cryo-milling (CM)*: 50 mg of the copolymer, 5 mg of butylated hydroxytoluene (BHT), and two stainless steel grinding balls (10 mm diameter) were placed into a 10 mL stainless steel grinding jar equipped with a screw-top lid. Prior to milling, the jar was submerged in liquid nitrogen for 20 minutes to ensure thorough cooling. The polymer was then subjected to mechanical grinding at a frequency of 30 Hz for 5 minutes. After each grinding cycle, the jar was re-submerged in liquid nitrogen for an additional 5 minutes. This alternating process between cryogenic cooling and mechanical grinding was repeated until the desired total milling time was reached. After completion, the jar was removed from the mixer mill and allowed to warm to room temperature. The resulting polymer sample was then collected and analyzed by SEC and ^1^H NMR.

Table S2 Optimization of milling conditions (with and without BHT) using P1

| **Milling Time** | | 20min | 40min | 60min | 90min | 120min |
| --- | --- | --- | --- | --- | --- | --- |
| **Ring Opening Yield** | **BM** | 6% | 18% | 31%  (42% with BHT) | insoluble | insoluble |
|  | **CM** | 17% | 34% | 38%  (57% with BHT) | 20%  (55% with BHT) | insoluble |

BM: ball-mill grinding, CM: cryo-mill grinding, Ring opening yield (%) = z/x×100% based on ^1^H NMR integration.

Table S3 Optimization of milling conditions (with BHT) using P6

| **Milling Frequency** | 10Hz | 20Hz | 30Hz |
| --- | --- | --- | --- |
| **Ring Opening Yield**  **By BM** | 0% | 15% | 50% |

# **Degradation of mechanical activated copolymers**

40 mg of mechanical activated polymer, 1 mg of triazabicyclodecene (TBD), 1 mL of ethanol (EtOH), and 4 mL of xylene were added to a 25 mL pressure reaction tube. The mixture was bubbled with Argon for 15 minutes, then reaction tube was sealed and heated at 120 °C for 12 hours. Thereafter, the reaction mixture was precipitated into 20 mL of methanol. The resulting precipitate was collected by filtration and dried under vacuum at 50 °C for 24 hours to obtain the degraded fractions.

Table S4 Mechanical activation and degradation of copolymers

| **Entry** | ***M_n_*** ^a^  **(kDa)** | ***Đ*** ^a^ | ***F_CBE_***  **(%)**^b^ | **Ring opening yield (%) BM/CM (1h)** ^b^ | ***M_n_***  **(kDa) after**  **BM/CM** ^a^ | ***M_n_* (kDa) after**  **ethanolysis**  **BM/CM** ^a^ | ***M_n, theo._* (kDa) after**  **ethanolysis BM/CM** ^b^ |
| --- | --- | --- | --- | --- | --- | --- | --- |
| P1 | 82 | 1.9 | 12.6 | 42/57 | 49/28 | <1/<1^c^ | 0.5/0.4 |
| P2 | 62 | 2.0 | 5.7 | 23/43 | 36/30 | 1.6/1.3 | 2.0/1.1 |
| P3 | 26 | 1.9 | 12.4 | 6/38 | 16/23 | <1/<1 ^c^ | 4.5/0.6 |
| P4 | 90 | 2.6 | 7.5 | 17/52 | 51/51 | <1/<1 ^c^ | 2.0/0.7 |
| P5 | 65 | 2.6 | 2.0 | 31/35 | 31/33 | 8.4/9.8 | 4.5/4.1 |
| P6 | 190 | 2.6 | 0.5 | 50/49 | 51/57 | 21/22 | 12.4/12.7 |
| HDPE | 57 | 6.8 | 0 | - | 26/37 | 21/33 | - |
| LDPE | 36 | 5.1 | 0 | - | 22/22 | 24/25 | - |

^a)^ Determined by SEC at 150°C. ^b)^ Determined by ^1^H NMR at 120°C. ^c)^ The molecular weight is out range of the calibration in the SEC.


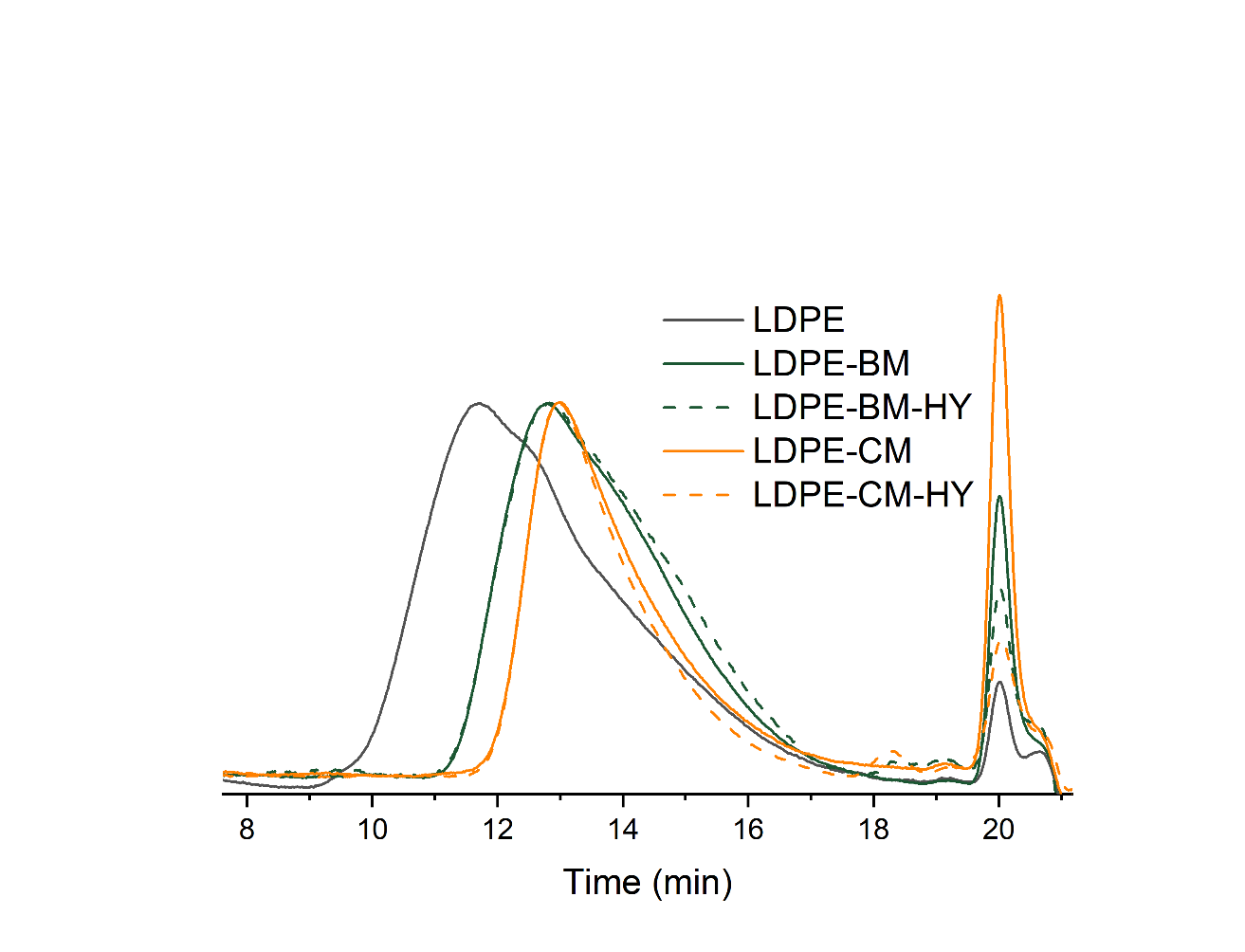


**Figure S2**. SEC of mechanical activated and degraded of commercial LDPE


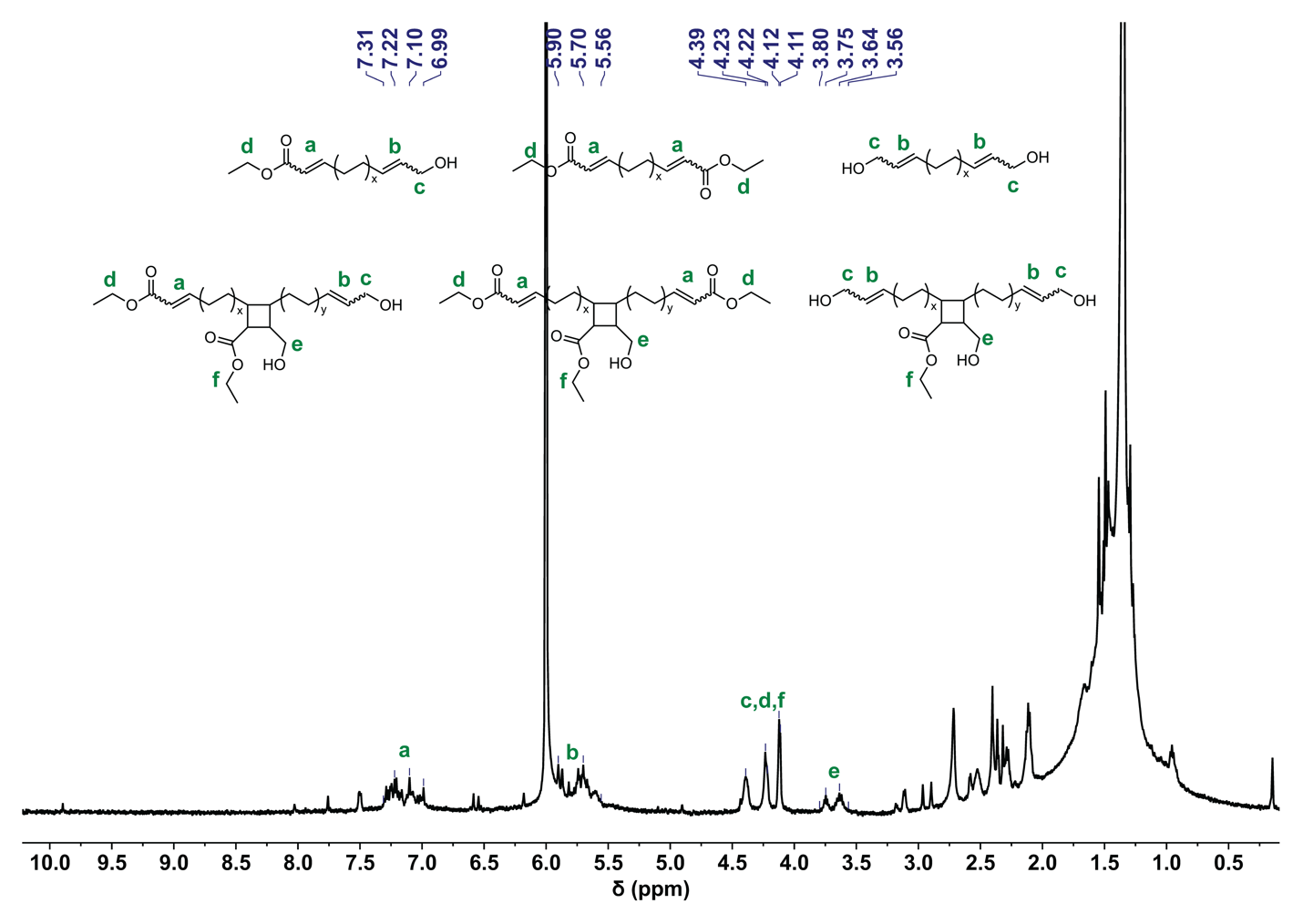


**Figure S3**. ^1^H NMR of degraded fractions of P1 by cryo-mill and ethanolysis (C_2_D_2_Cl_4_, 500 MHz, 120°C).

# ****

**Figure S4**. ^1^H NMR of degraded fractions of P4 under cryo-mill-hydrogenation-ethanolysis and ball-mill-hydrogenation-ethanolysis (C_2_D_2_Cl_4_, 500 MHz, 120°C).


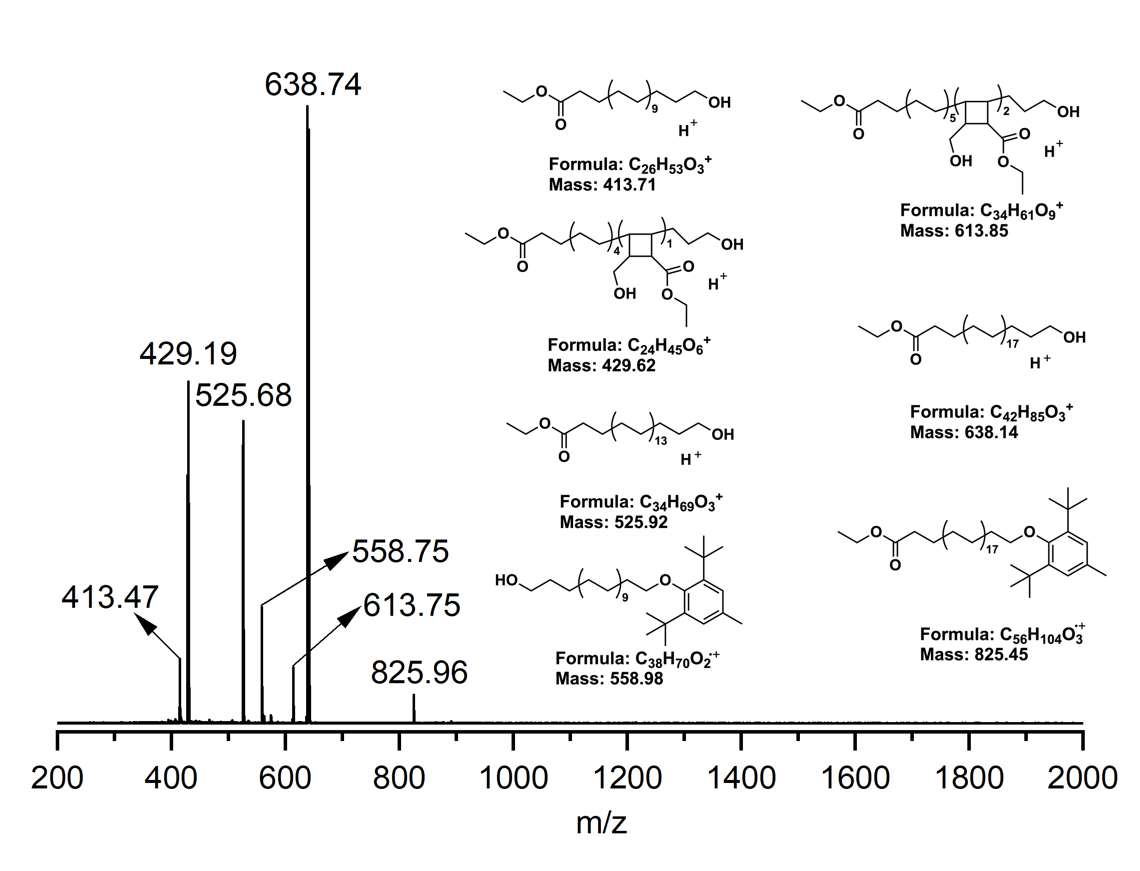


**Figure S5**. MALDI-ToF and possible chemical structures of degraded fractions of P4 after ball-mill-hydrogenation-ethanolysis.


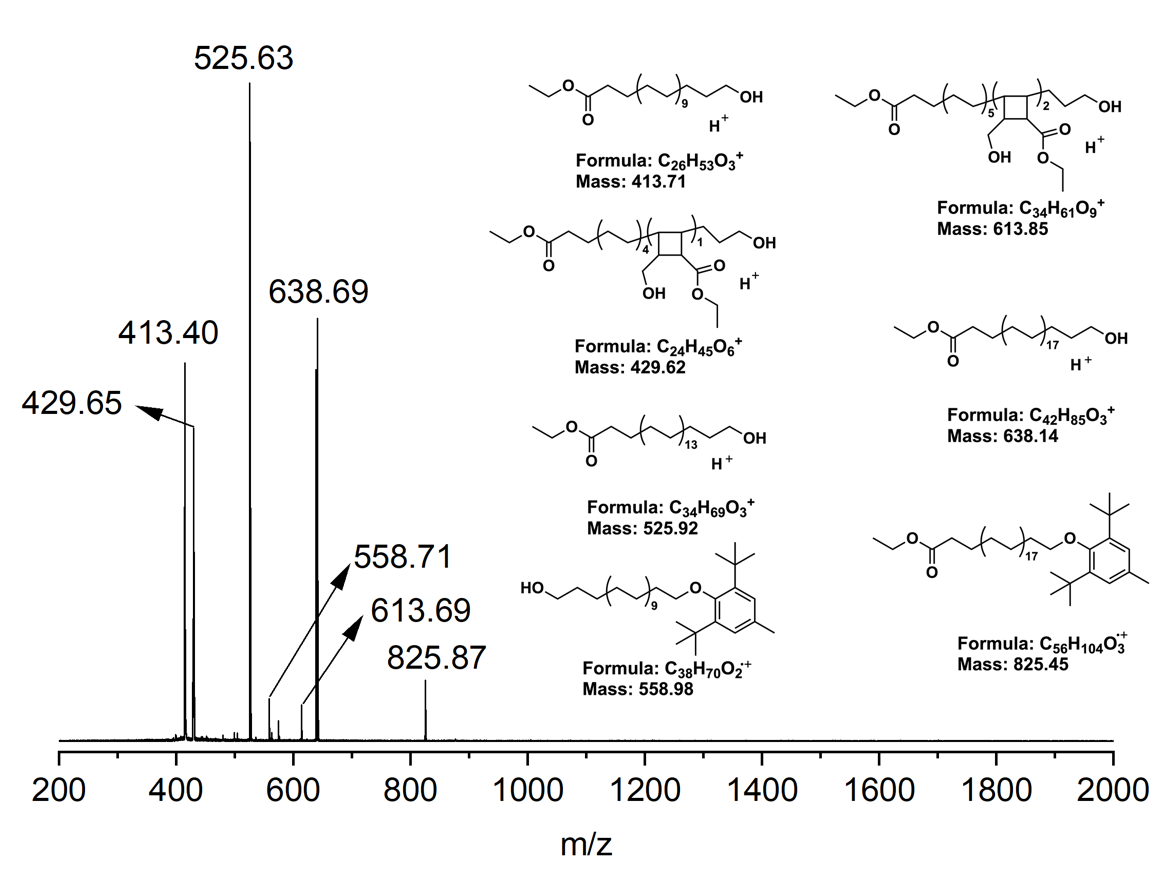


**Figure S6**. MALDI-ToF and possible chemical structures of degraded fractions of P4 after cryo-mill-hydrogenation-ethanolysis.

# **Repolymerization of the degraded fractions**

Mechanically degraded polymer (40 mg), tripropylamine (TPA, 0.5 mL), *p*-toluenesulfonyl hydrazide (PTSH, 100 mg), and xylene (5 mL) were added to a 25 mL pressure reaction tube. The mixture was purged with argon for 5 minutes. Then the reaction tube was sealed, and heated at 120 °C for 12 hours. Thereafter, the reaction mixture was precipitated into 20 mL of methanol. The resulting solid was collected by filtration and dried under vacuum at 50 °C for 24 hours to yield the hydrogenated polymer.

The hydrogenated fractions and titanium butoxide (Ti(O*^n^*Bu)_4_, 0.5 mg) were placed in a 10 mL Schlenk flask. The flask was purged with argon three times, then heated to 150 °C under an argon atmosphere. After 1 hour, the mixture was placed on a stainless-steel mold (length = 80.0 mm, width = 80.0 mm, thickness = 0.5 mm) and pressed between two polytetrafluoroethylene sheets under 2 kN pressure at 180 °C for 5 hours, along with high vacuum. The polymer was then cooled to room temperature. The resulting crosslinked polymer was characterized by TGA, DSC, and tensile testing.

# **^1^H NMR of copolymers**

**Figure S7**. ^1^H NMR of P1 (C_2_D_2_Cl_4_, 500 MHz, 120°C). CBE incorporation ratio: 1/(1+27.8/4)×100% = 12.6%

**Figure S8**. ^1^H NMR of P2 (C_2_D_2_Cl_4_, 500 MHz, 120°C). CBE incorporation ratio: 1/(1+65.81/4)×100% = 5.7%

**Figure S9**. ^1^H NMR of P3 (C_2_D_2_Cl_4_, 500 MHz, 120°C). CBE incorporation ratio: 1/(1+28.15/4)×100% = 12.4%

**Figure S10**. ^1^H NMR of P4 (C_2_D_2_Cl_4_, 500 MHz, 120°C). CBE incorporation ratio: 1/(1+49.68/4)×100% = 7.5%

**Figure S11**. ^1^H NMR of P5 (C_2_D_2_Cl_4_, 500 MHz, 120°C). CBE incorporation ratio: 1/(1+191/4)×100% = 2.0%

**Figure S12**. ^1^H NMR of P6 (C_2_D_2_Cl_4_, 500 MHz, 120°C). CBE incorporation ratio: 1/(1+840.06/4)×100% = 0.5%

# **^1^H NMR of mechanical activated copolymers**

**Figure S13**. ^1^H NMR of P1 after mechanical activation (C_2_D_2_Cl_4_, 500 MHz, 120°C). CBE ring opening yield: CM 2.63/(2.63+2)×100% = 57%, BM 1.46/(1.46+2)×100% = 42%.

**Figure S14**. ^1^H NMR of P2 after mechanical activation (C_2_D_2_Cl_4_, 500 MHz, 120°C). CBE ring opening yield: CM 1.49/(1.49+2)×100% = 43%, BM 0.6/(0.6+2)×100% = 23%.

**Figure S15**. ^1^H NMR of P3 after mechanical activation (C_2_D_2_Cl_4_, 500 MHz, 120°C). CBE ring opening yield: CM 1.22/(1.22+2)×100% = 38%, BM 0.12/(0.12+2)×100% = 6%.

**Figure S16**. ^1^H NMR of P4 after mechanical activation (C_2_D_2_Cl_4_, 500 MHz, 120°C). CBE ring opening yield: CM 2.18/(2.18+2)×100% = 52%, BM 0.42/(0.42+2)×100% = 17%.

**Figure S17**. ^1^H NMR of P5 after mechanical activation (C_2_D_2_Cl_4_, 500 MHz, 120°C). CBE ring opening yield: CM 1.07/(1.07+2)×100% = 35%, BM 0.9/(0.9+2)×100% = 31%.

**Figure S18**. ^1^H NMR of P6 after mechanical activation (C_2_D_2_Cl_4_, 500 MHz, 120°C). CBE ring opening yield: CM 1.91/(1.91+2)×100% = 49%, BM 1.93/(1.93+2)×100% = 50%.

# **SEC of copolymers**

**Figure S19**. SEC of P1.


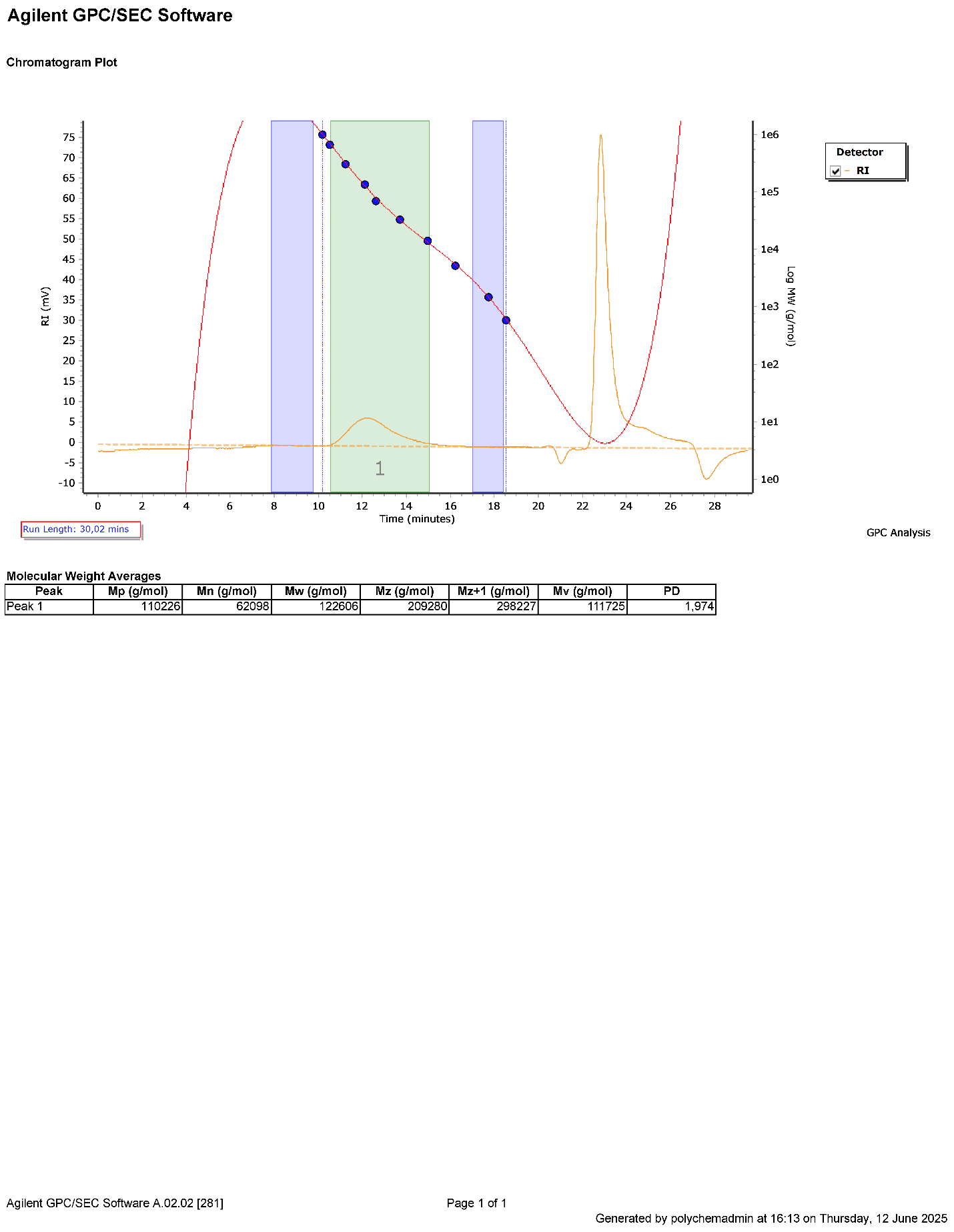


**Figure S20**. SEC of P2.


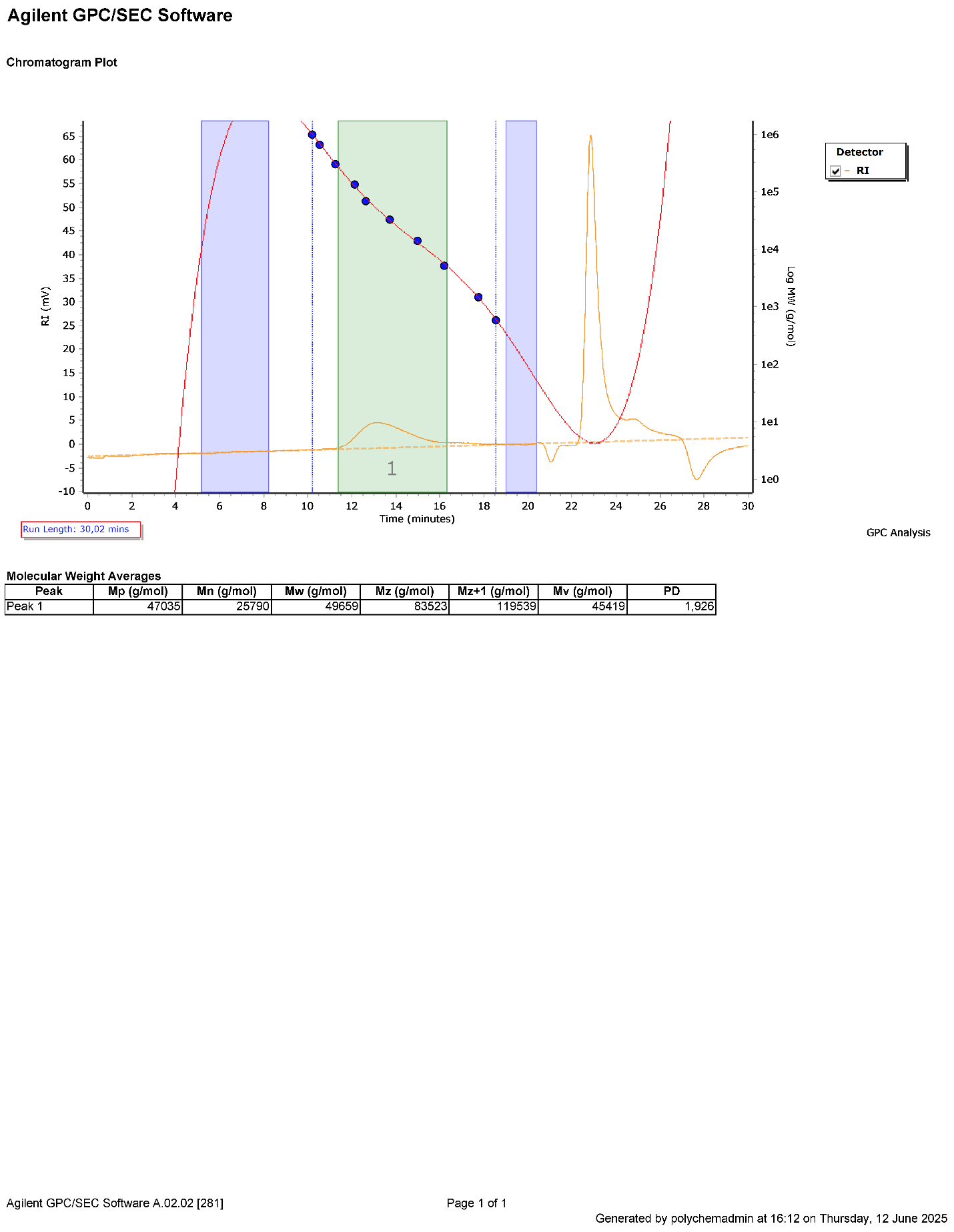


**Figure S21**. SEC of P3.


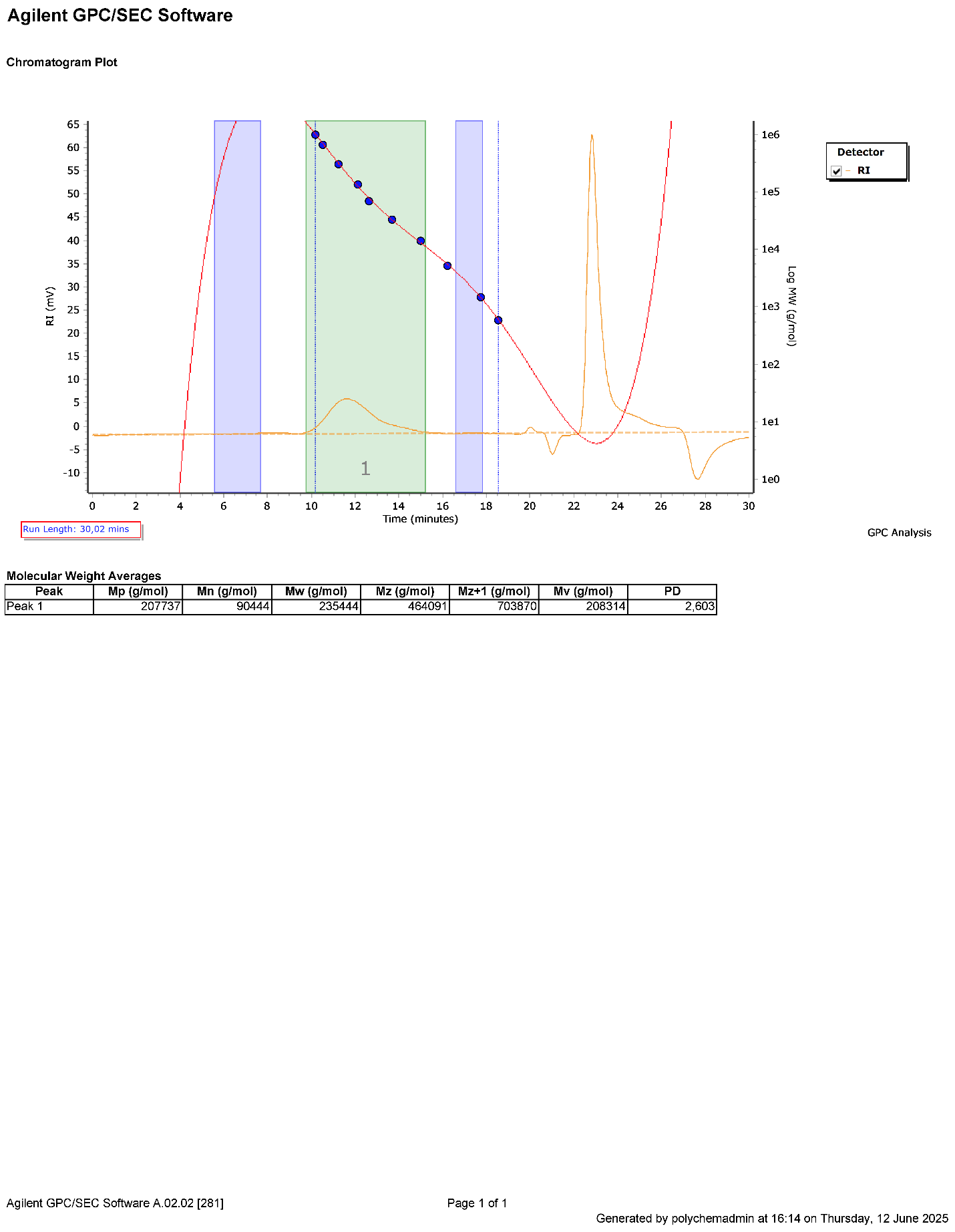


**Figure S22**. SEC of P4.


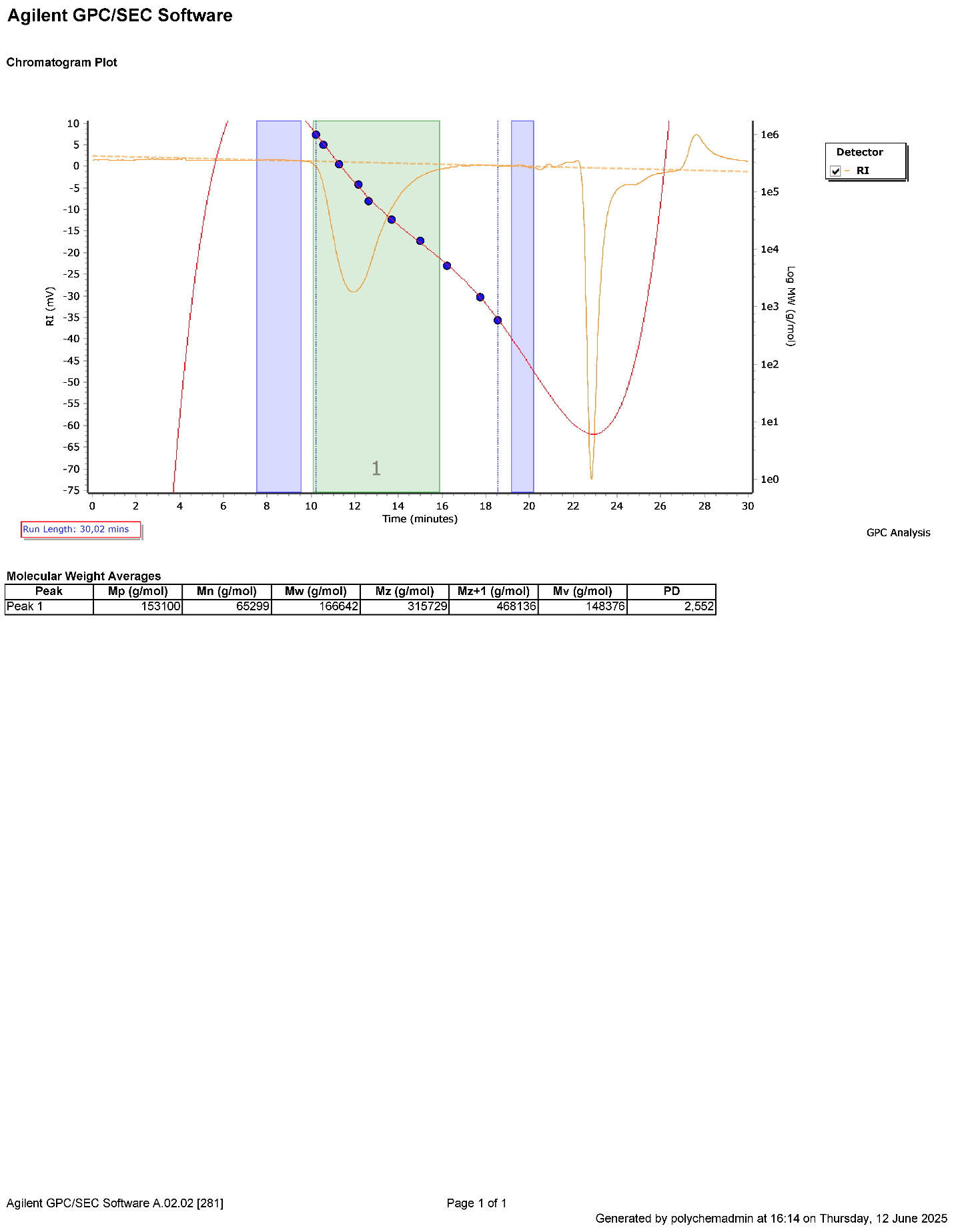


**Figure S23**. SEC of P5.


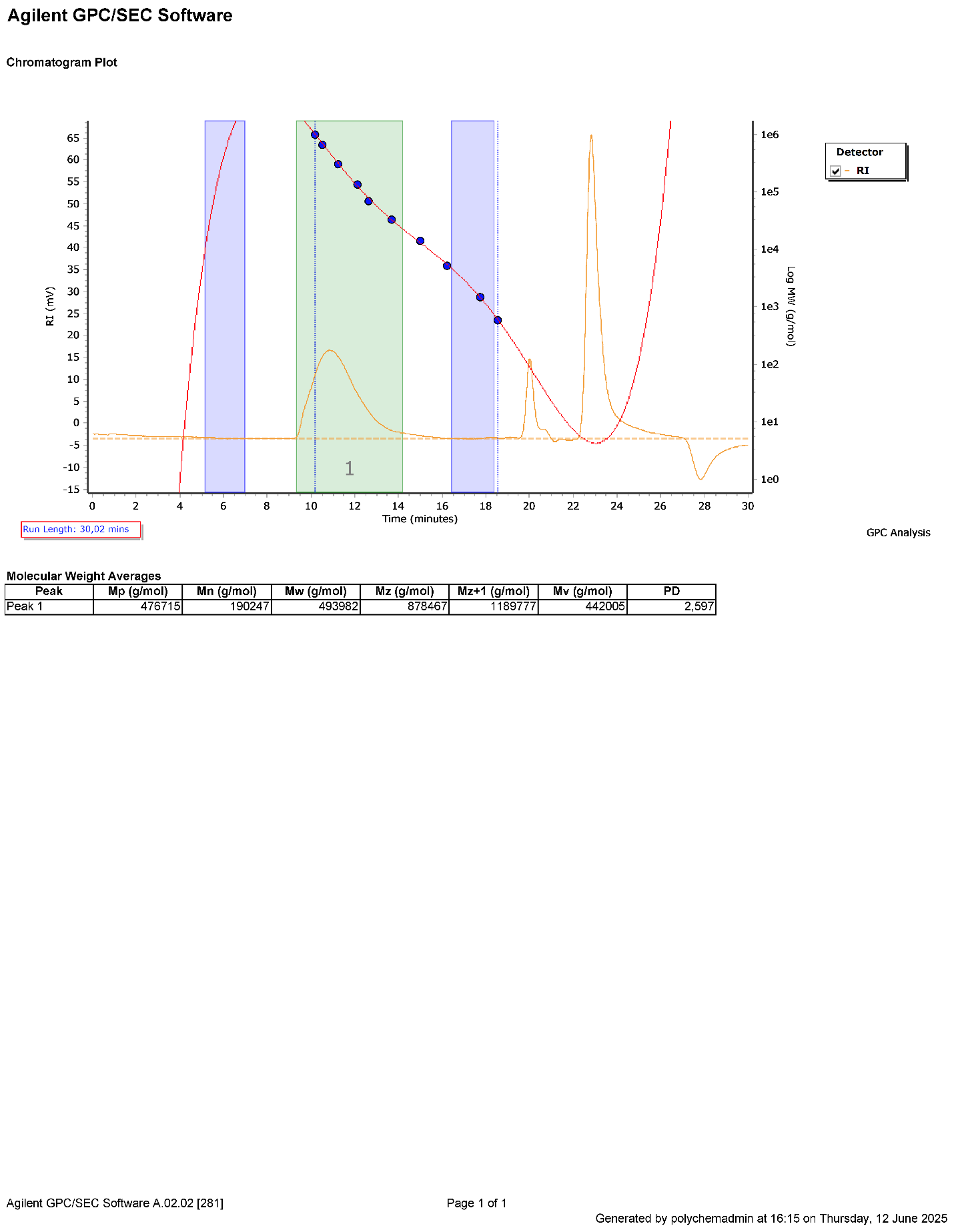


**Figure S24**. SEC of P6.


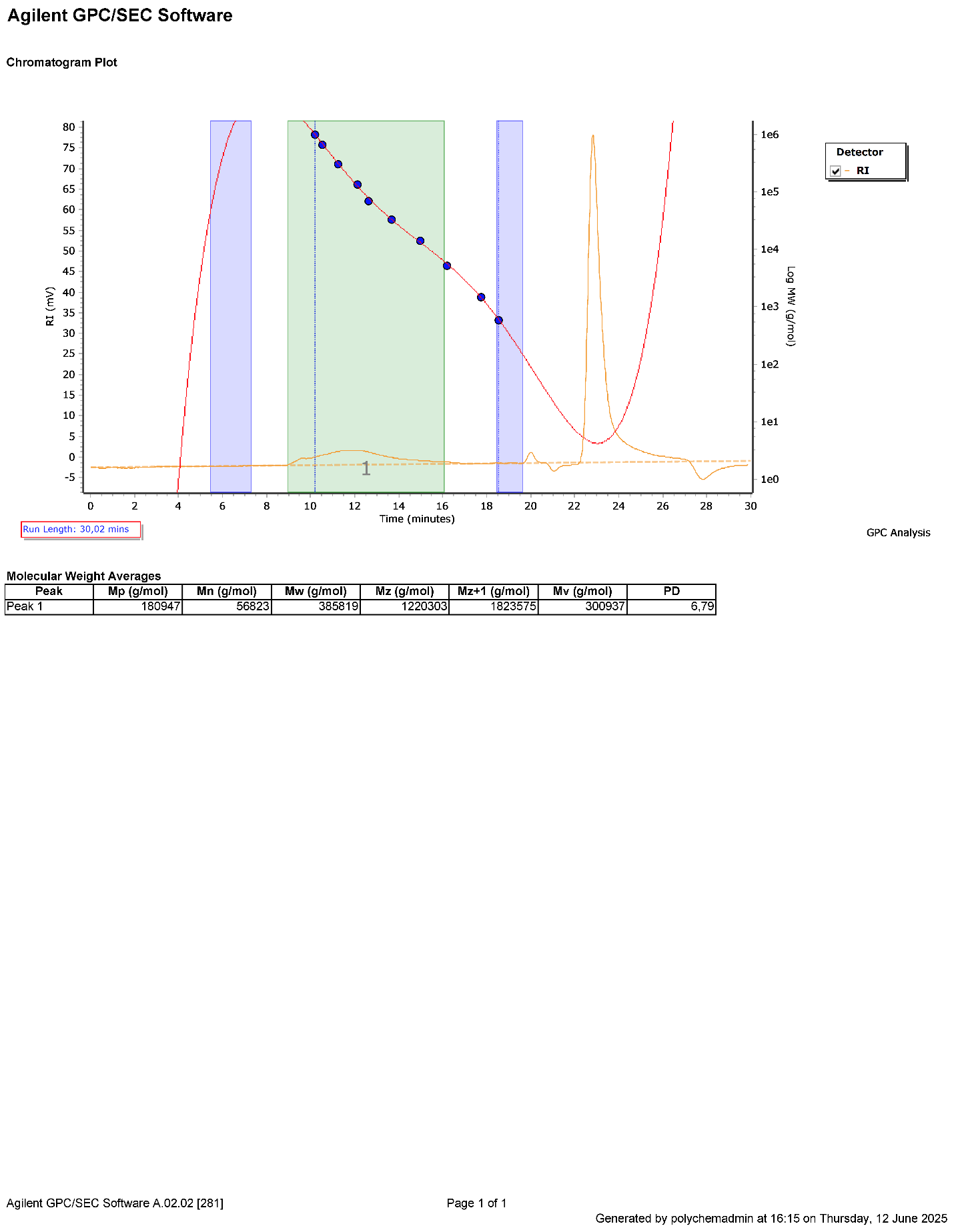


**Figure S25**. SEC of commercial HDPE.


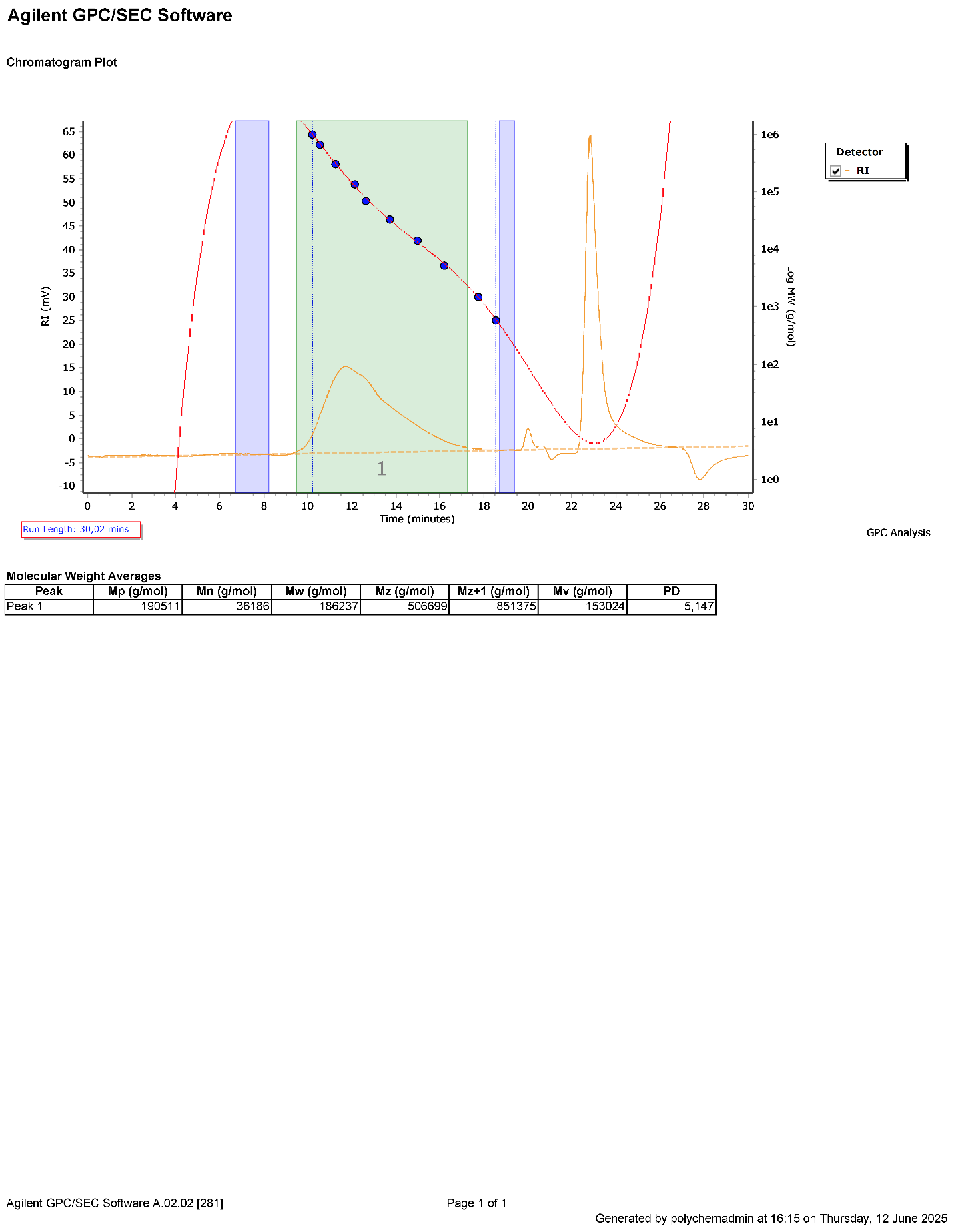


**Figure S26**. SEC of commercial LDPE.

# **SEC of mechanical activated polymers**

**Figure S27.** SEC of mechanical activated P1 by ball-mill.


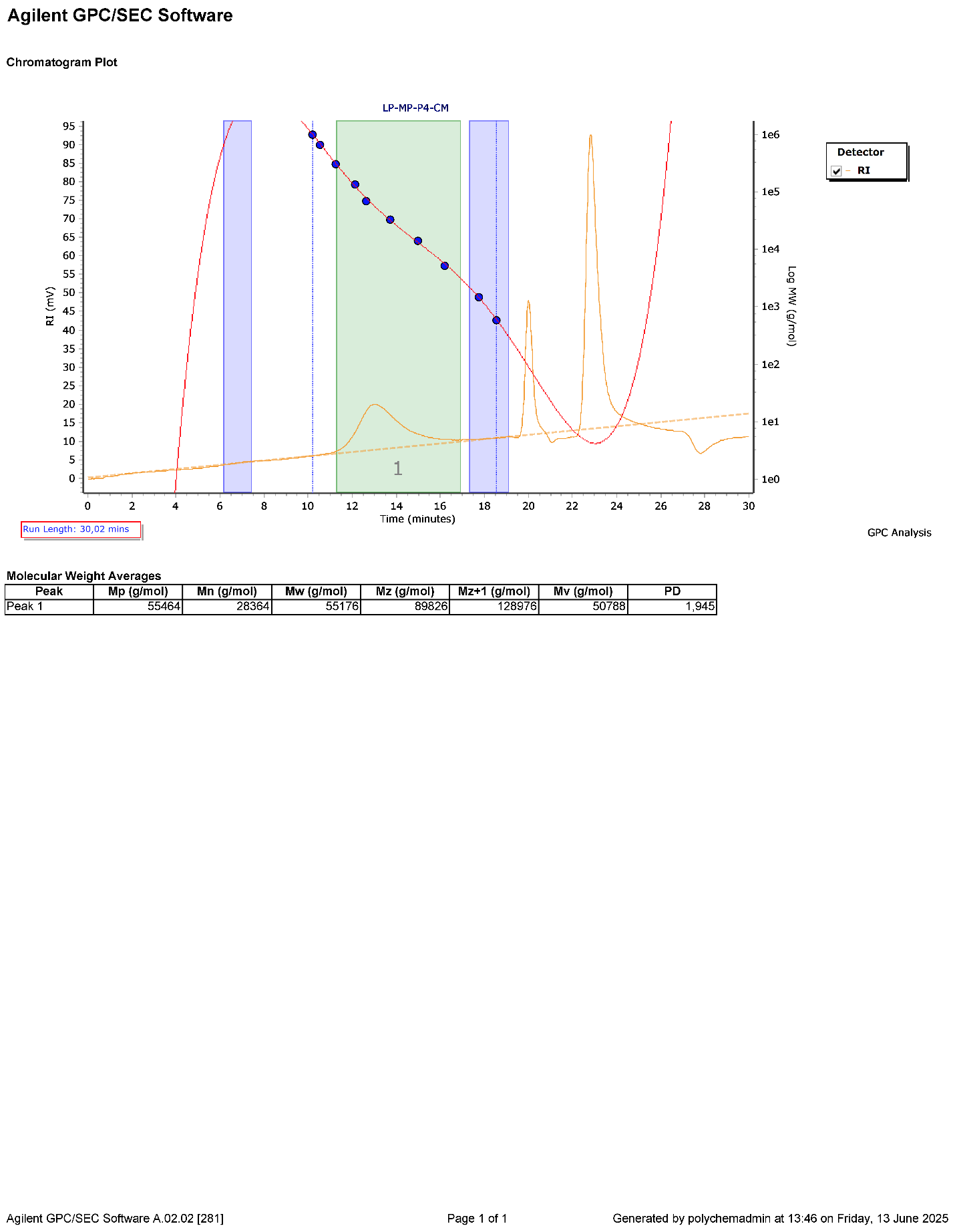


**Figure S28**. SEC of mechanical activated P1 by cryo-mill.


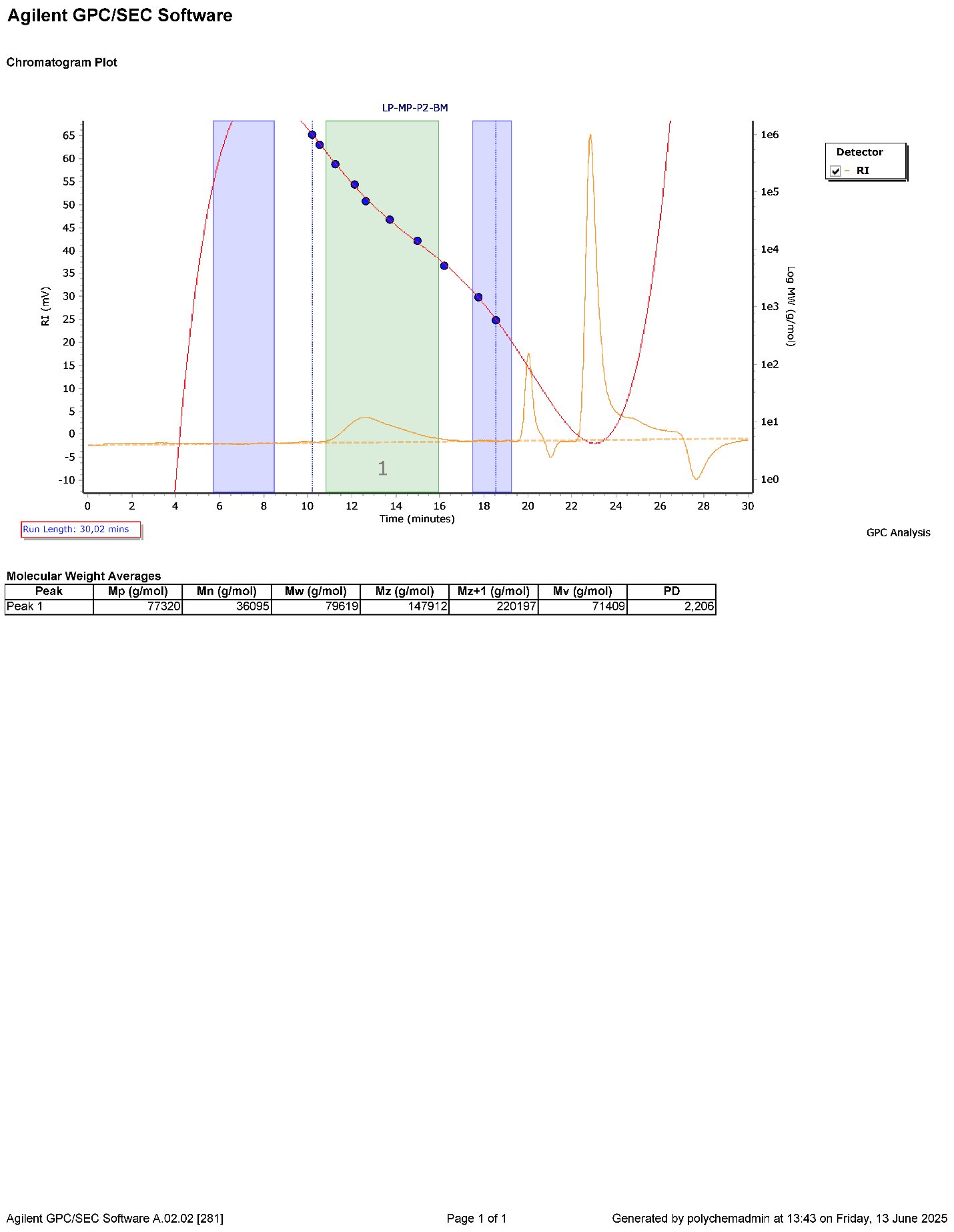


**Figure S29**. SEC of mechanical activated P2 by ball-mill.


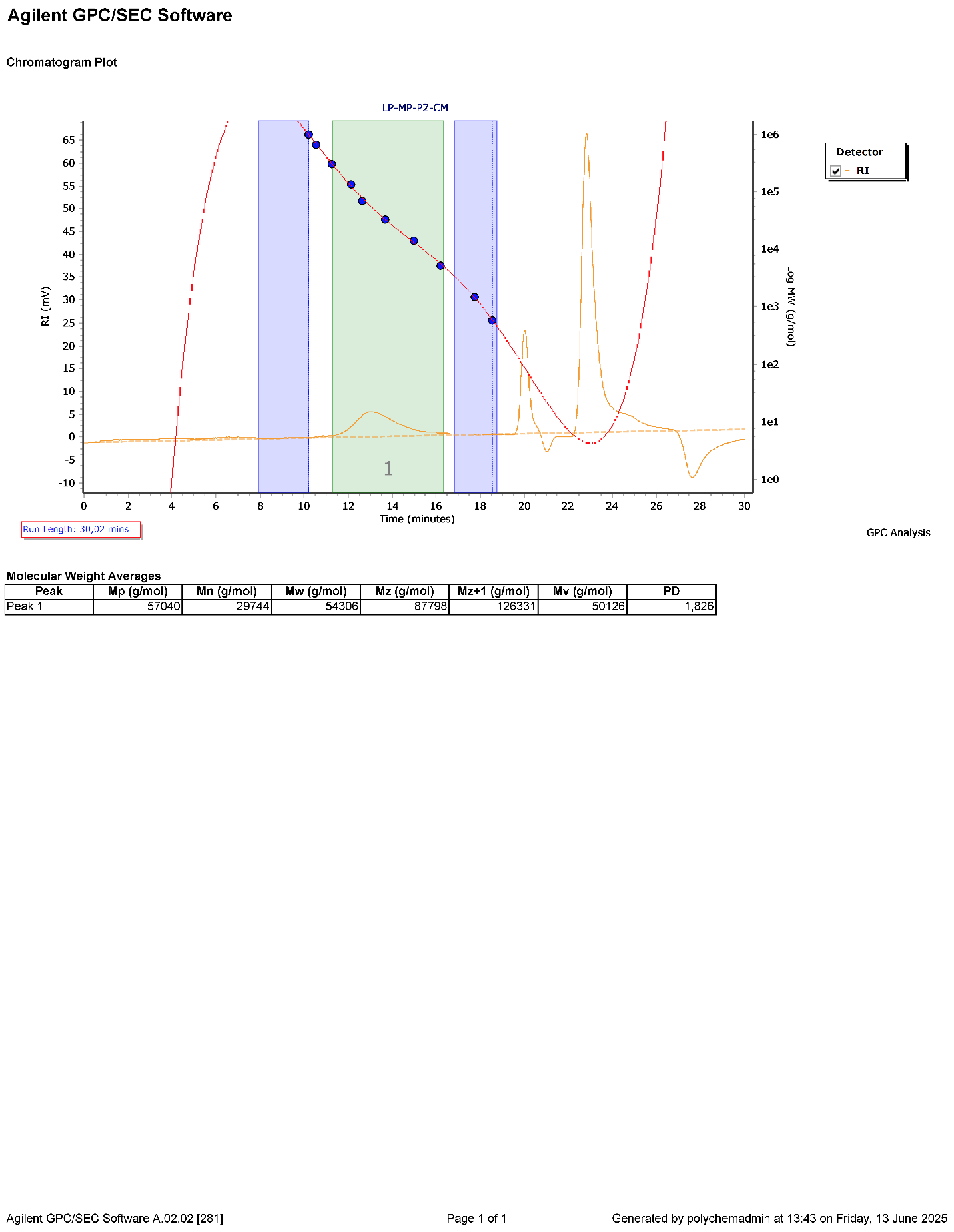


**Figure S30**. SEC of mechanical activated P2 by cryo-mill.


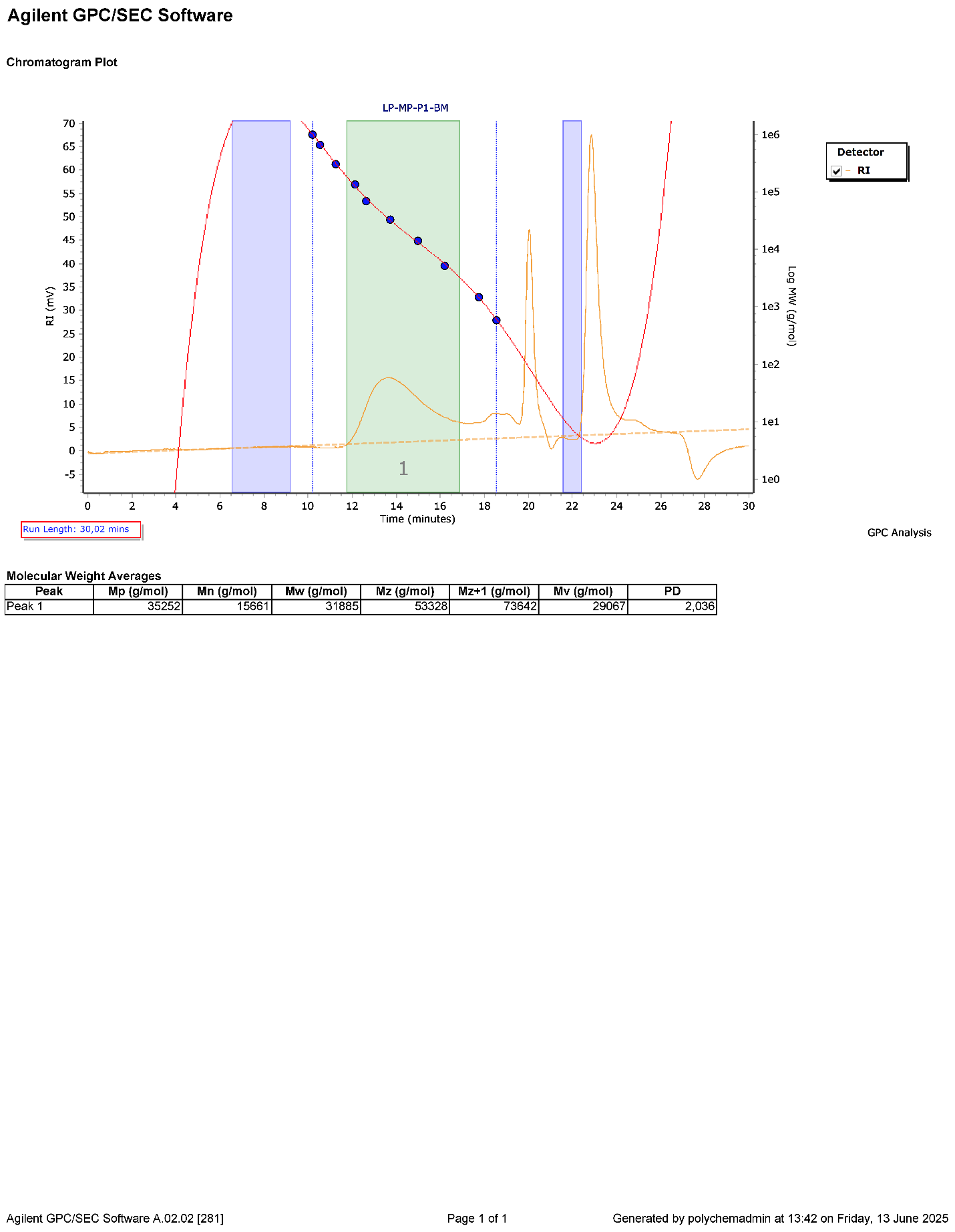


**Figure S31.** SEC of mechanical activated P3 by ball-mill.


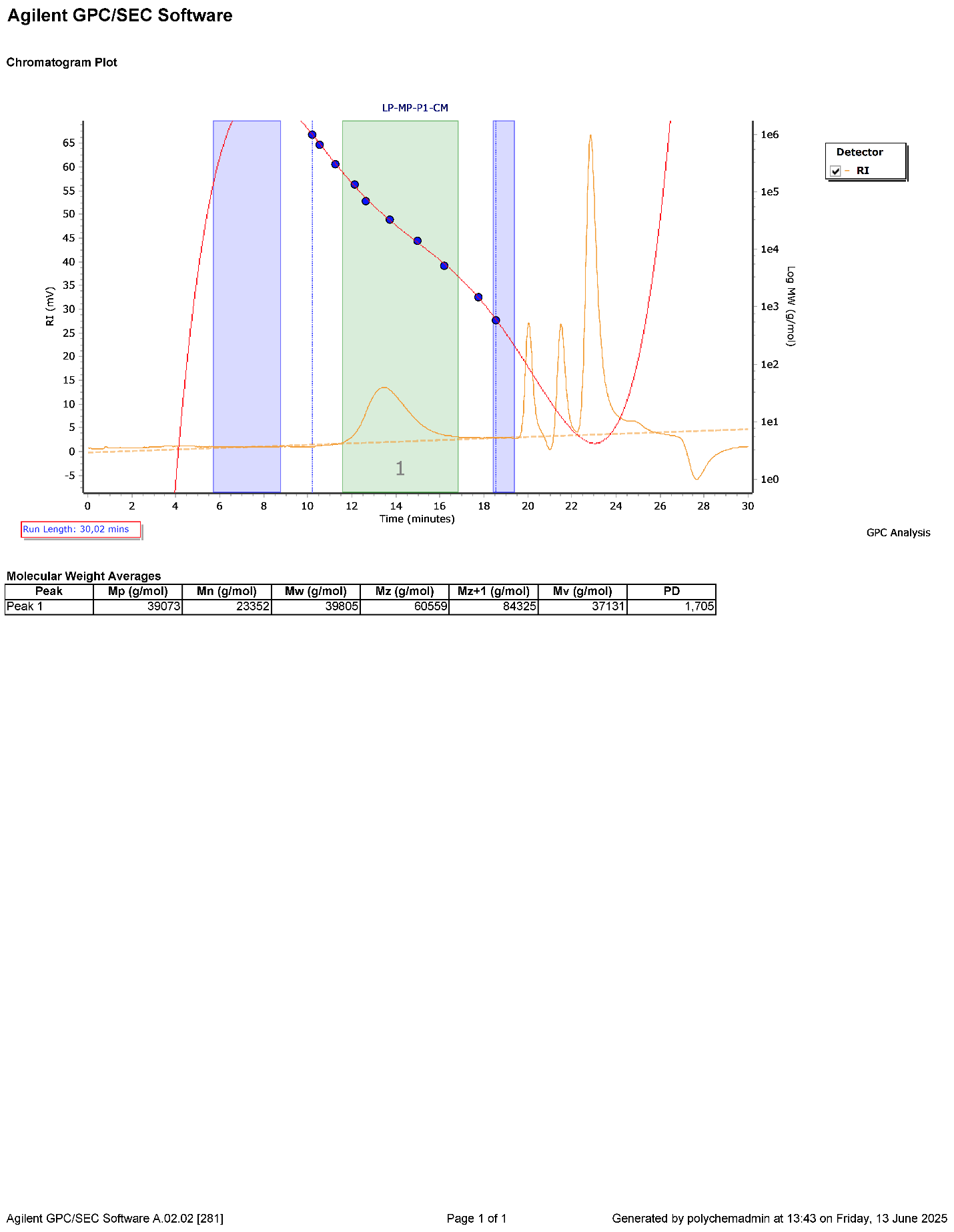


**Figure 32.** SEC of mechanical activated P3 by cryo-mill


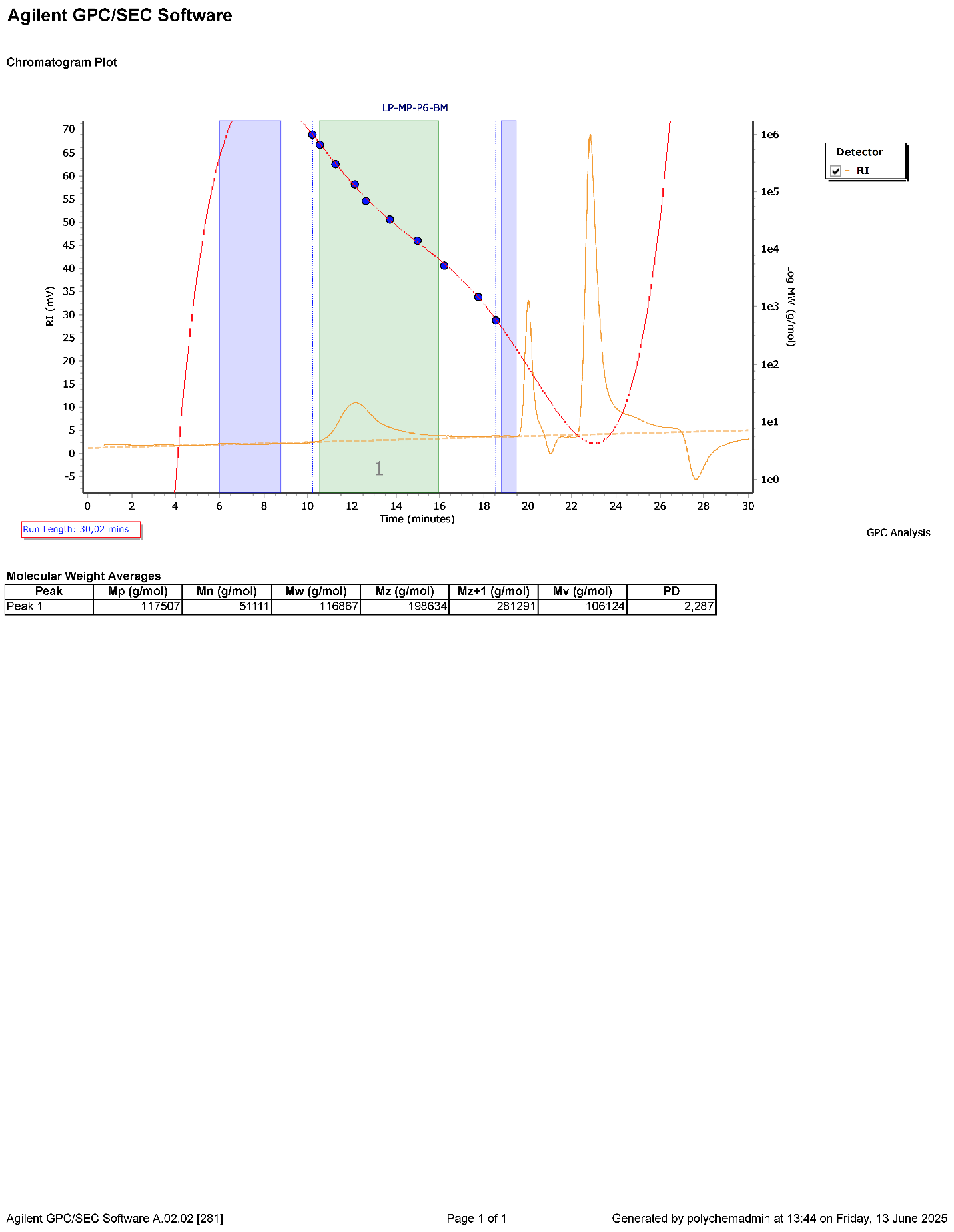


**Figure 33.** SEC of mechanical activated P4 by ball-mill.


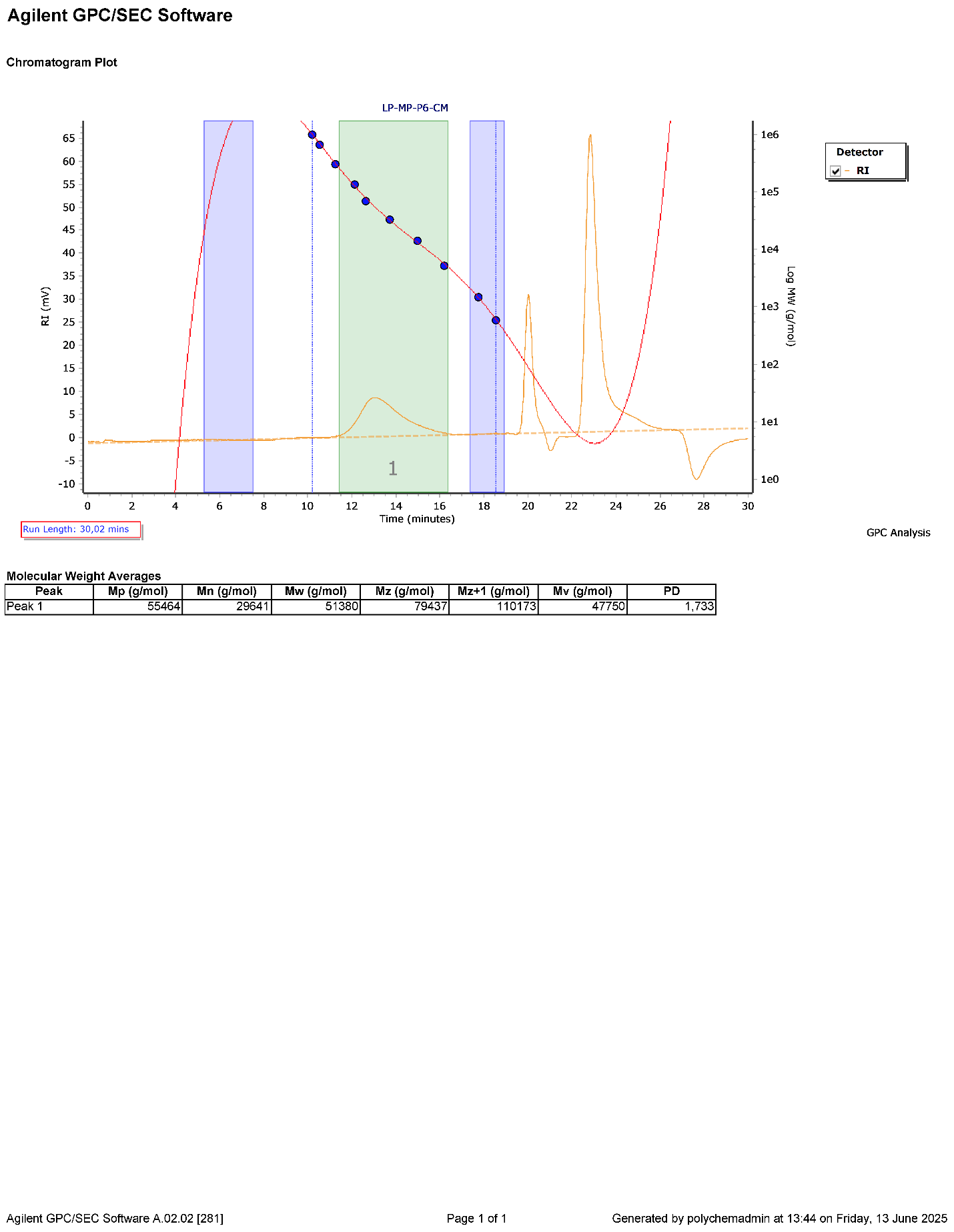


**Figure S34**. SEC of mechanical activated P4 by cryo-mill.


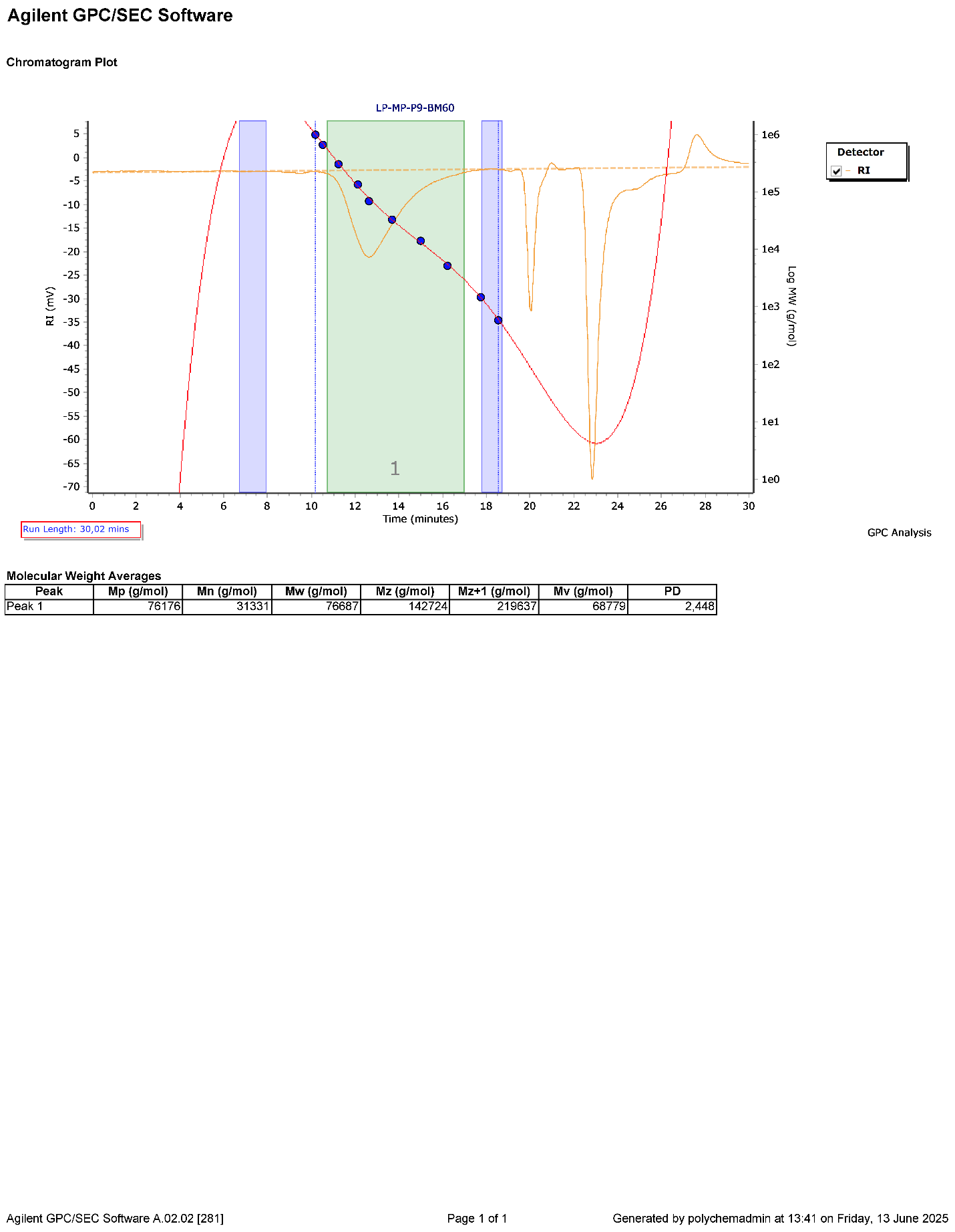


**Figure S35.** SEC of mechanical activated P5 by ball-mill.


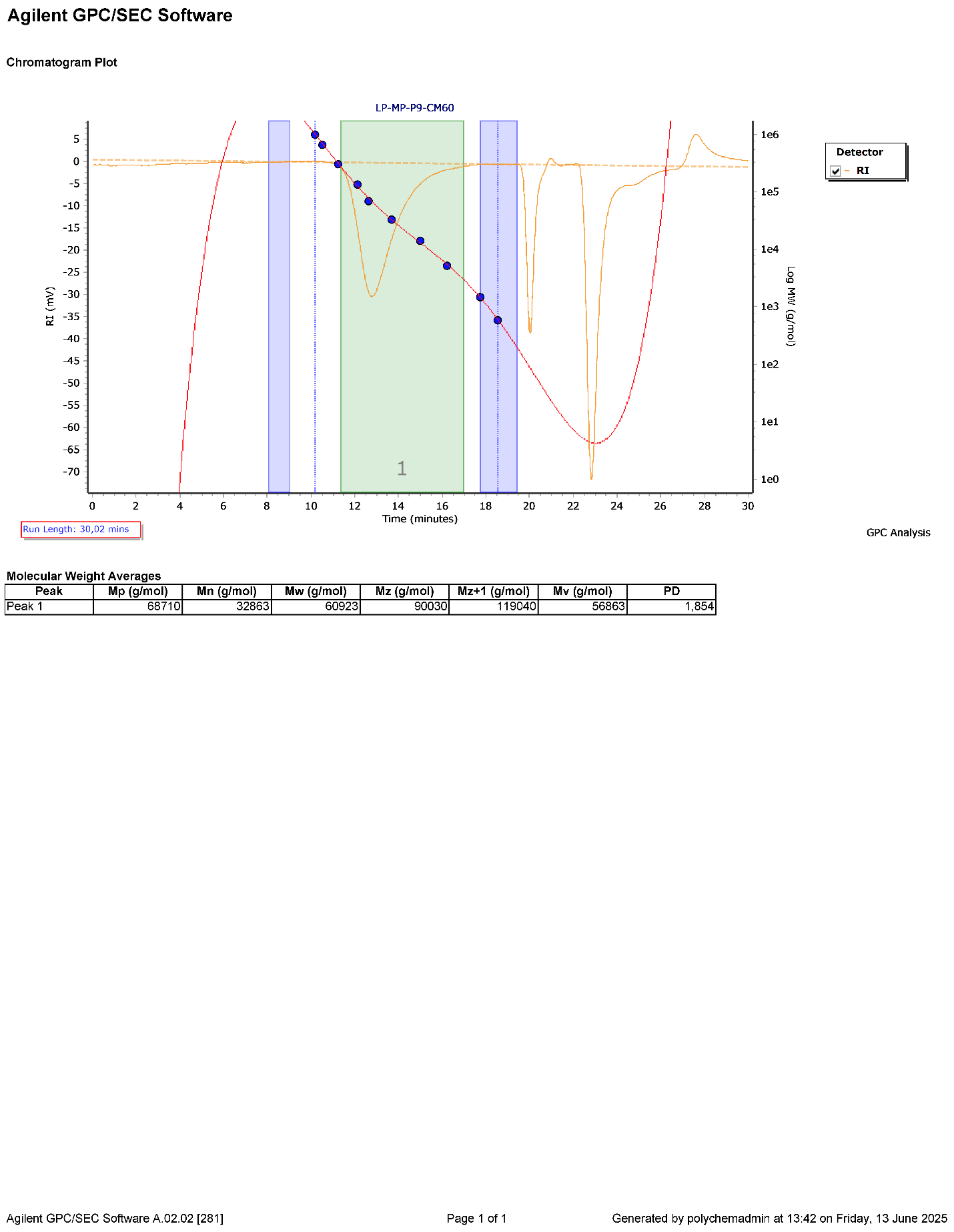


**Figure S36.** SEC of mechanical activated P5 by cryo-mill.


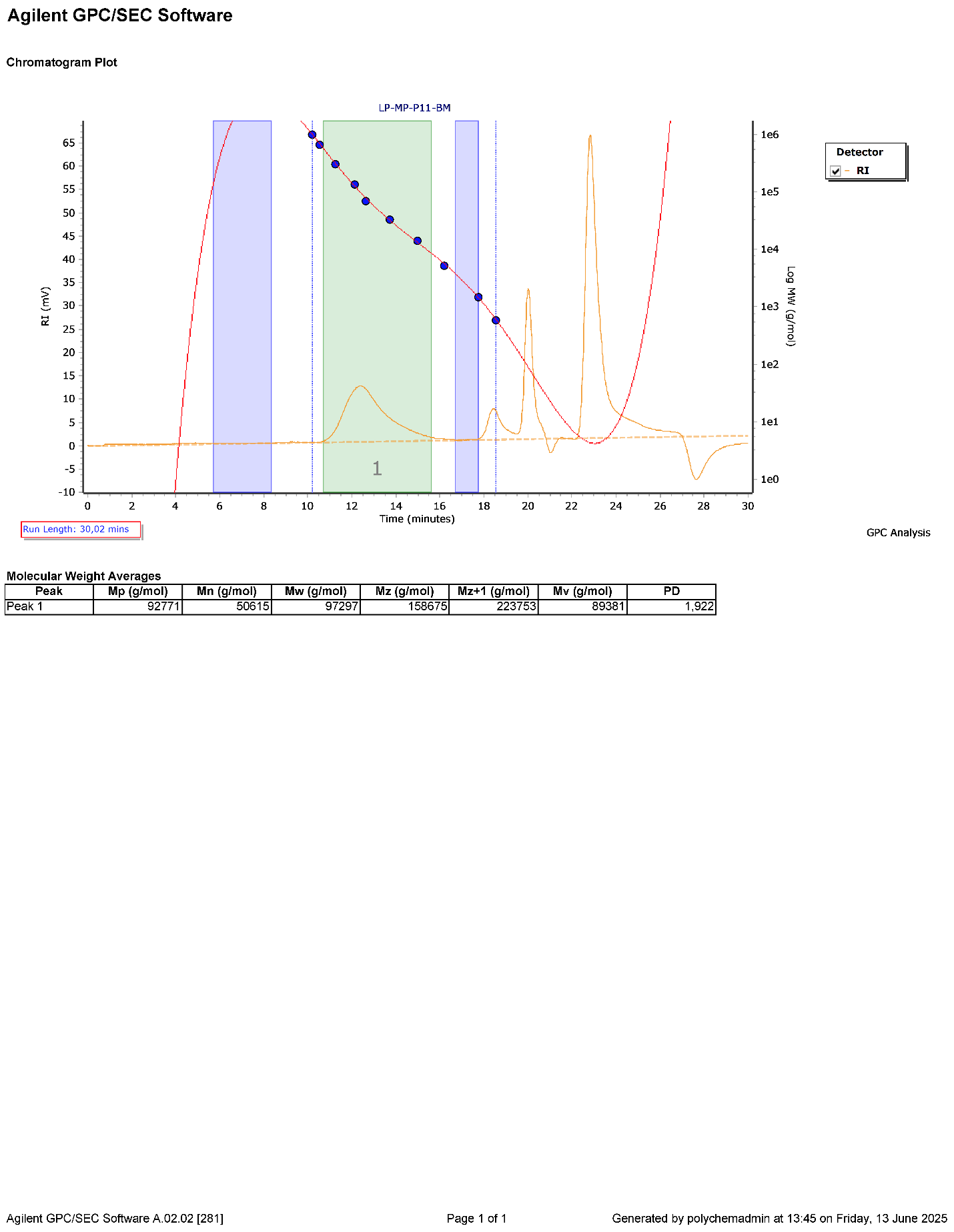


**Figure S37.** SEC of mechanical activated P6 by ball-mill.


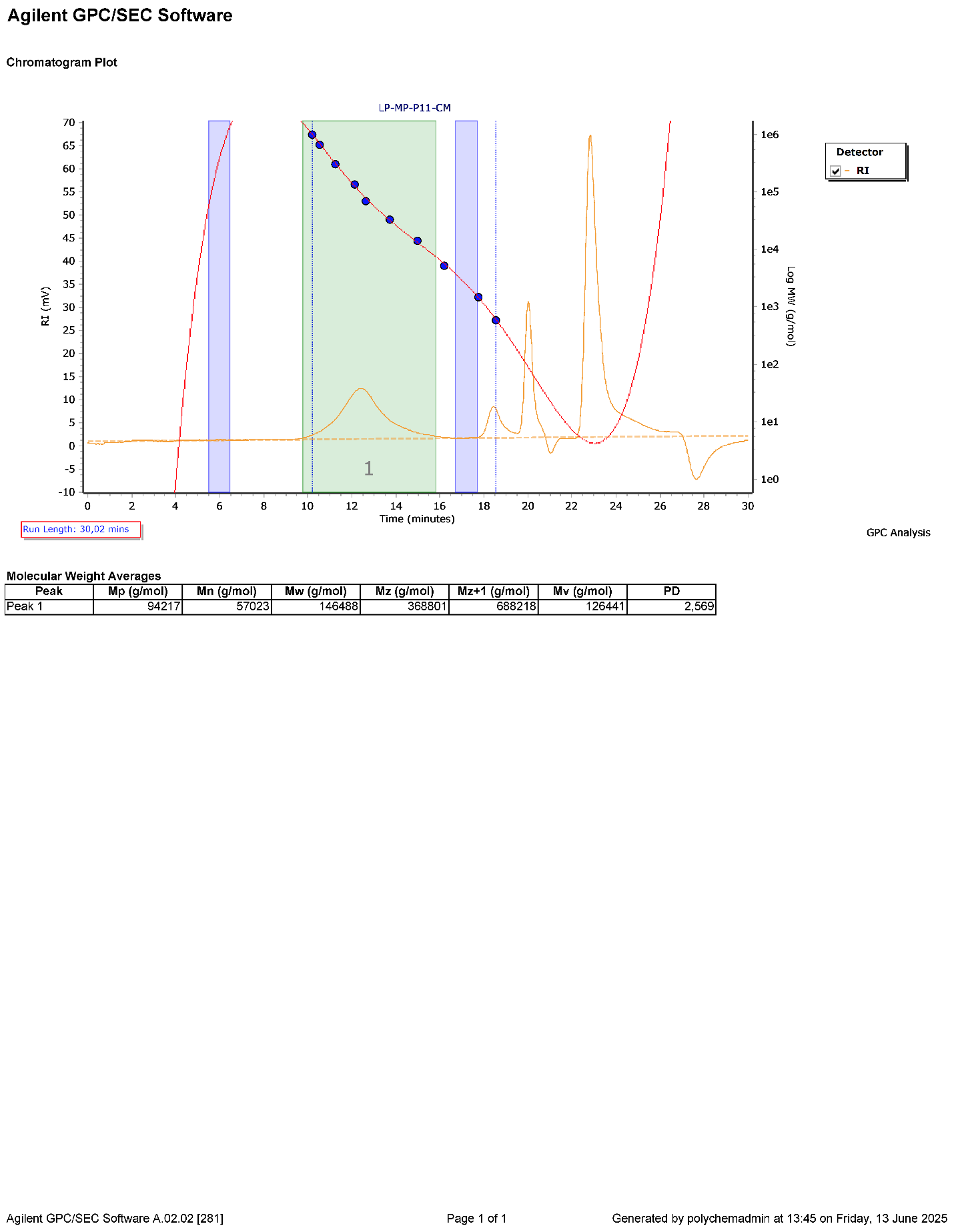


**Figure 38.** SEC of mechanical activated P6 by cryo-mill.


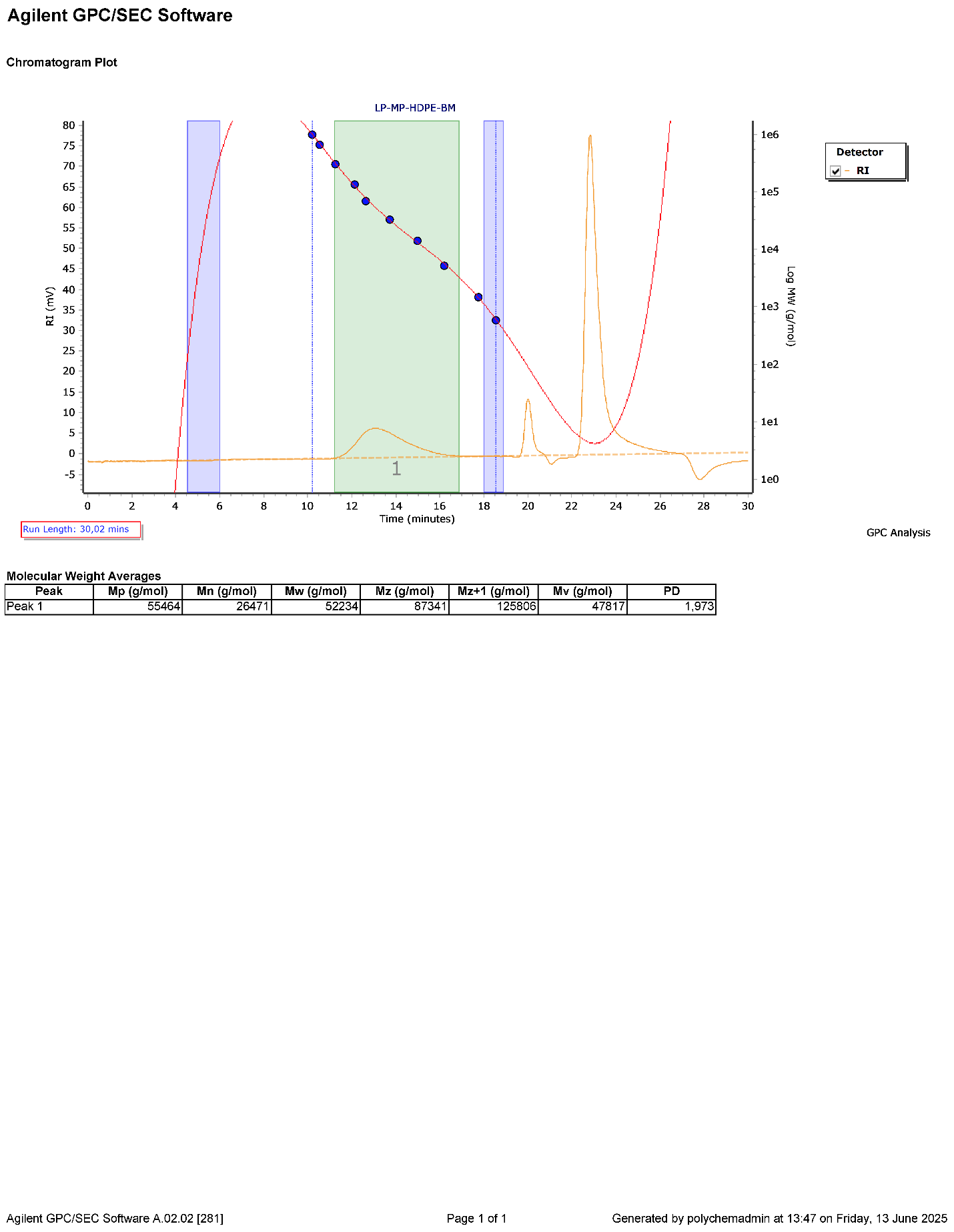


**Figure S39.** SEC of mechanical activated HDPE by ball-mill.

**
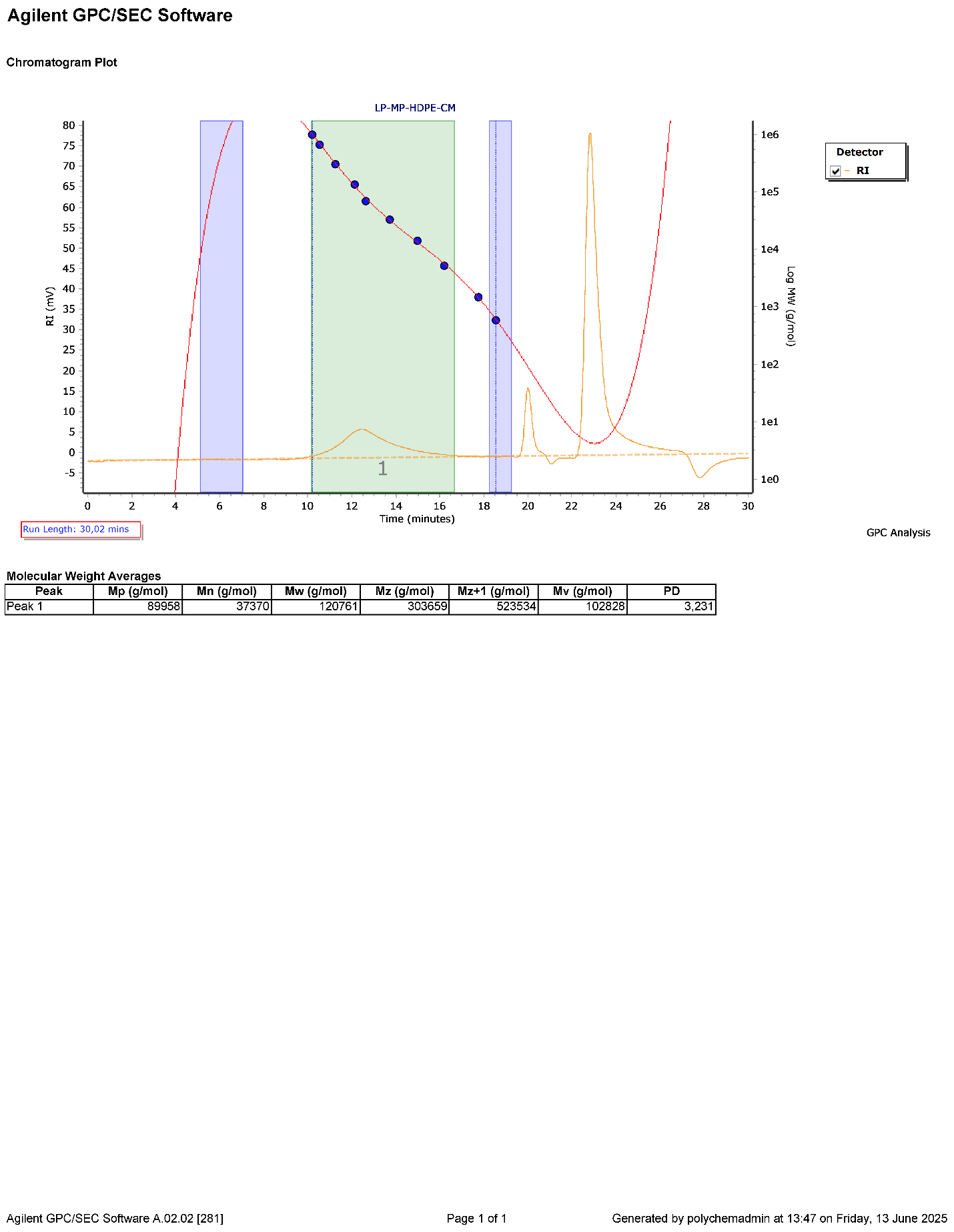
**

**Figure 40**. SEC of mechanical activated HDPE by cryo-mill.


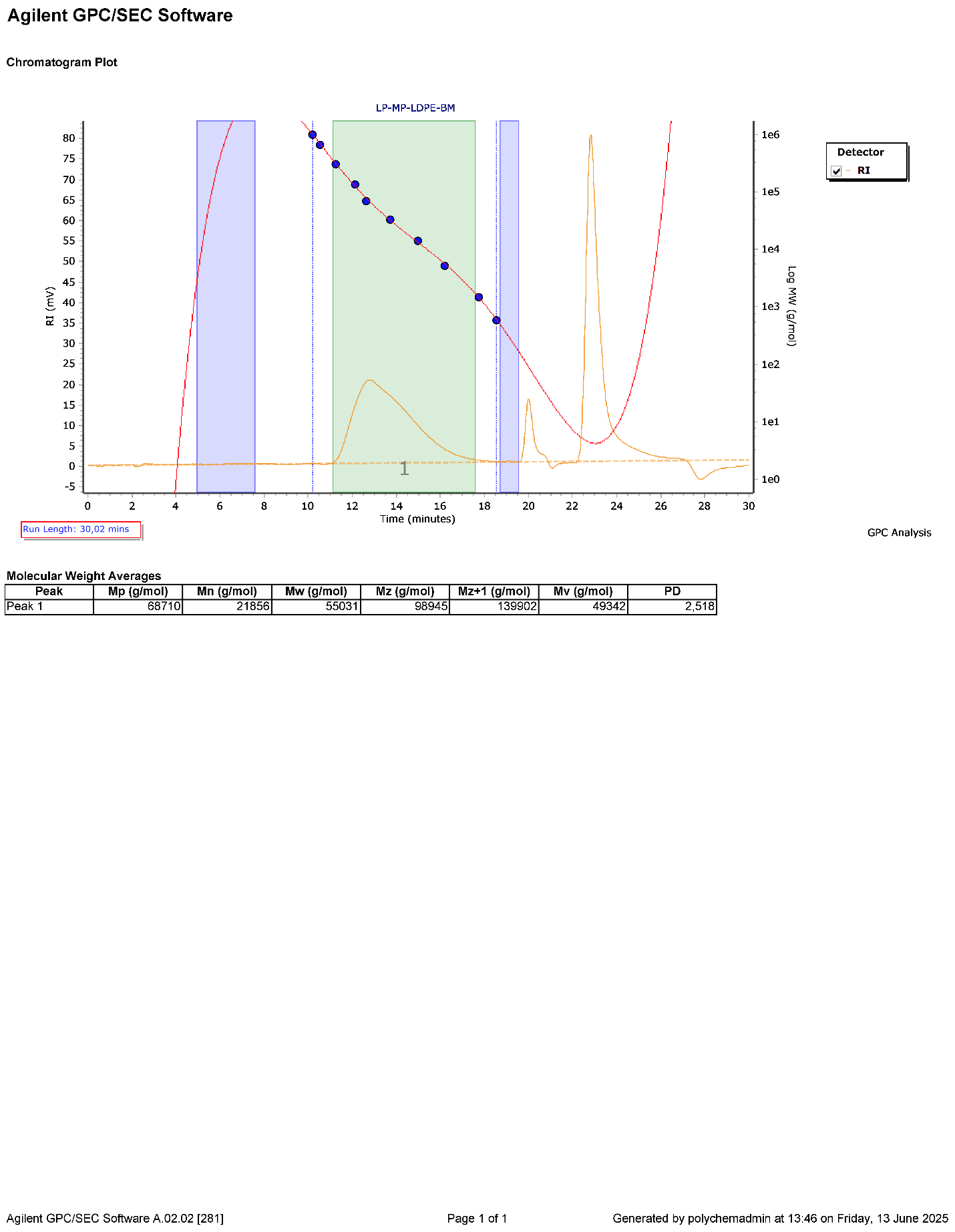


**Figure S41**. SEC of mechanical activated LDPE by ball-mill.

**
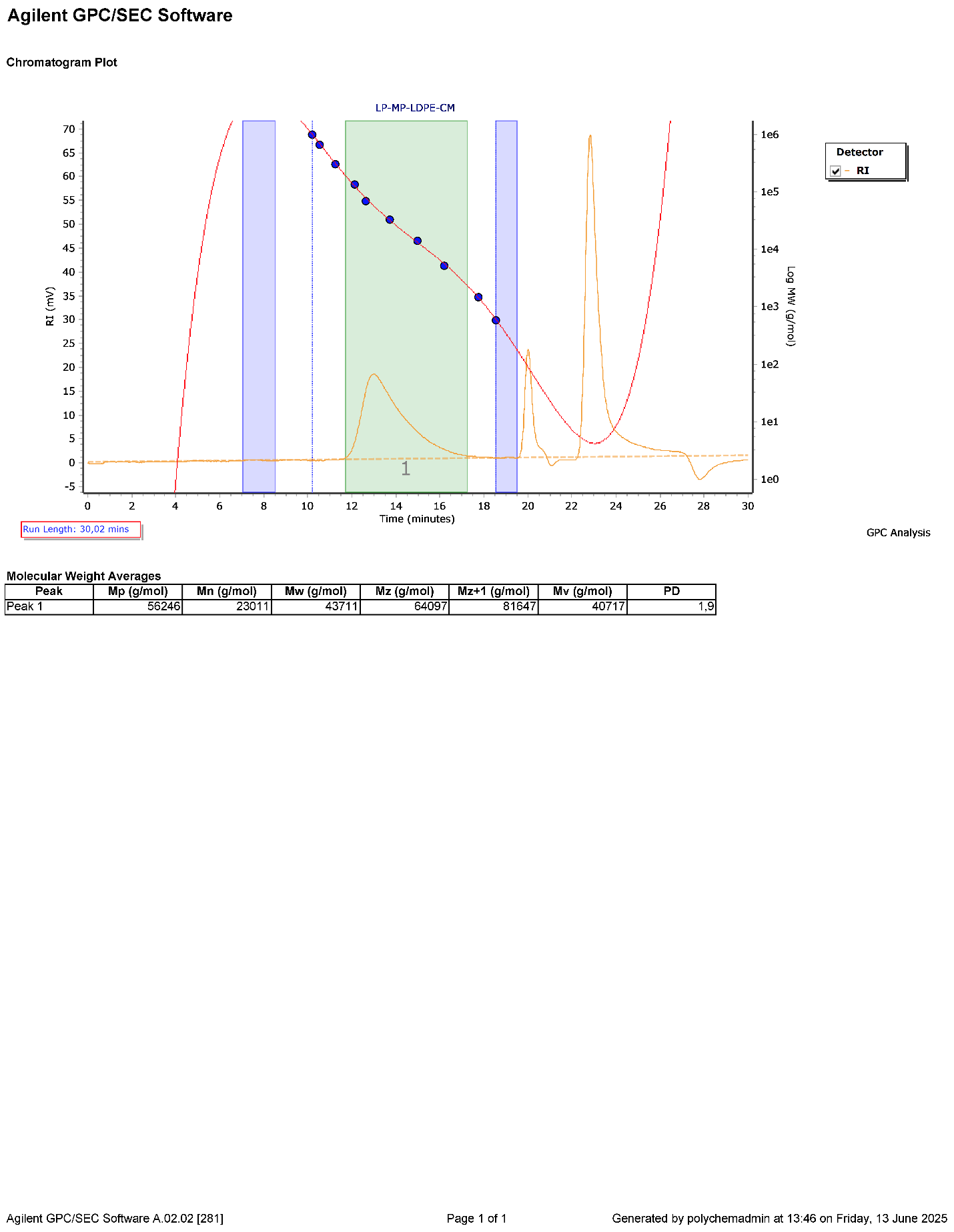
**

**Figure 42.** SEC of mechanical activated LDPE by cryo-mill.

# **SEC of degraded polymers**


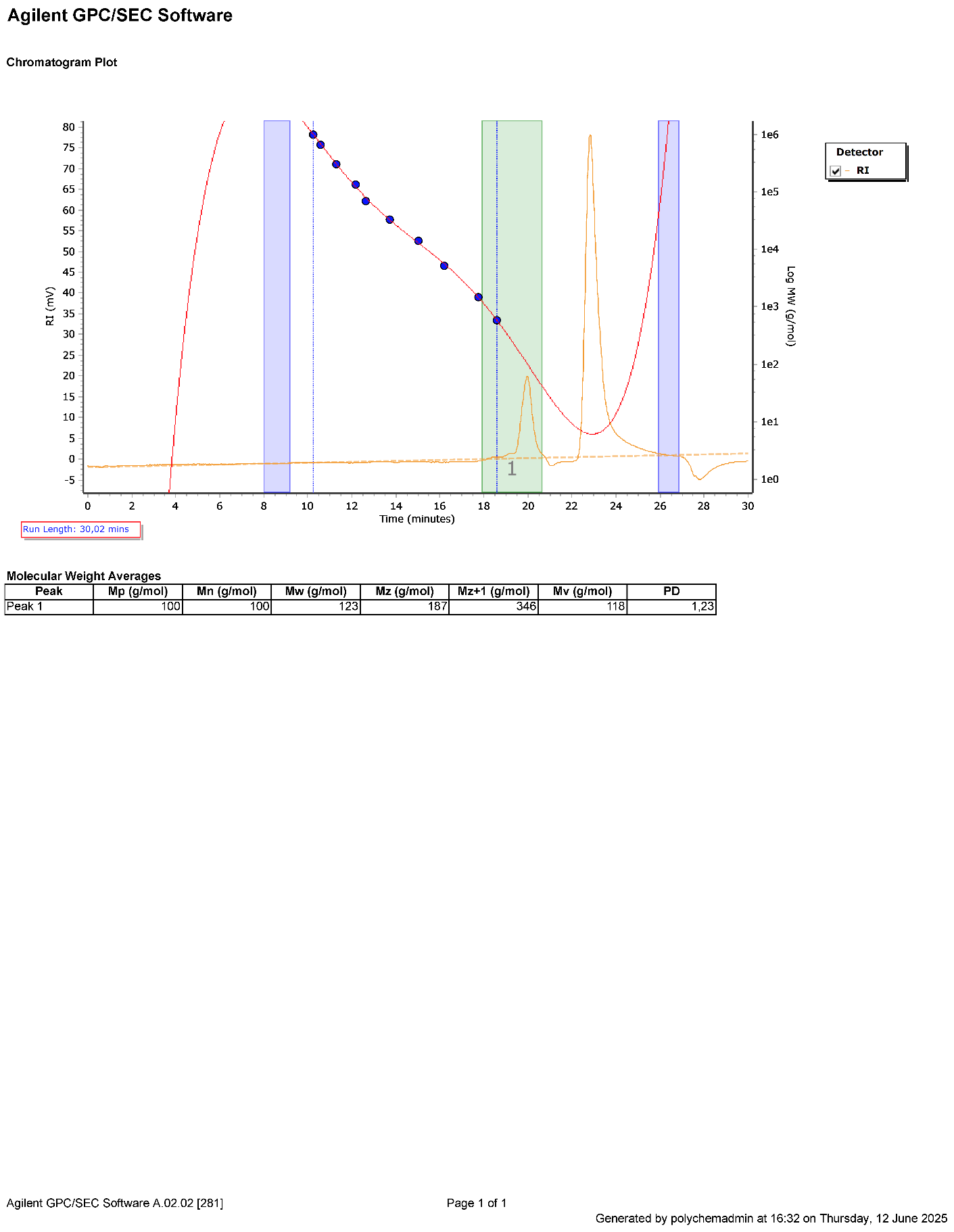


**Figure S43**. GPC of degraded P1 by ball-mill.


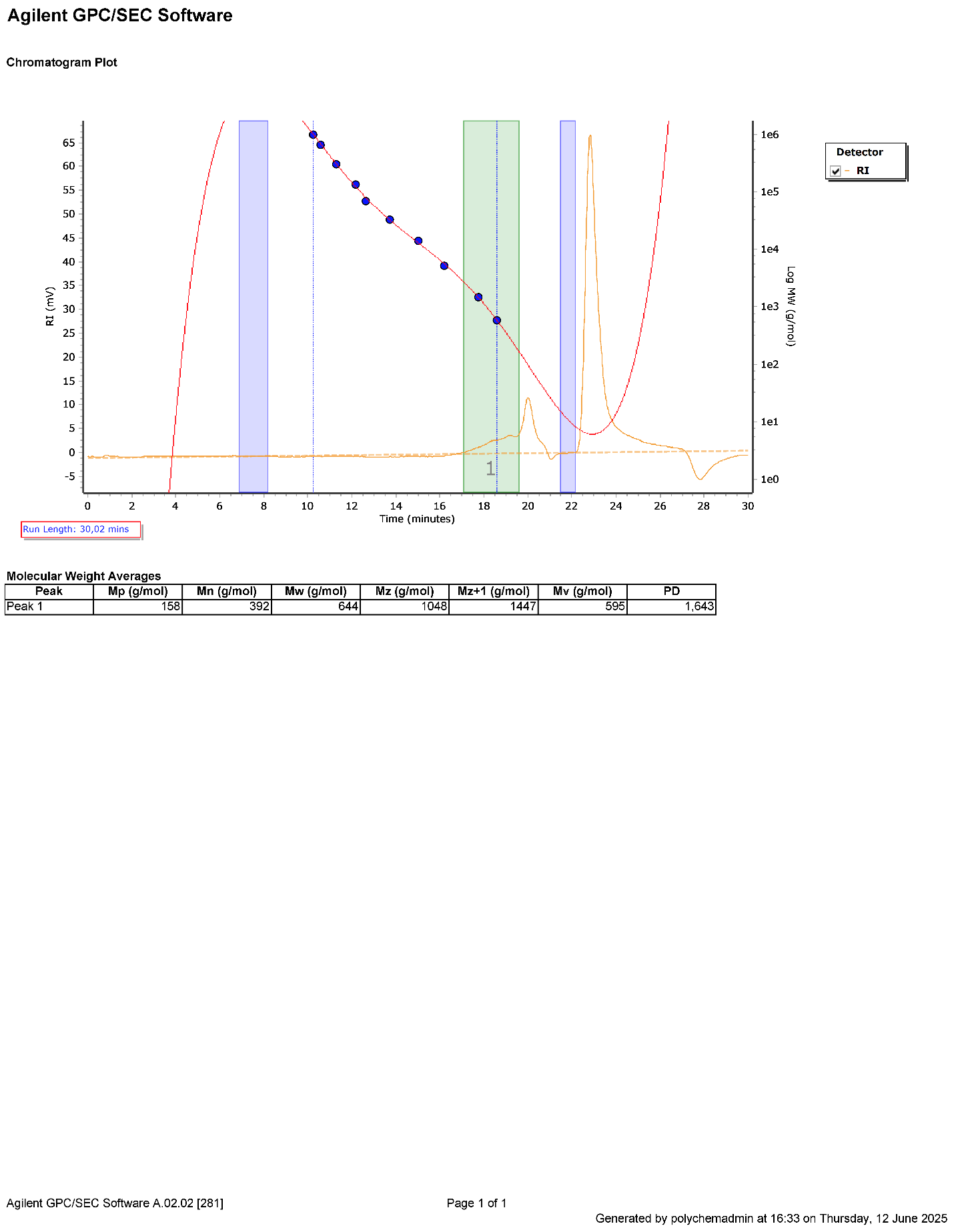


**Figure S44**. GPC of degraded P1 by cryo-mill.


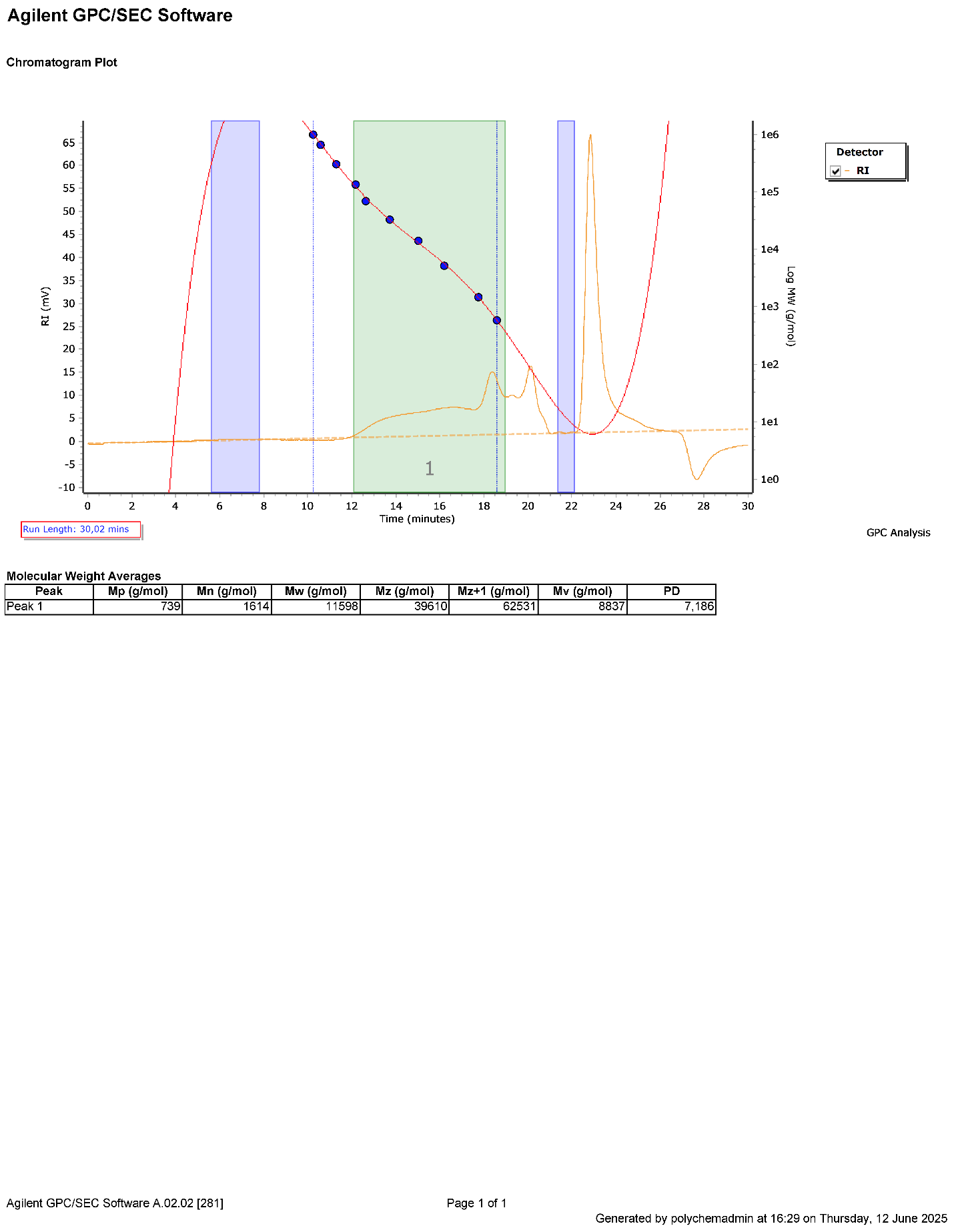


**Figure S45**. GPC of degraded P2 by ball-mill.


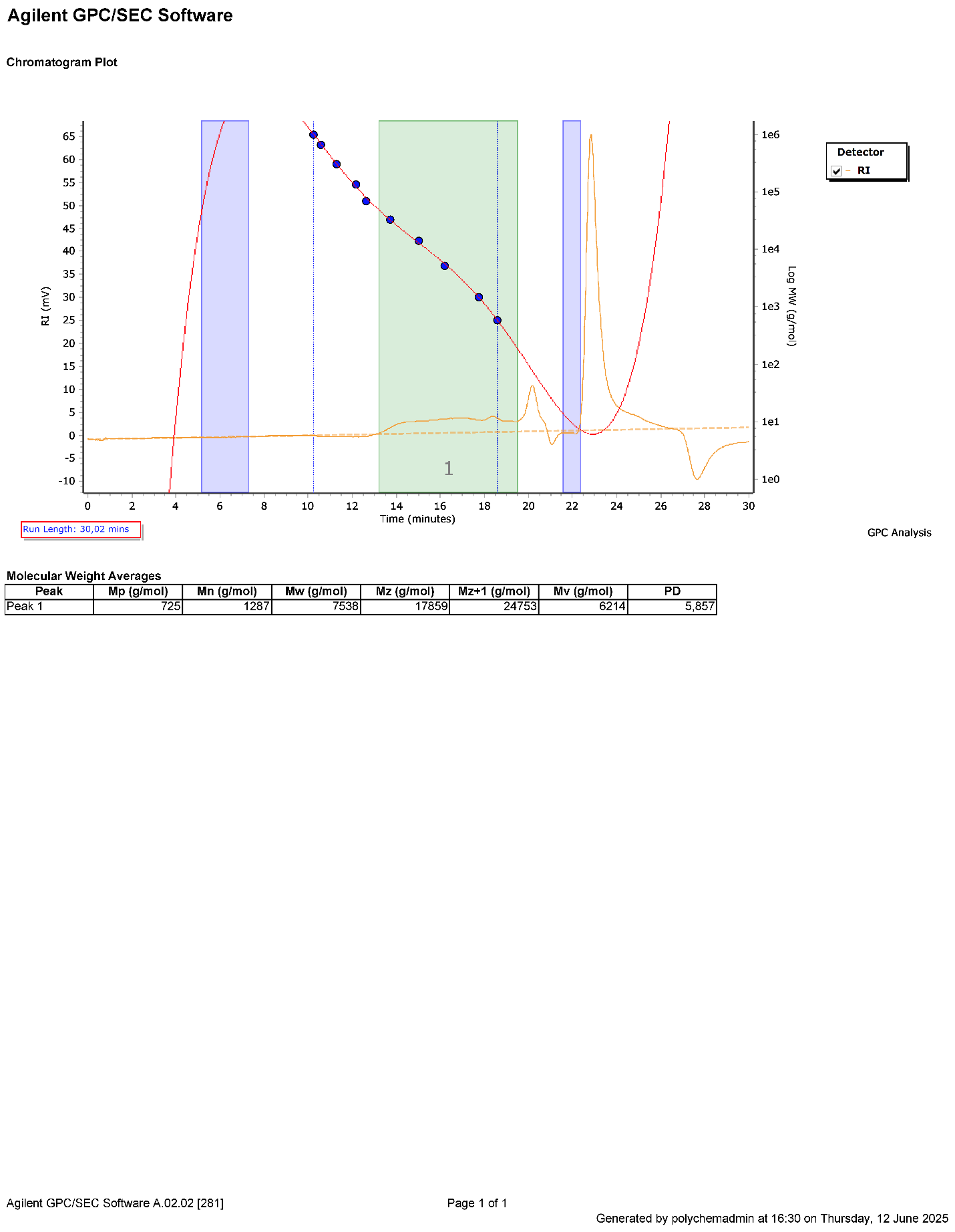


**Figure 46.** GPC of degraded P2 by cryo-mill.


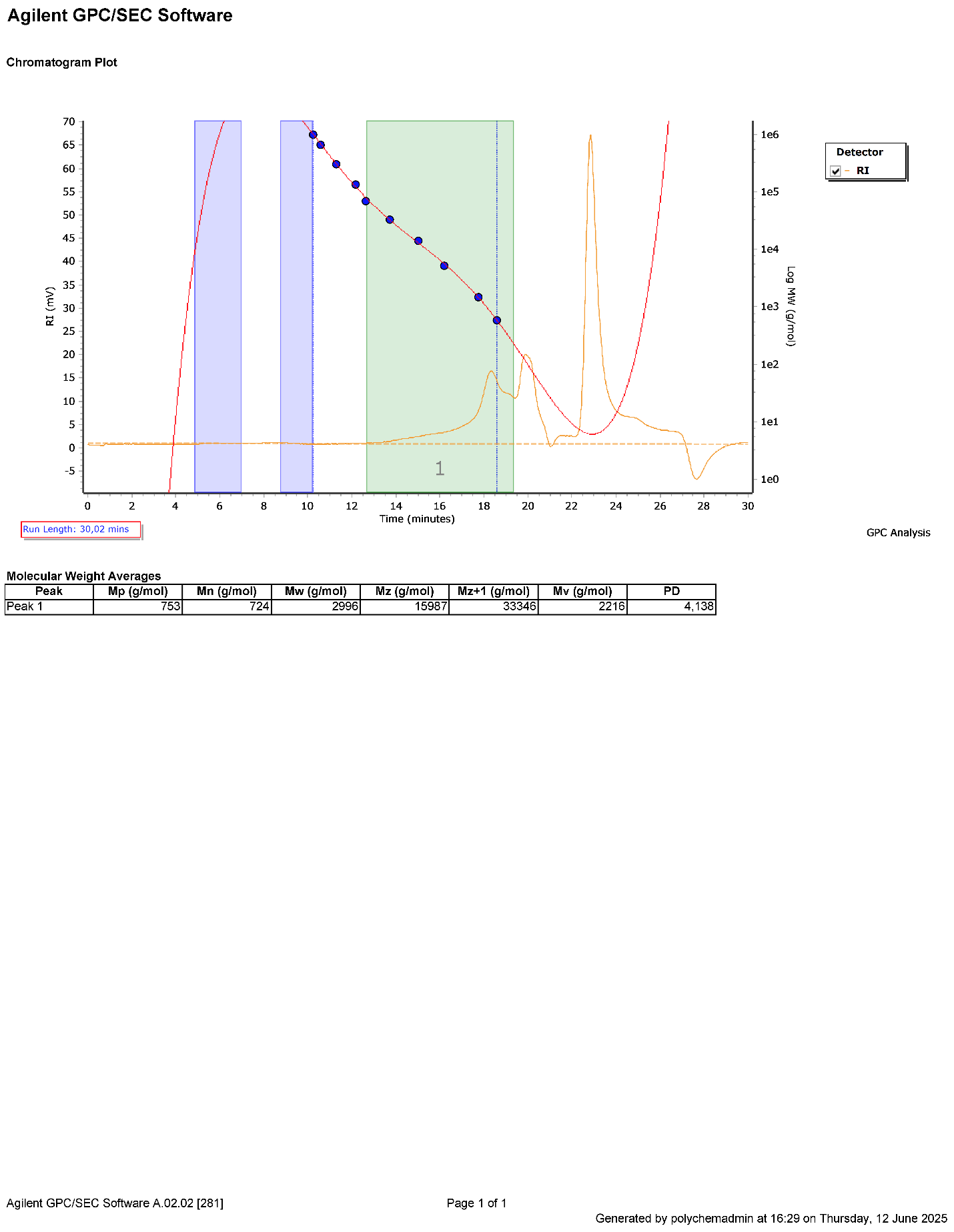


**Figure S47**. GPC of degraded P3 by ball-mill.


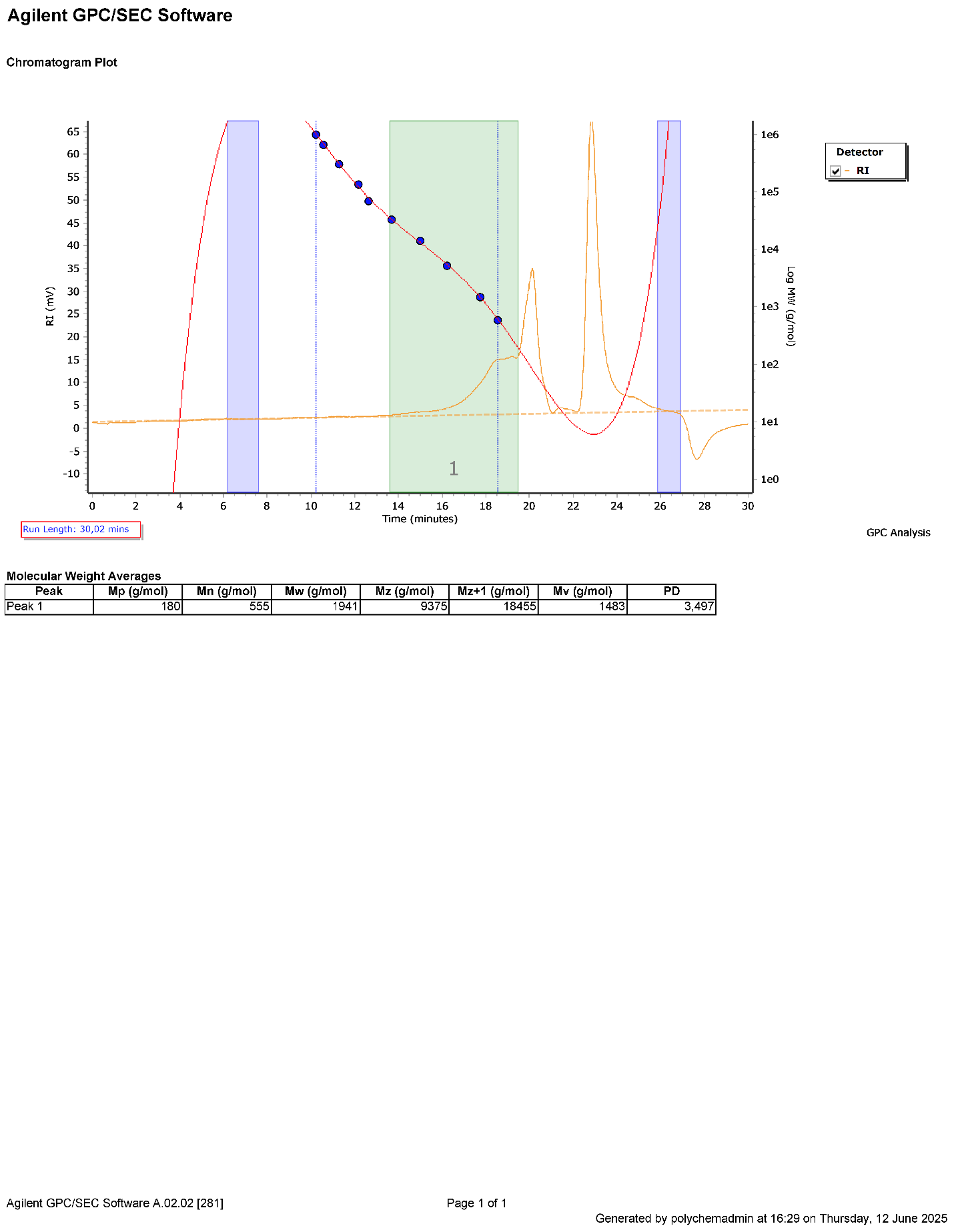


**Figure 48.** GPC of degraded P3 by cryo-mill.


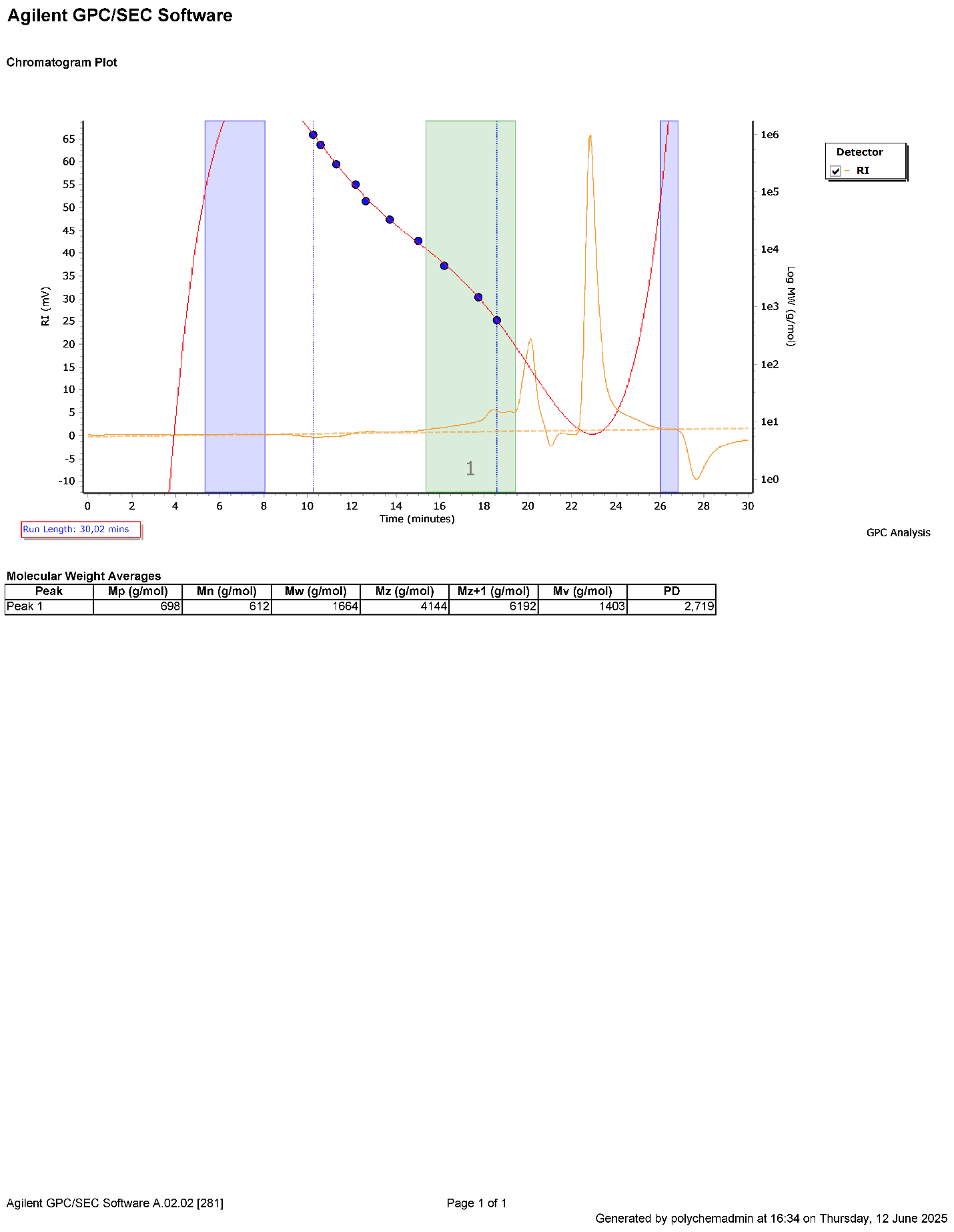


**Figure S49.** GPC of degraded P4 by ball-mill.


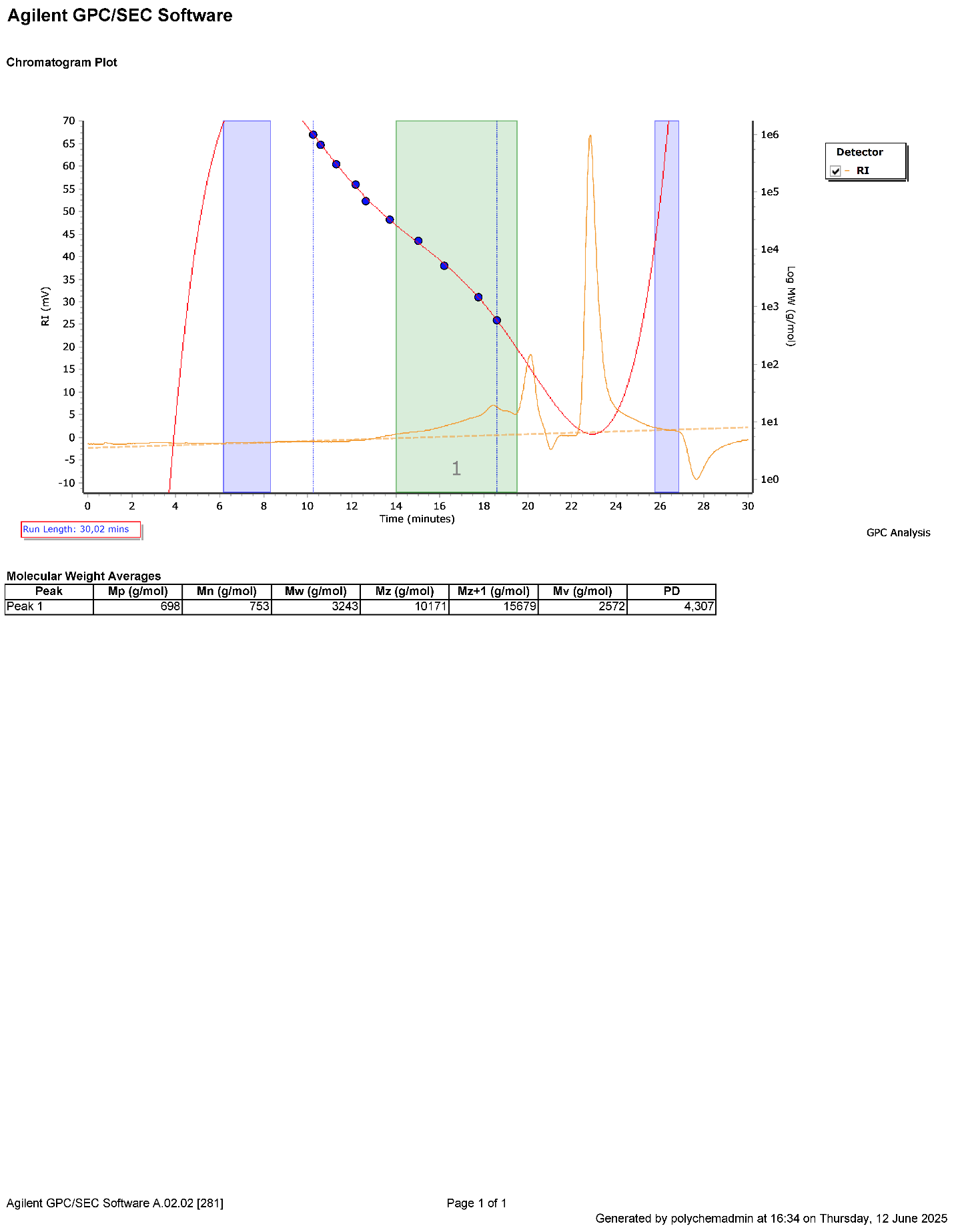


**Figure S50.** GPC of degraded P4 by cryo-mill.


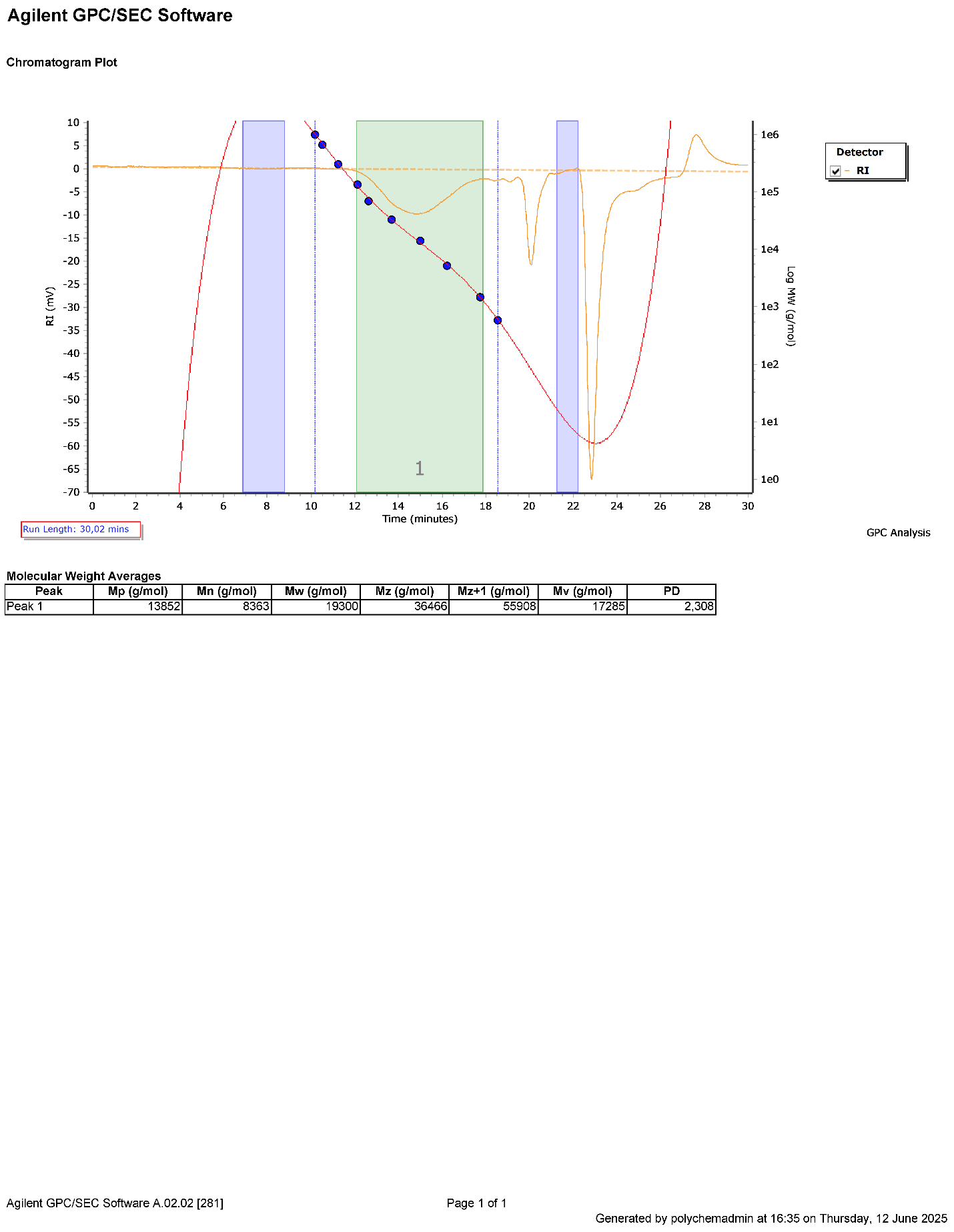


**Figure S51**. GPC of degraded P5 by ball-mill.


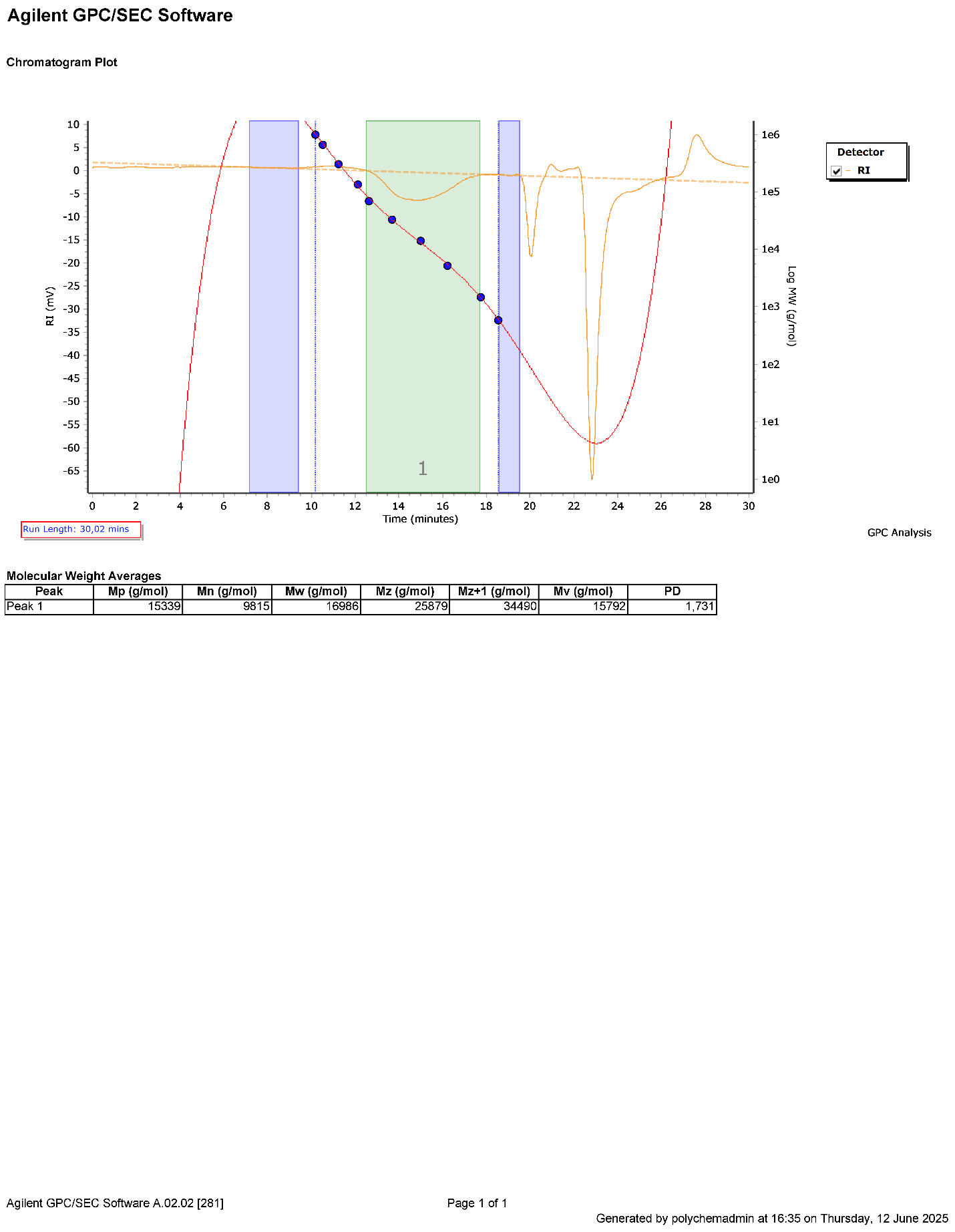


**Figure S52**. GPC of degraded P5 by cryo-mill.


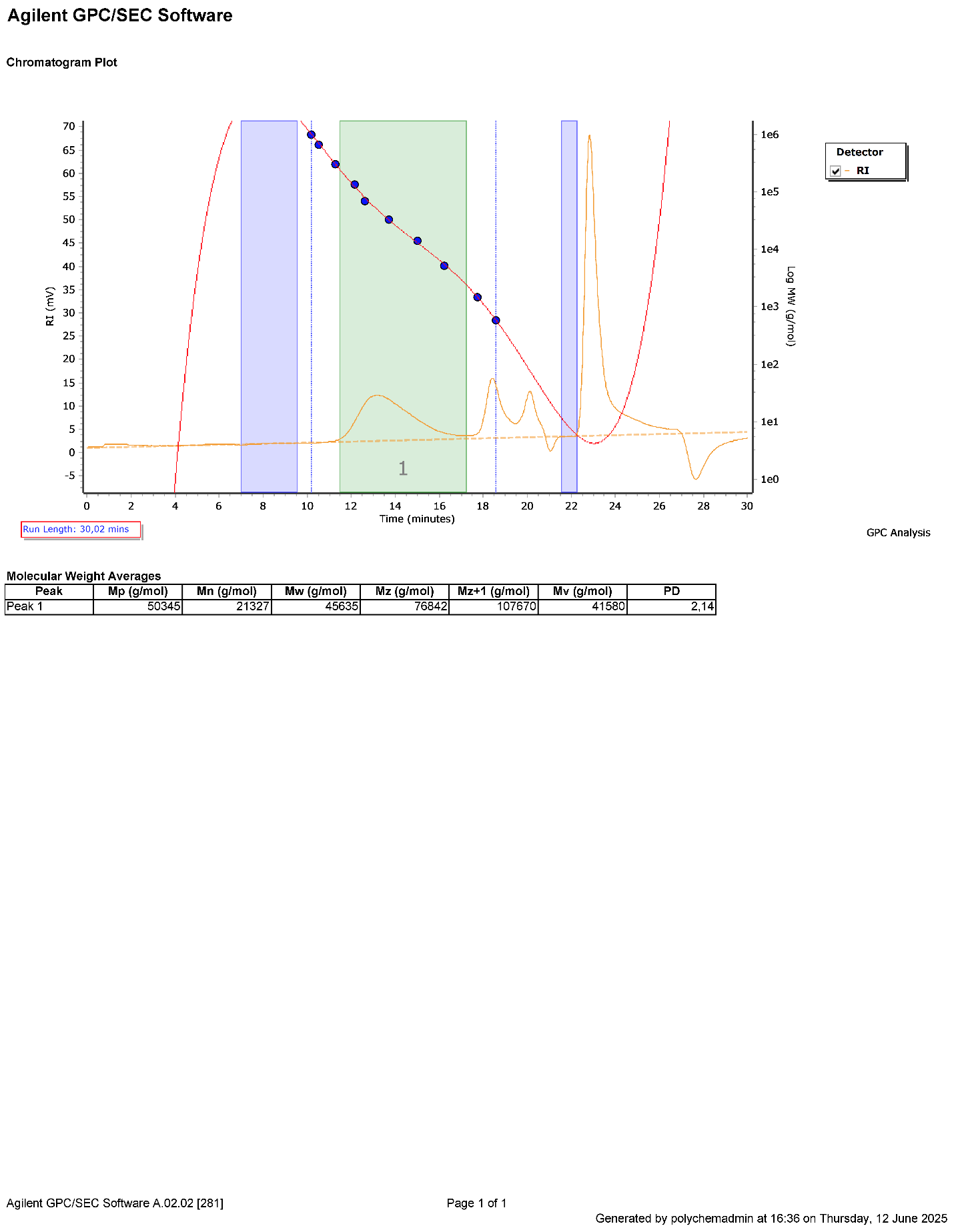


**Figure S53**. GPC of degraded P6 by ball-mill.


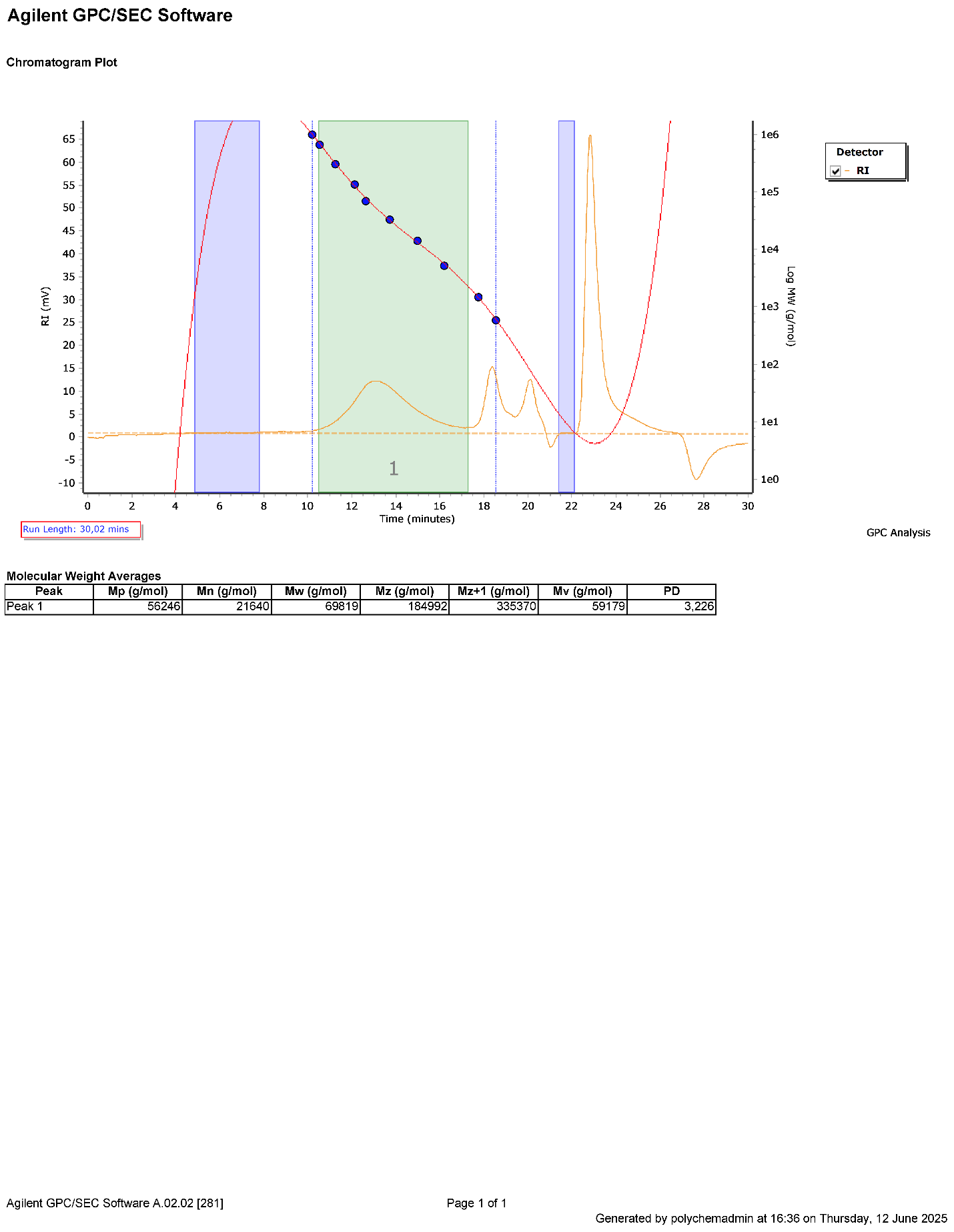


**Figure S54**. GPC of degraded P6 by cryo-mill.


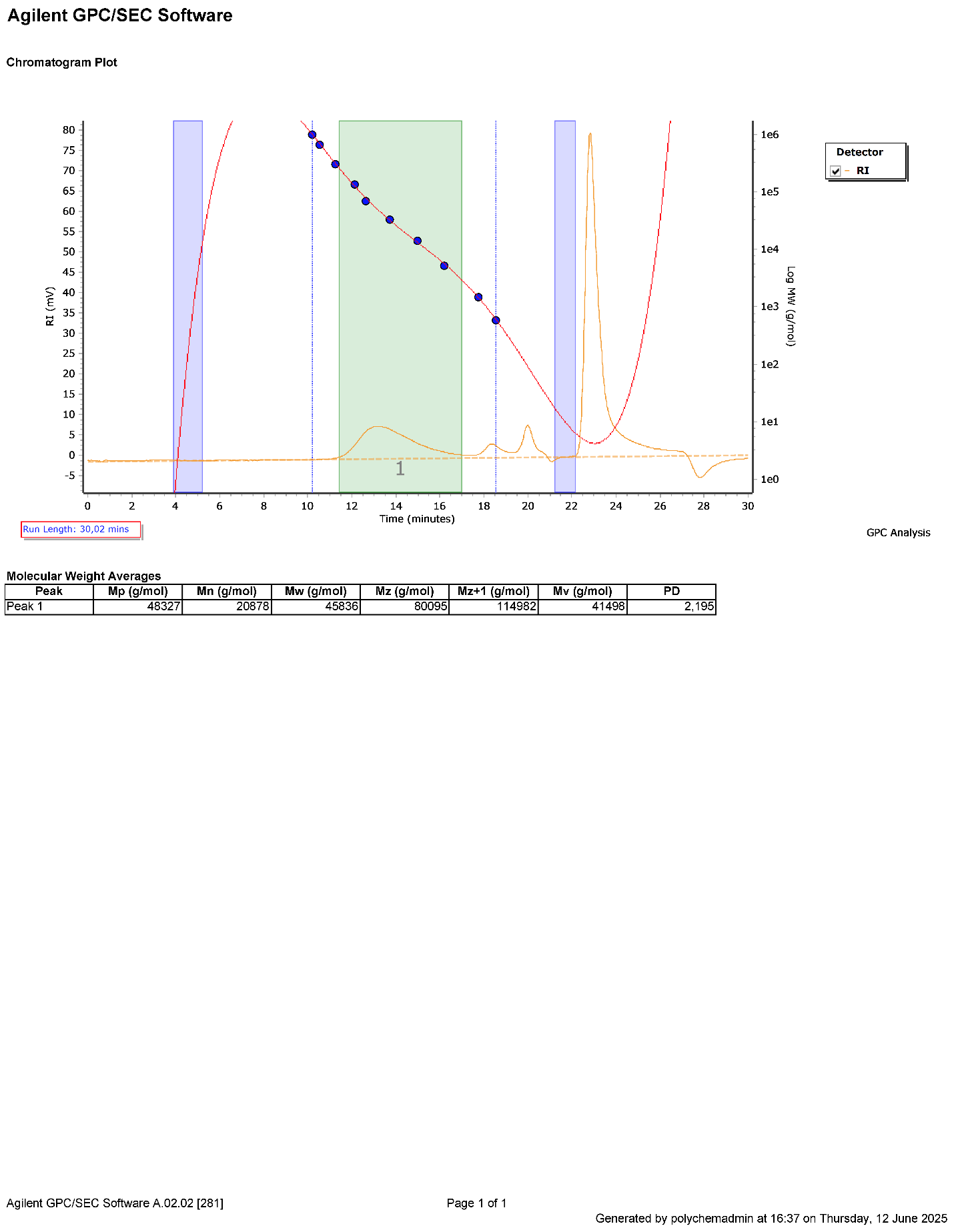


**Figure S55.** GPC of degraded HDPE by ball-mill.


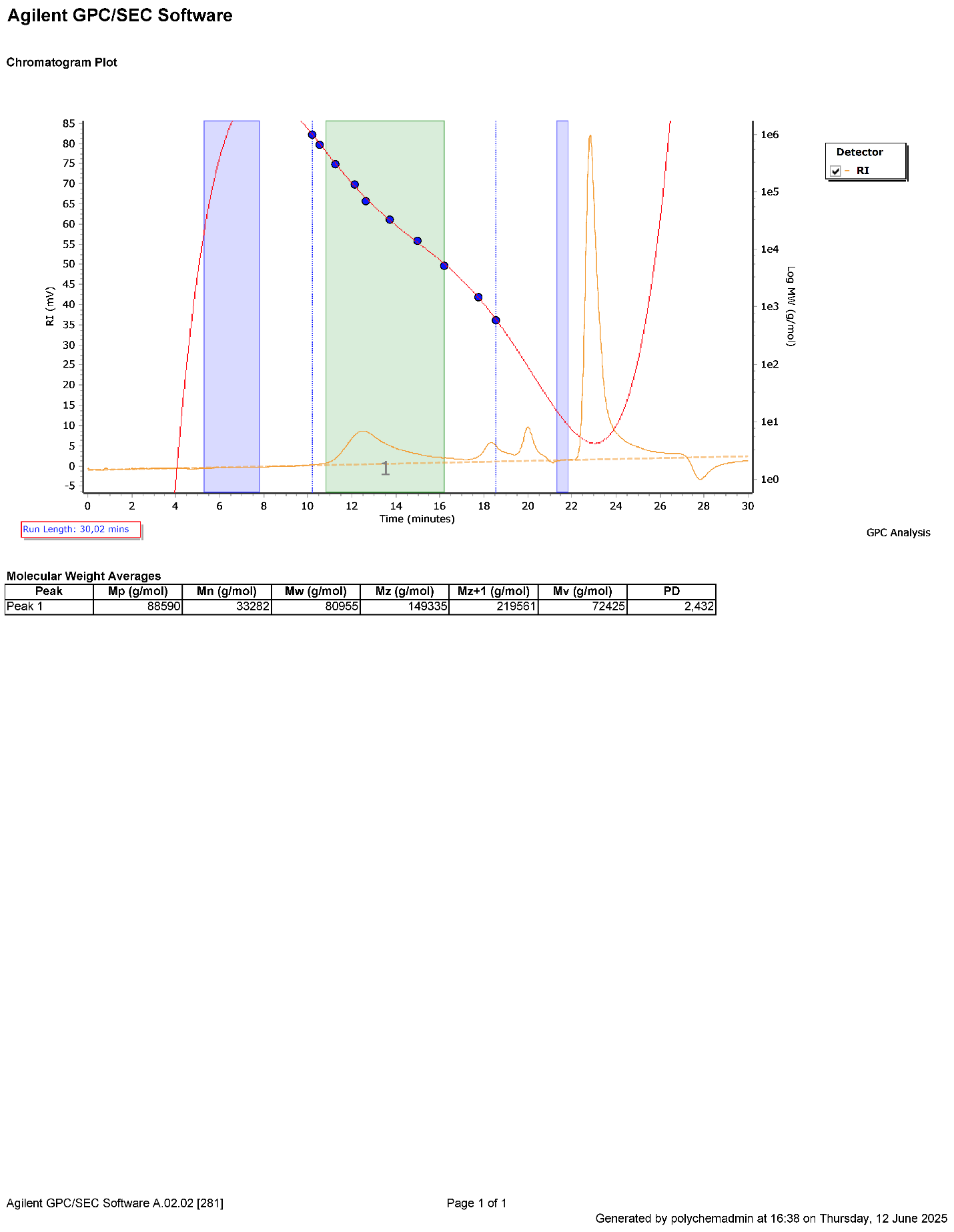


**Figure S56.** GPC of degraded HDPE by cryo-mill.


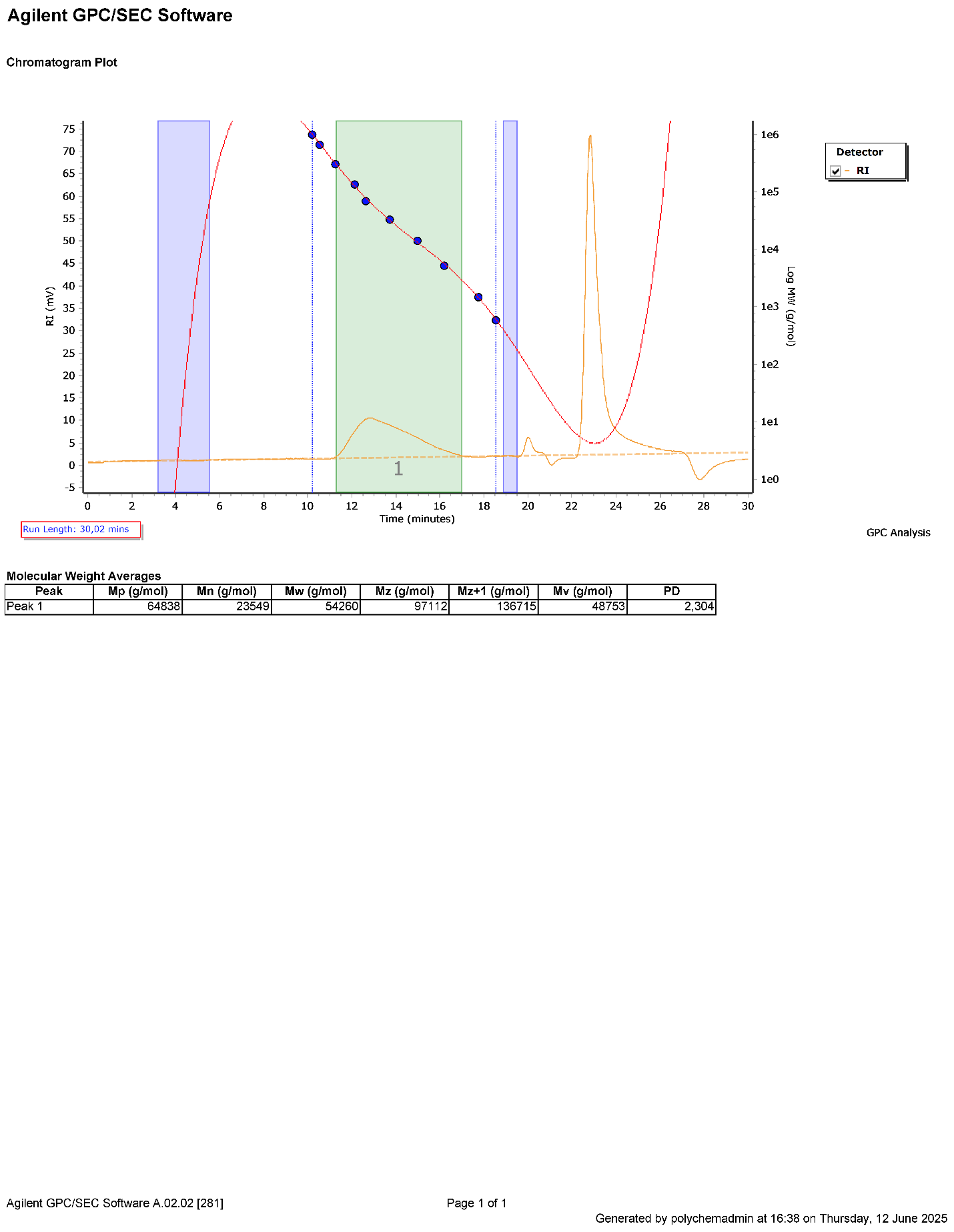


**Figure S57**. GPC of degraded LDPE by ball-mill.


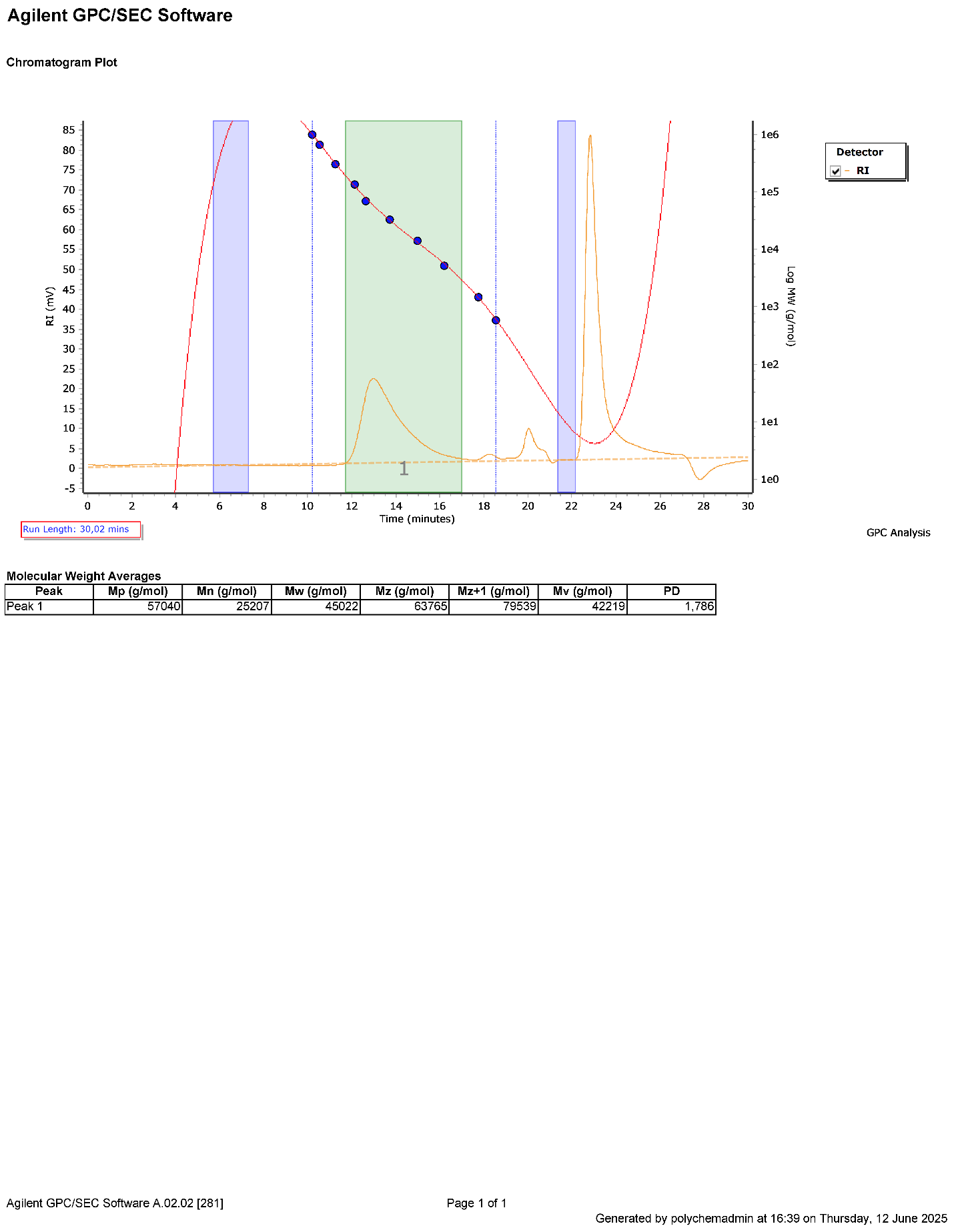


**Figure S58**. GPC of degraded LDPE by cryo-mill.

# **TGA of copolymers**


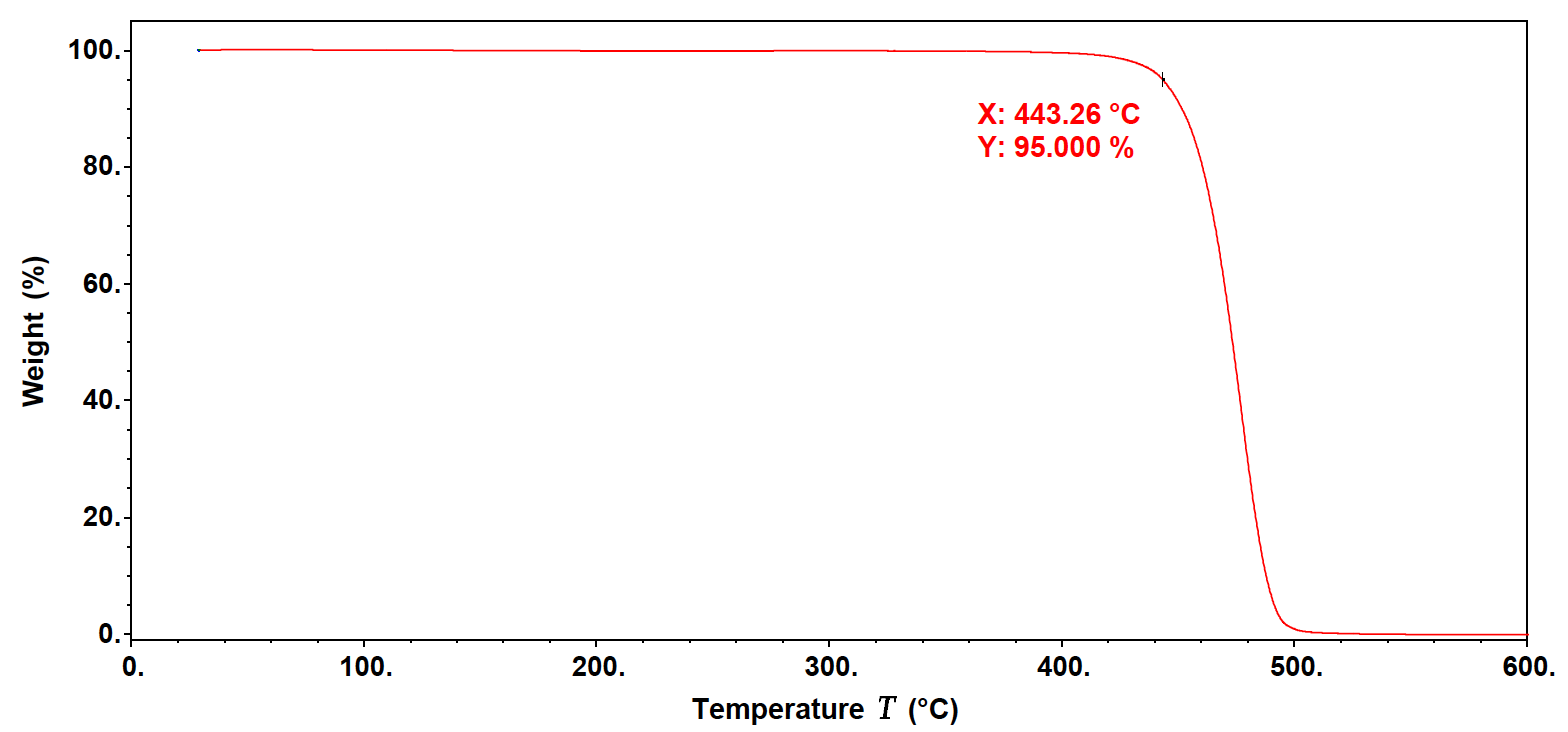


**Figure S59**. TGA trace of the P1.


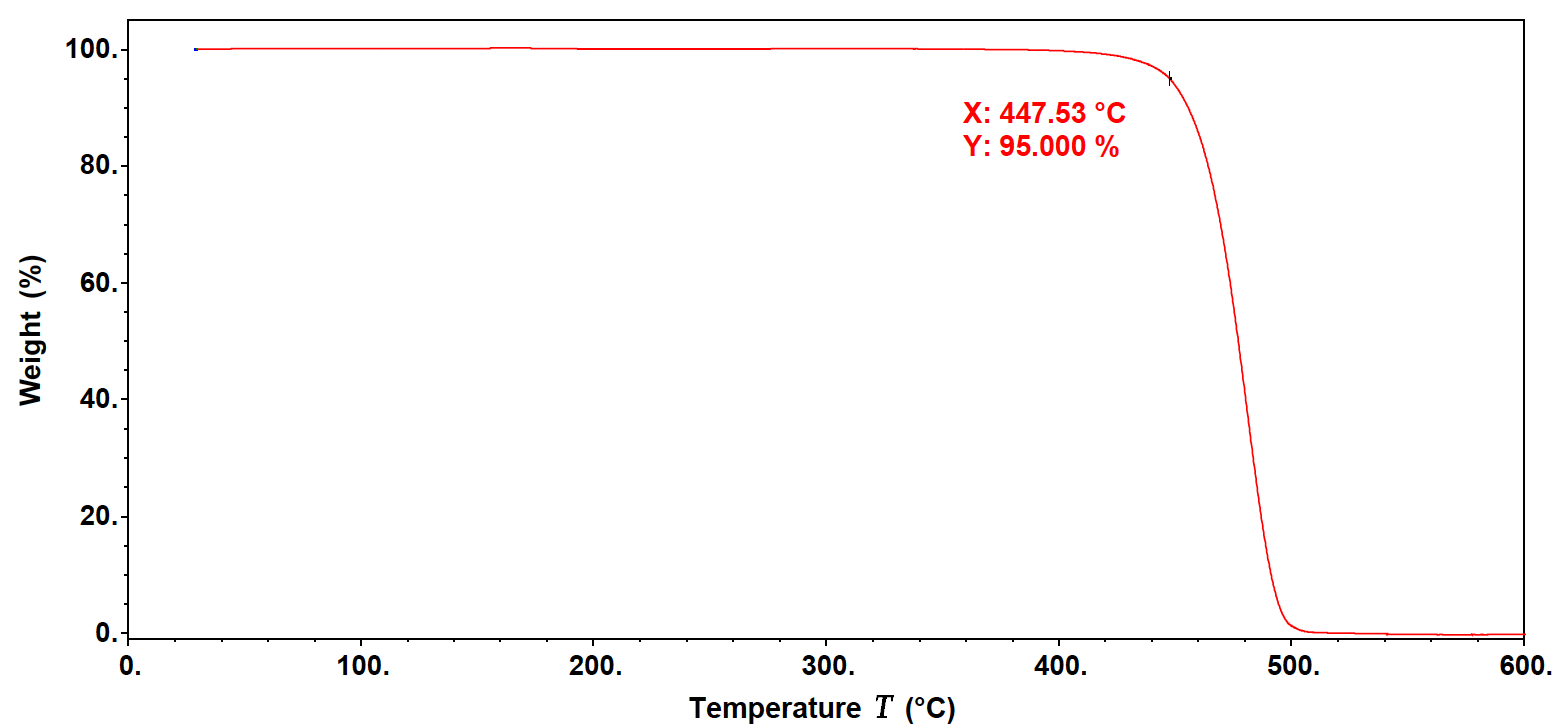


**Figure S60**. TGA trace of the P2.


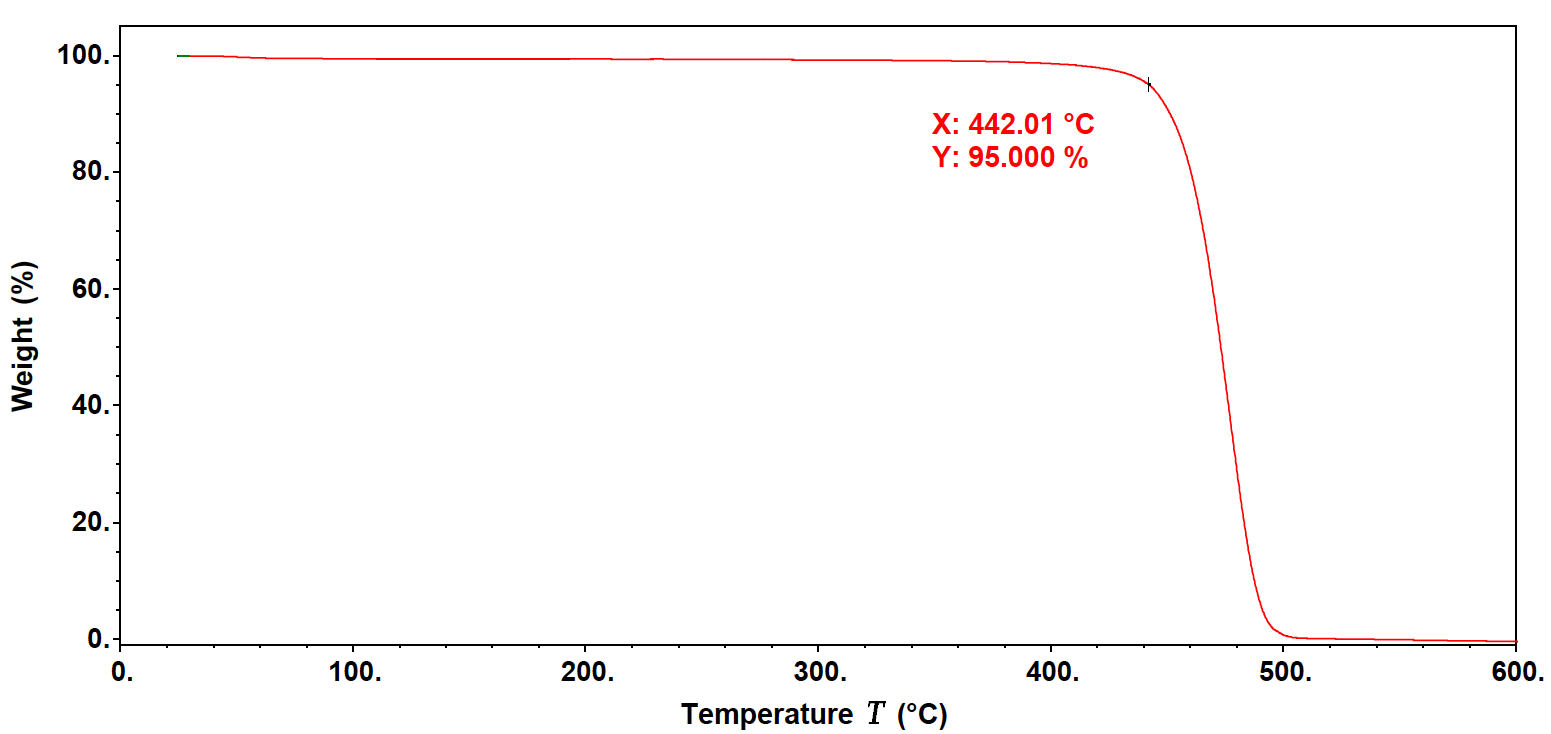


**Figure S61**. TGA trace of the P3.


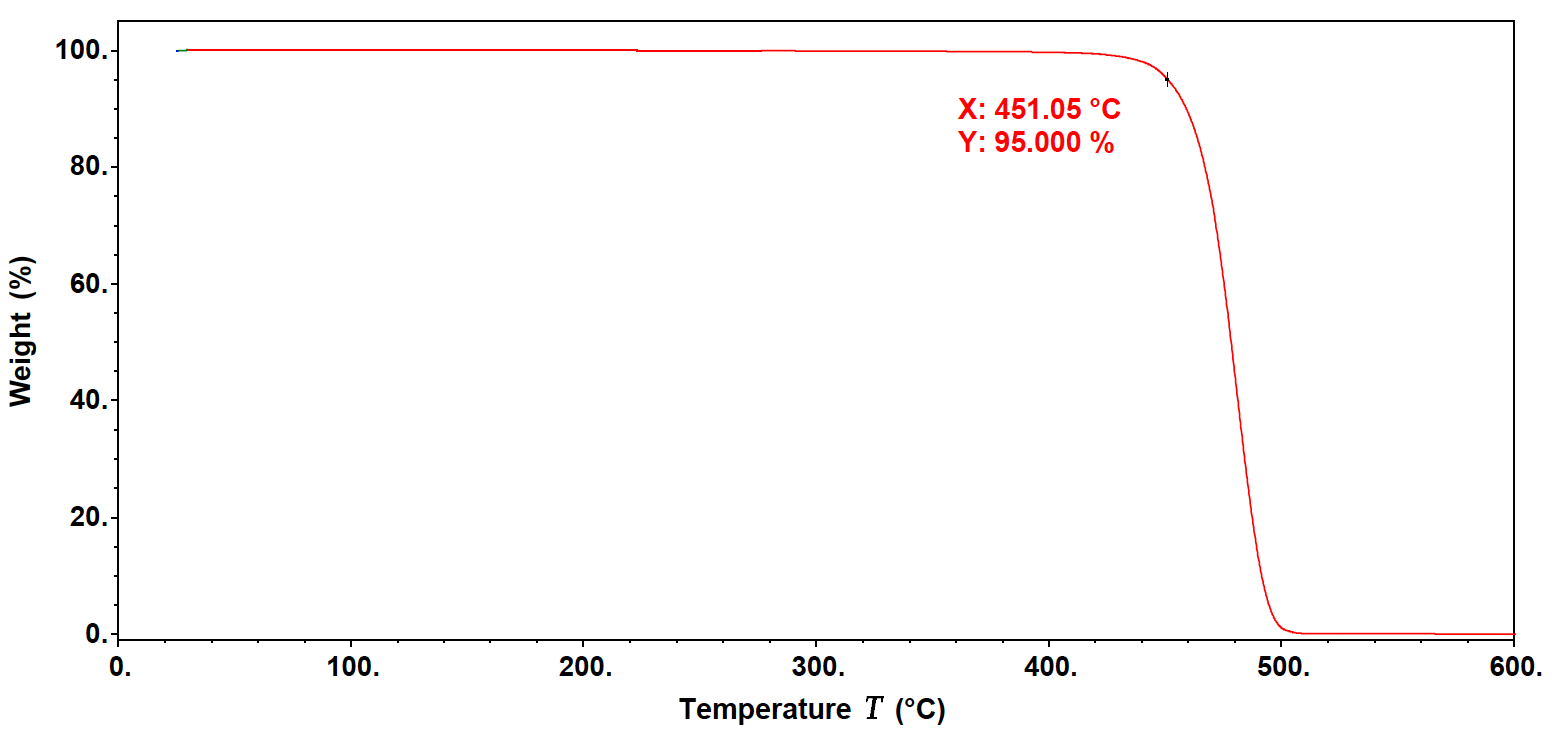


**Figure S62**. TGA trace of the P4.


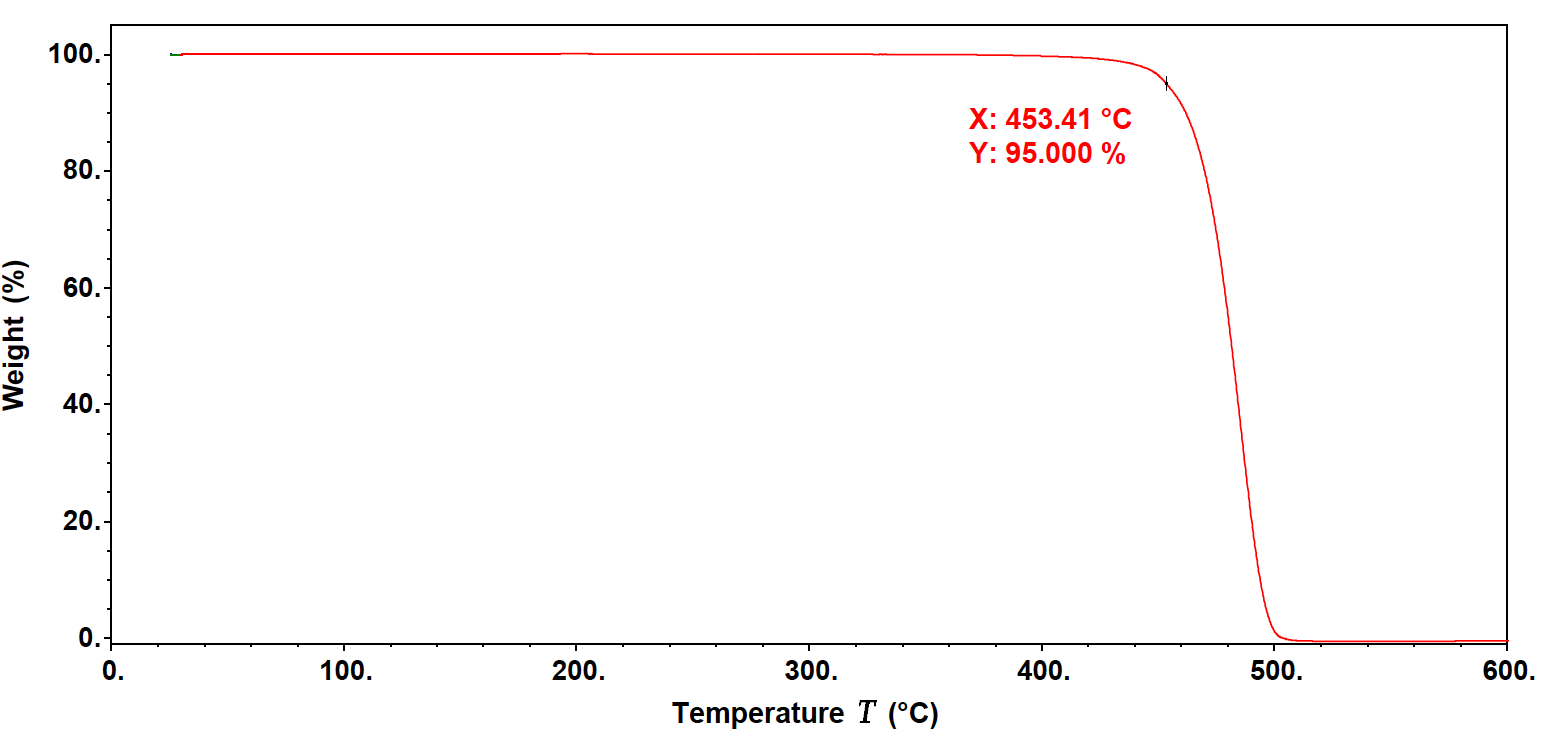


**Figure S63**. TGA trace of the P5.


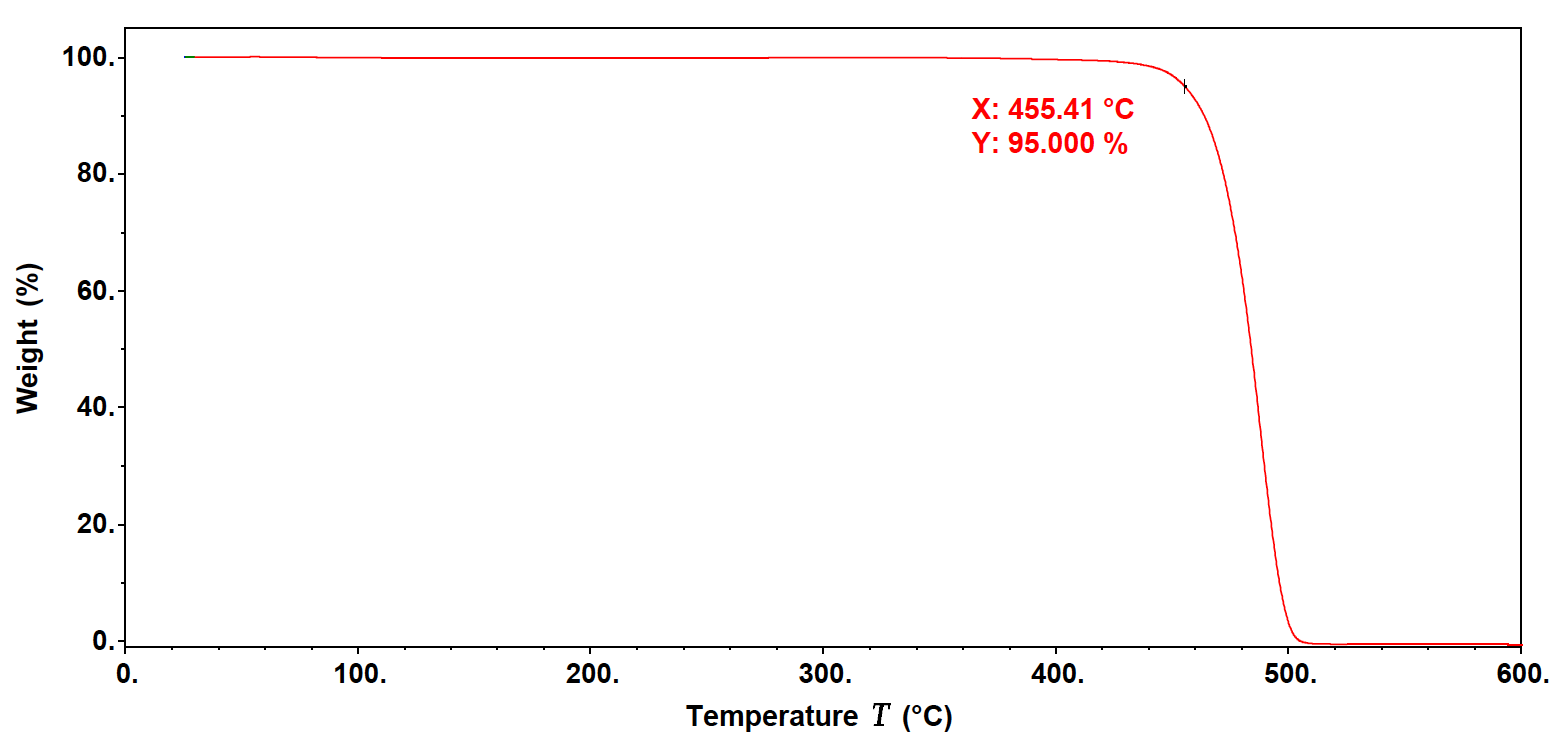


**Figure S64**. TGA trace of the P6.


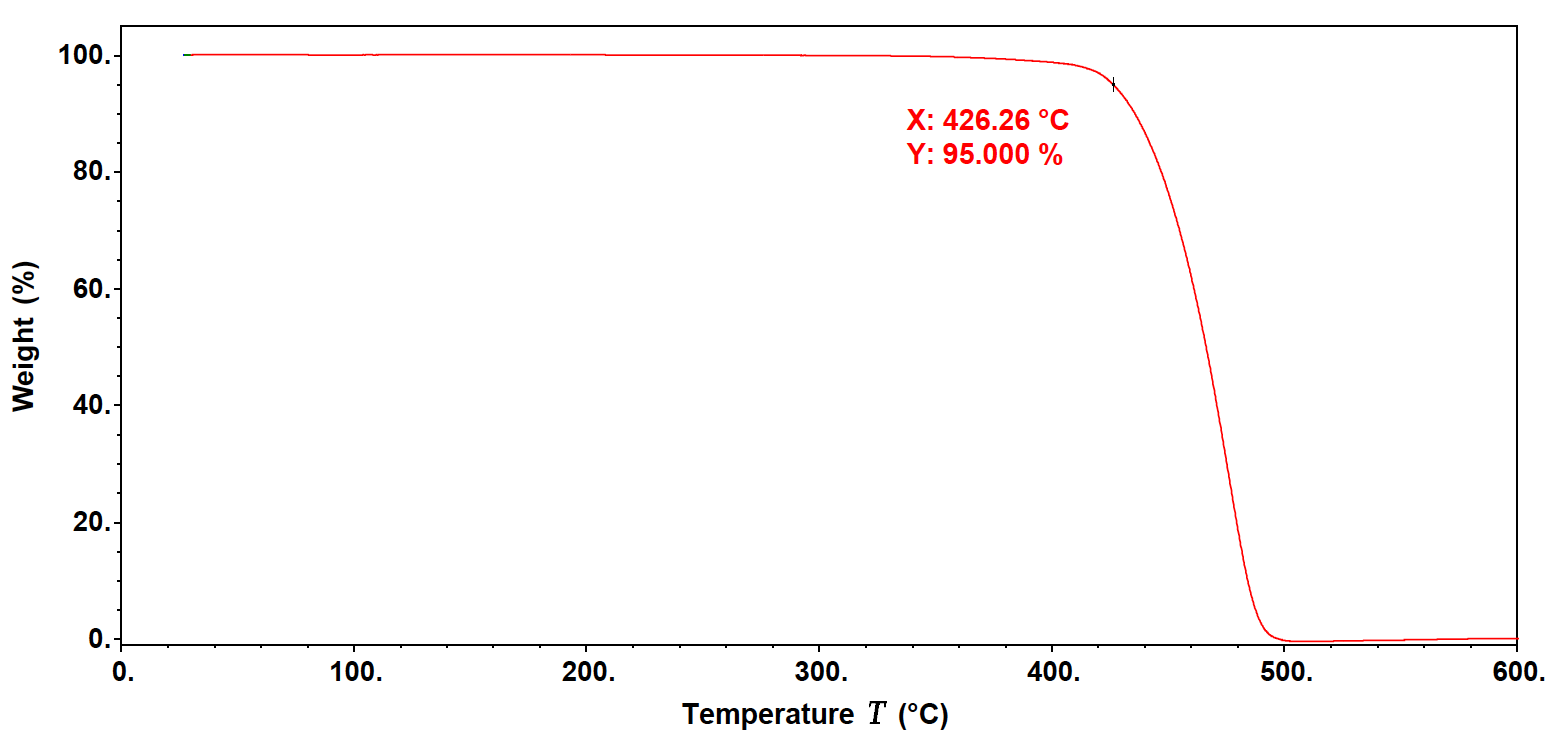


**Figure S65**. TGA trace of commercial HDPE.


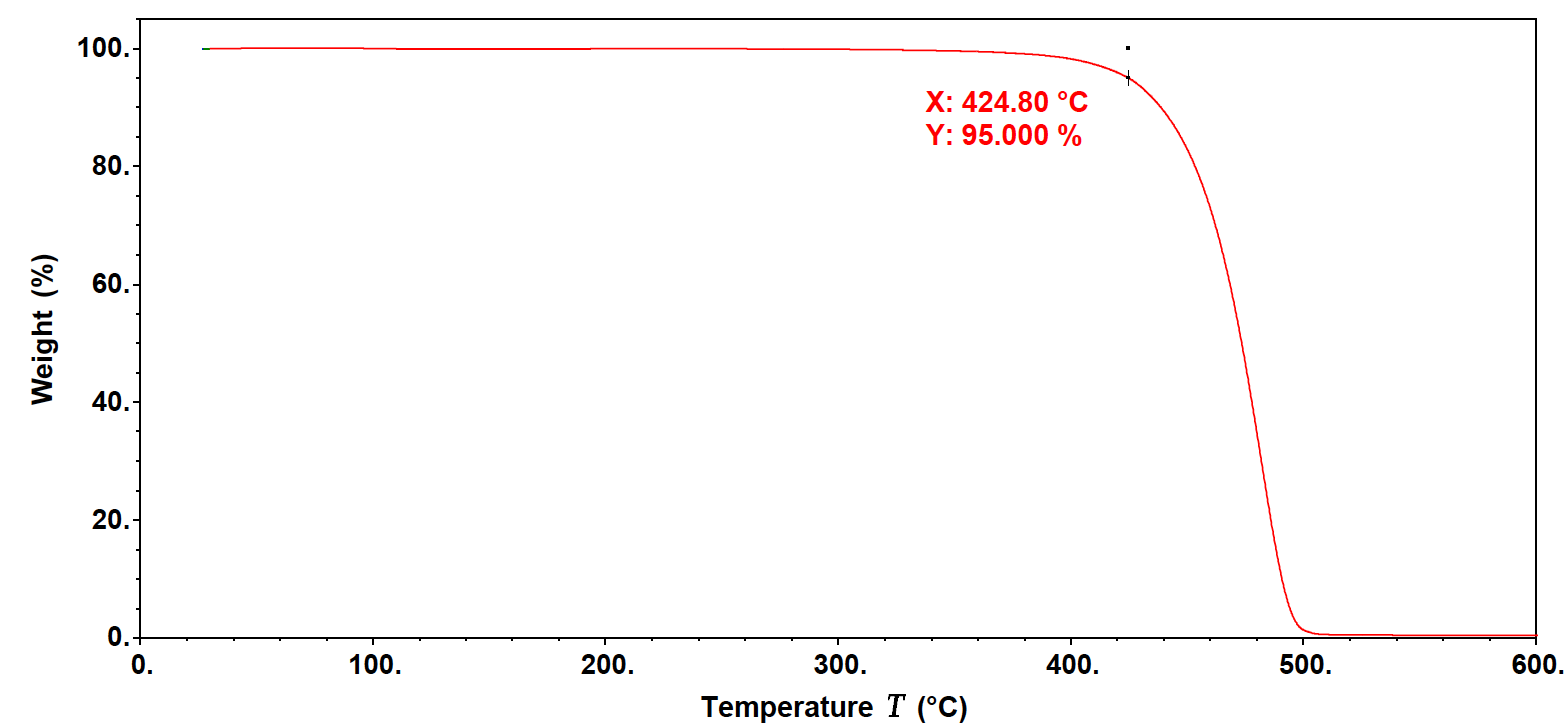


**Figure S66**. TGA trace of commercial LDPE


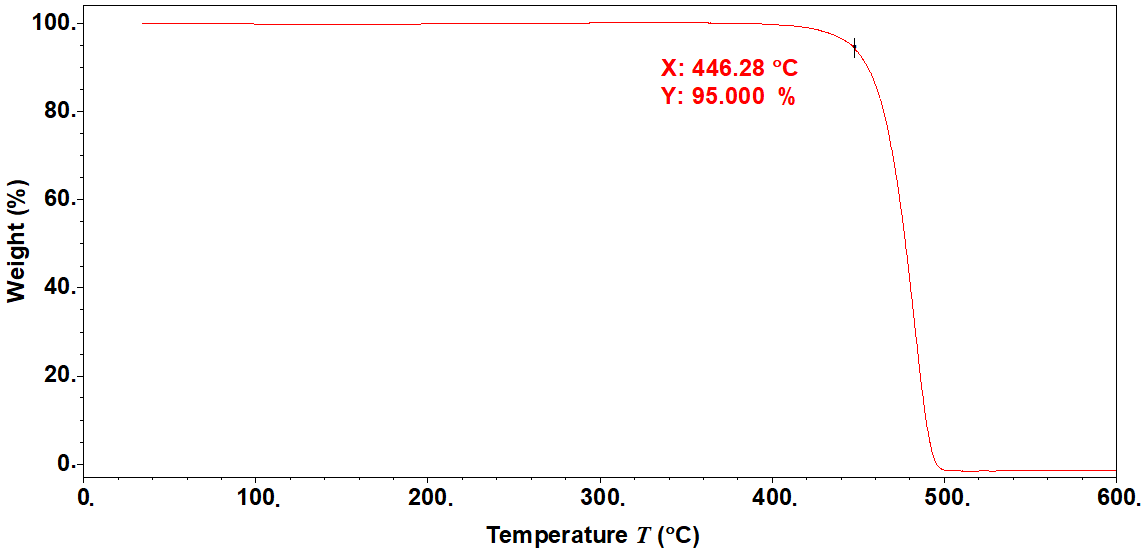


**Figure S67**. TGA trace of commercial XLPE


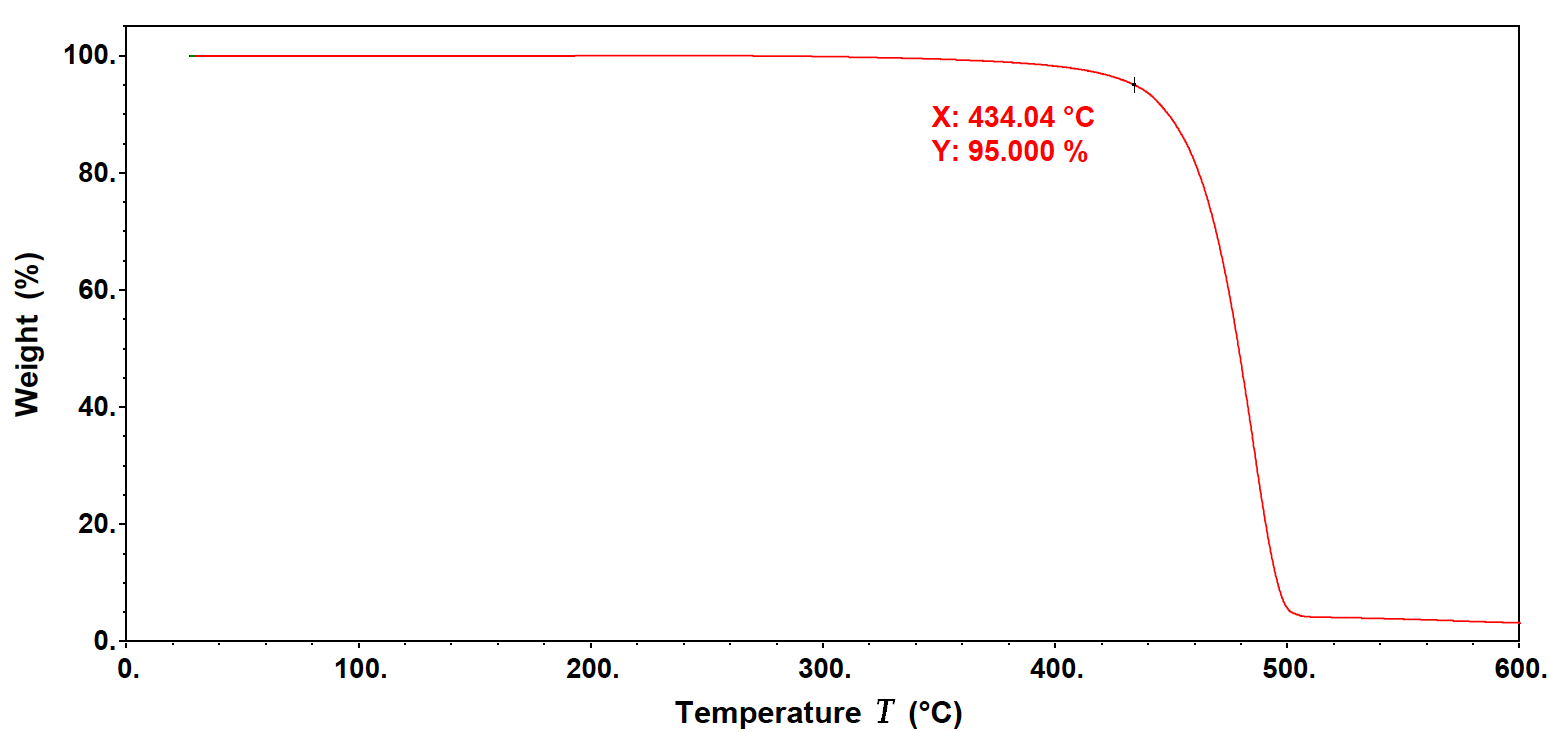


**Figure S68**. TGA trace of the crosslinked PE-like polymer (P6-ReXL) from the repolymerization of degraded fractions of P6.

**Figure S69**. TGA trace of the crosslinked P6c (crosslinked P6).

**Figure S70**. TGA trace of the crosslinked PE-like polymer (P6c-ReXL) from the repolymerization of degraded fractions of P6c.

# **DSC of copolymers**


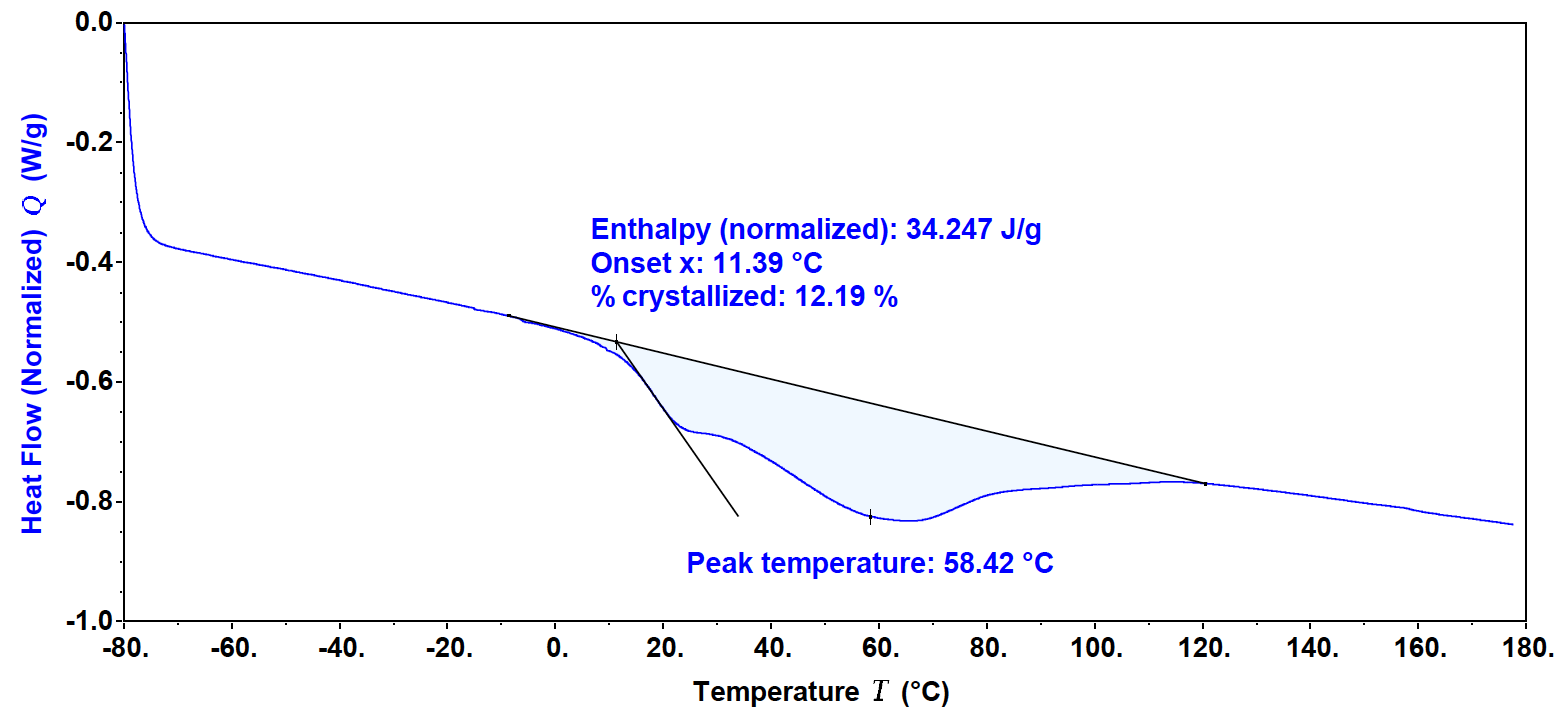


**Figure S71**. DSC trace of P1.

**
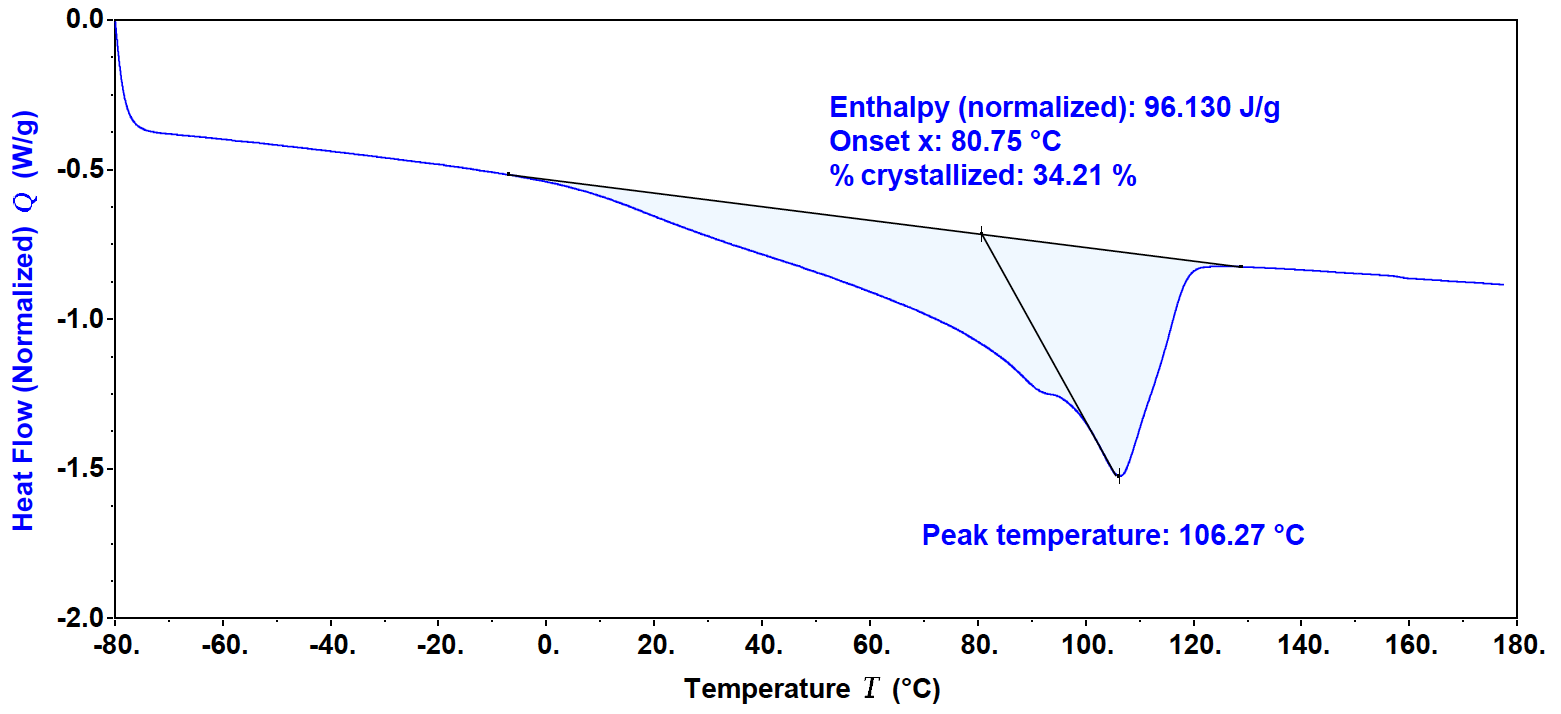
**

**Figure S72**. DSC trace of P2.

**
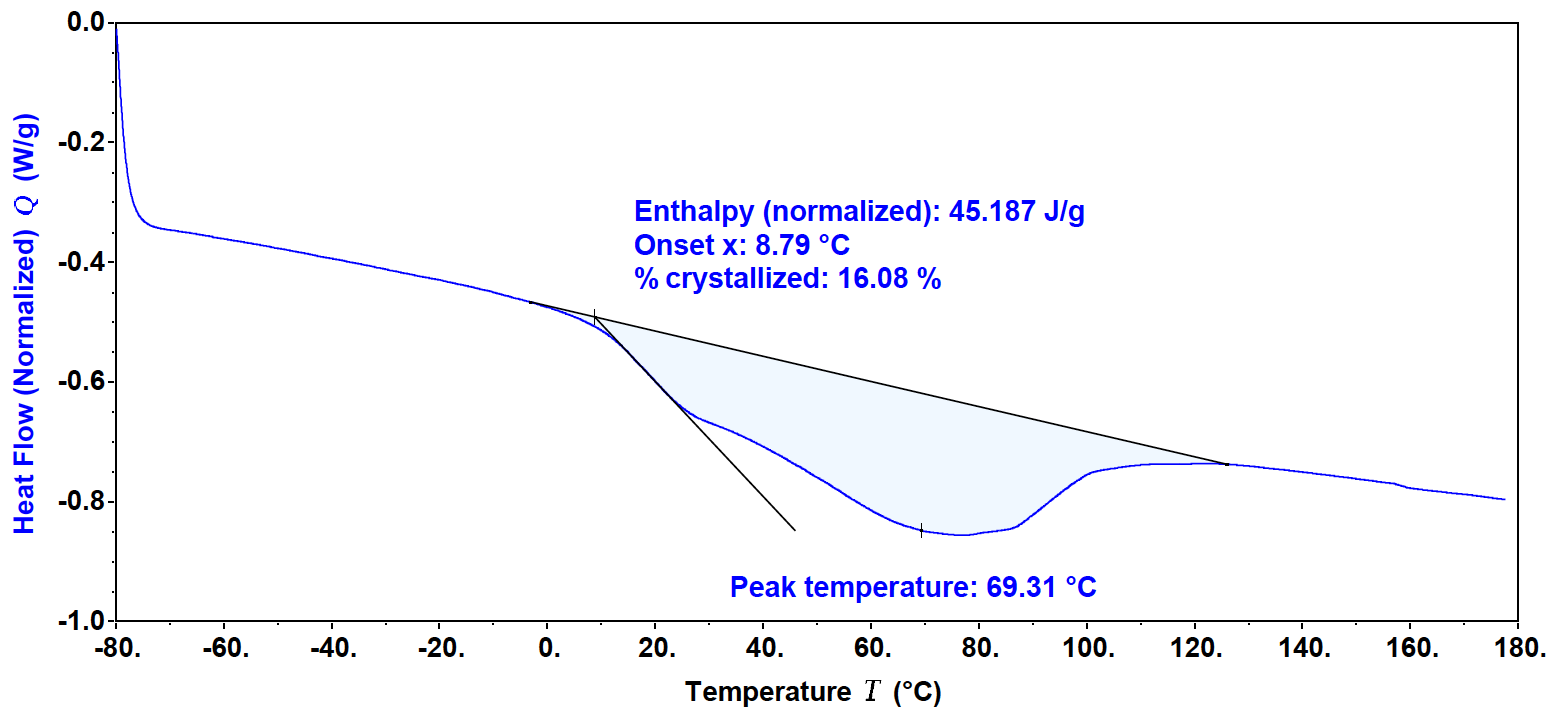
**

**Figure S73**. DSC trace of P3.

**
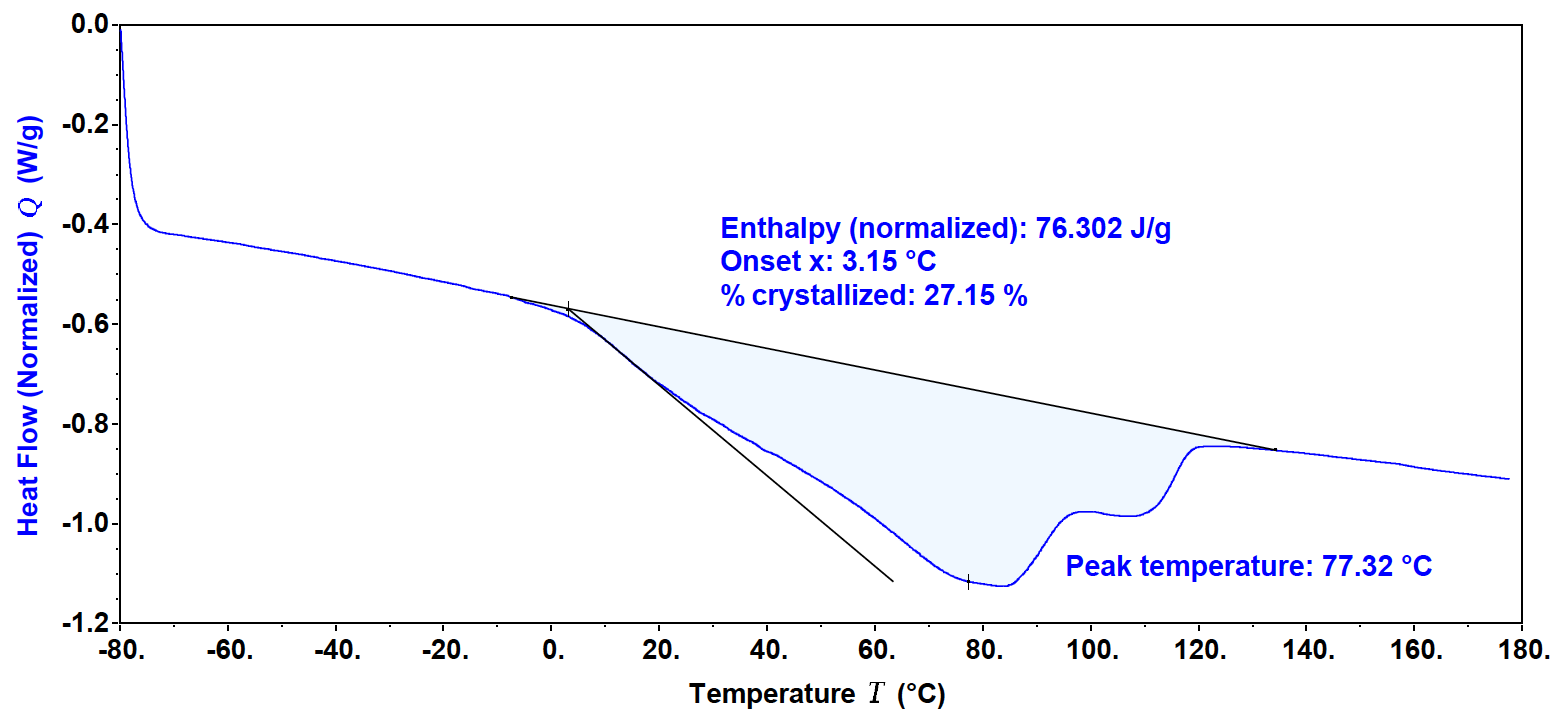
**

**Figure S74**. DSC trace of P4.

**
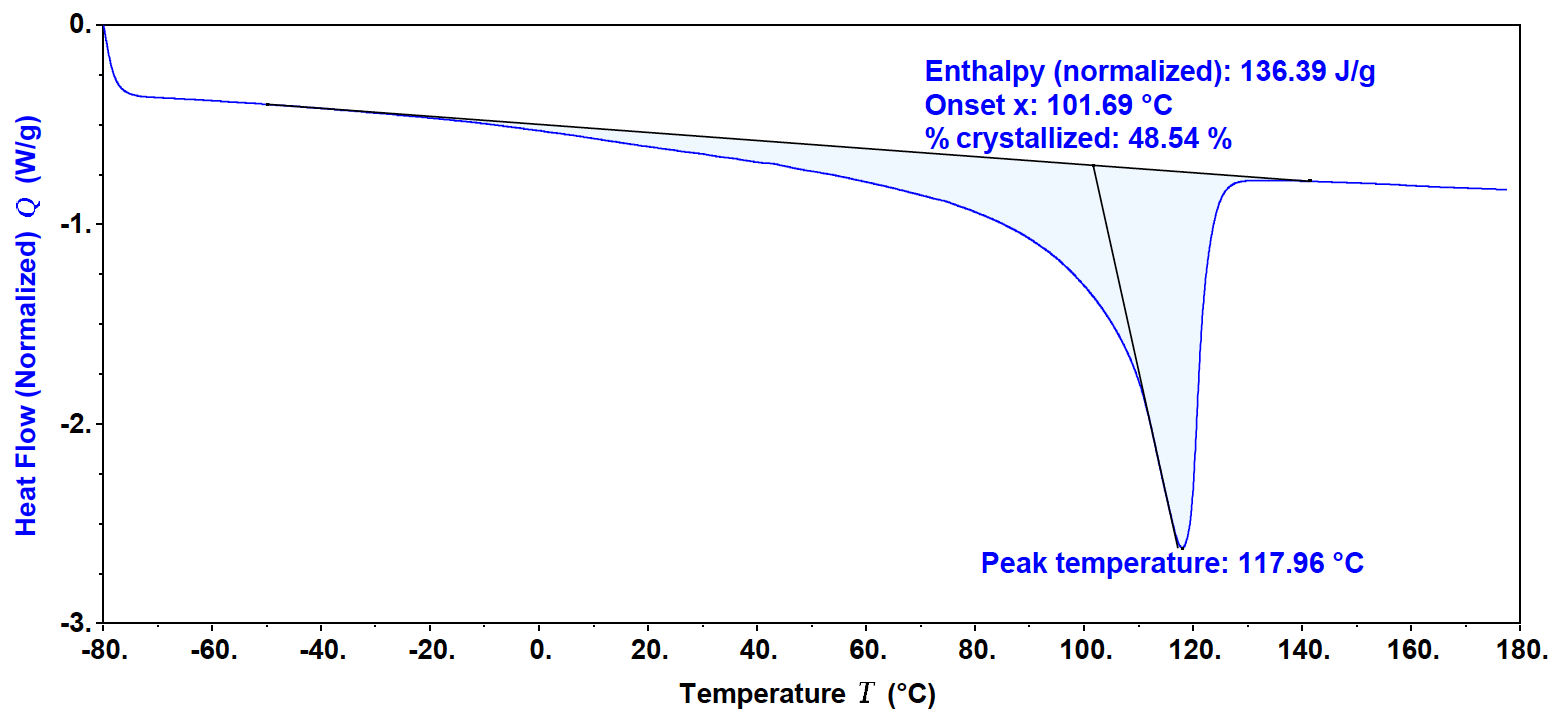
**

**Figure S75**. DSC trace of P5.

**
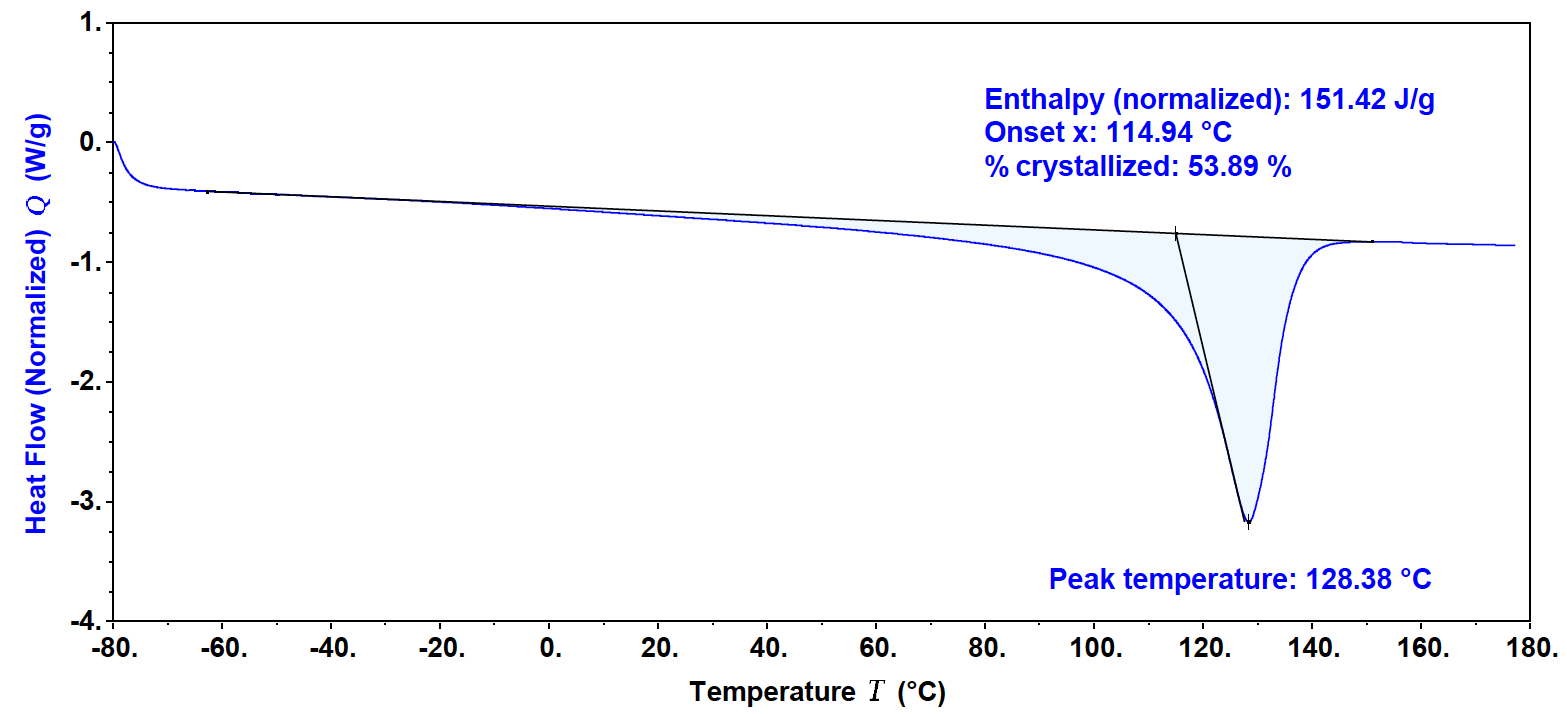
**

**Figure S76**. DSC trace of P6.

**
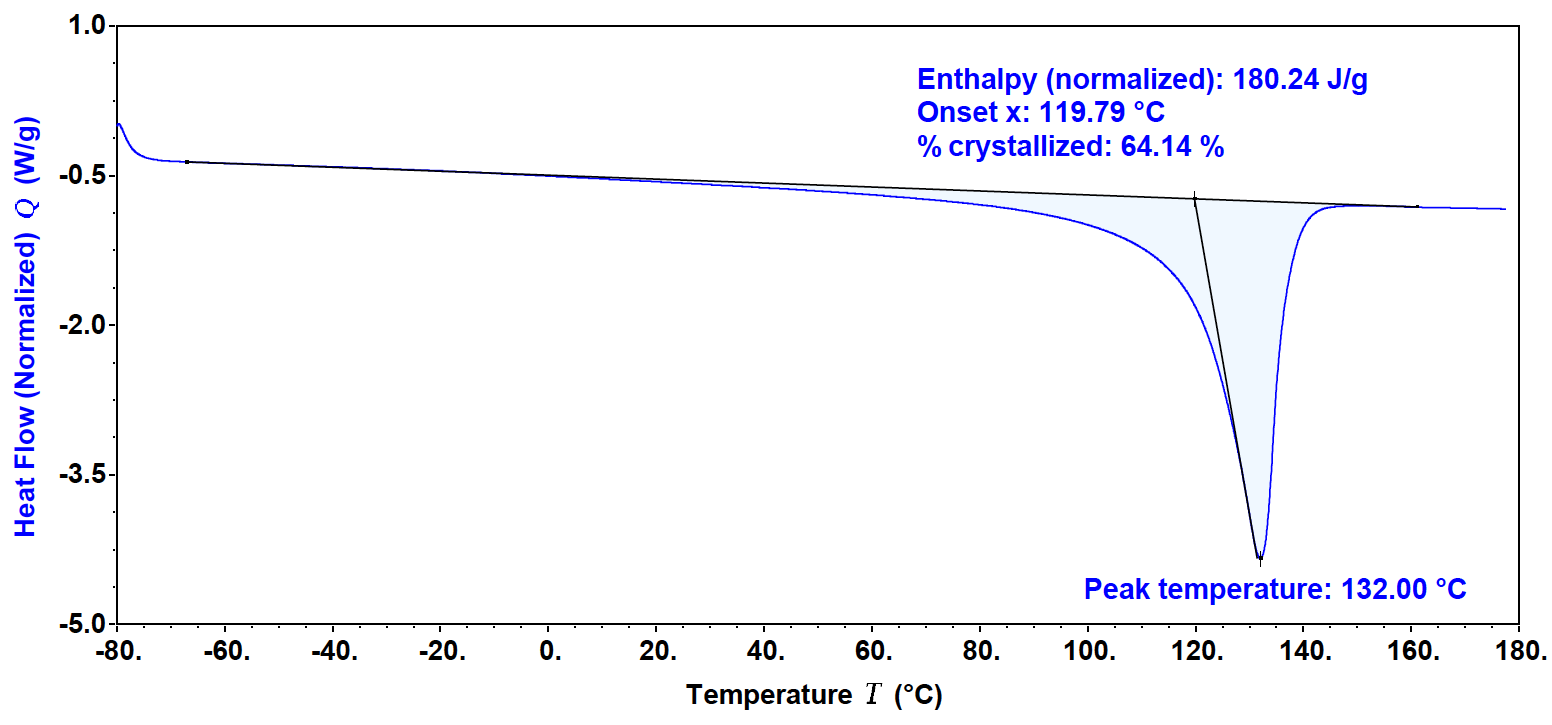
**

**Figure S77**. DSC trace of commercial HDPE.

**
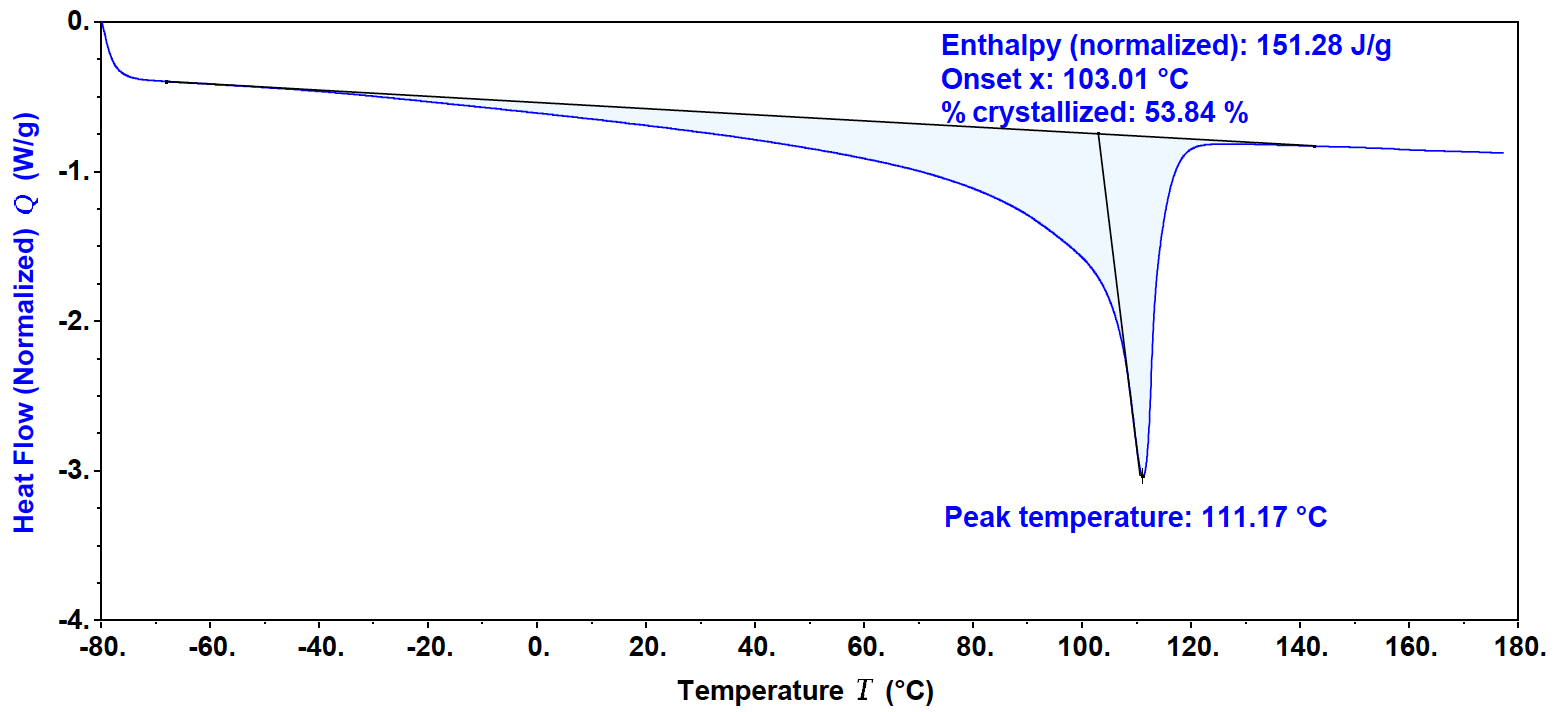
**

**Figure S78**. DSC trace of commercial LDPE.

**Figure S79**. DSC trace of commercial XLPE.

**
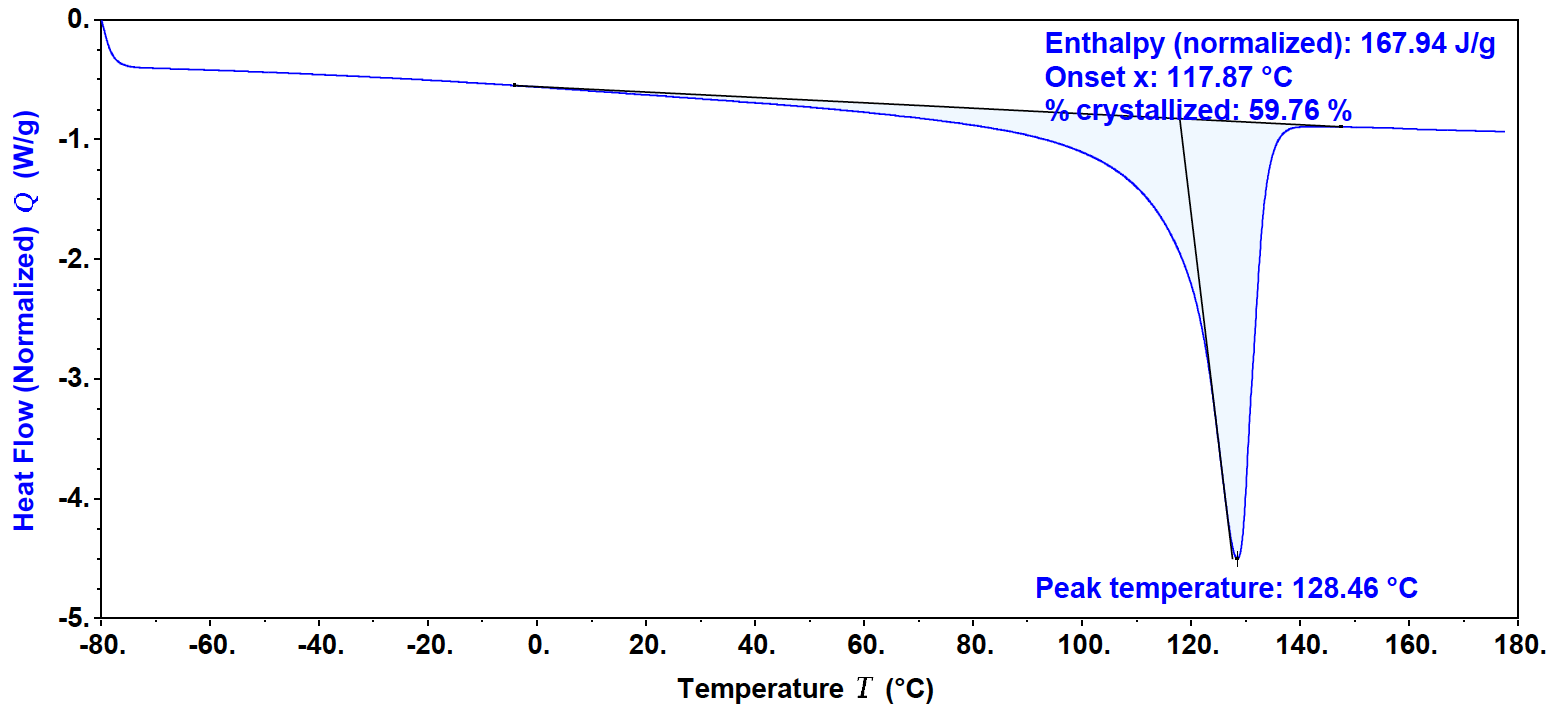
**

**Figure S80**. DSC trace of crosslinked PE-like polymer (P6-ReXL) from the repolymerization of degraded fractions of P6.

**Figure S81**. DSC trace of the crosslinked P6c (crosslinked P6).

**Figure S82**. DSC trace of the crosslinked PE-like polymer (P6c-ReXL) from the repolymerization of degraded fractions of P6c.

# **Reference**

(1) Liu, Y.; Cong, R.; Pan, Y.; Chen, M.; Xu, M. *Eur. Polymer. J.* **2024**, *203*, 112674.

(2) Na. Y.; Zhang, D.; Chen, C. *Poly. Chem.* **2017**, *8*, 2405.

(3) Guironnet, D.; Roesle, P.; Runzi, T.; Schnetmann, I. G.; Mecking. S. *J. Am. Chem. Soc.* **2009**, *131*, 422.

(4) Mevellec, L.; Evers, M.; Huet, F. *Tetrahedron* **1996**, *52*, 15103.

(5) Zhao, Y.; Rettner, E. M.; Harry, K. L.; Hu, Z.; Miscall, J.; Rorrer, N. A.; Miyake, G. *Science* **2023**, *382*, 310.
